# Convergence of Biofilm Formation and Antibiotic Resistance in *Acinetobacter baumannii* Infection

**DOI:** 10.3389/fmed.2022.793615

**Published:** 2022-03-24

**Authors:** Subhasree Roy, Goutam Chowdhury, Asish K. Mukhopadhyay, Shanta Dutta, Sulagna Basu

**Affiliations:** ^1^Division of Bacteriology, Indian Council of Medical Research (ICMR)-National Institute of Cholera and Enteric Diseases, Kolkata, India; ^2^Division of Molecular Microbiology, Indian Council of Medical Research (ICMR)-National Institute of Cholera and Enteric Diseases, Kolkata, India

**Keywords:** *Acinetobacter baumannii*, antimicrobial resistance, biofilm regulation, biofilm-associated infections, adult, paediatric, biofilm prevention

## Abstract

*Acinetobacter baumannii* (*A. baumannii*) is a leading cause of nosocomial infections as this pathogen has certain attributes that facilitate the subversion of natural defenses of the human body. *A. baumannii* acquires antibiotic resistance determinants easily and can thrive on both biotic and abiotic surfaces. Different resistance mechanisms or determinants, both transmissible and non-transmissible, have aided in this victory over antibiotics. In addition, the propensity to form biofilms (communities of organism attached to a surface) allows the organism to persist in hospitals on various medical surfaces (cardiac valves, artificial joints, catheters, endotracheal tubes, and ventilators) and also evade antibiotics simply by shielding the bacteria and increasing its ability to acquire foreign genetic material through lateral gene transfer. The biofilm formation rate in *A. baumannii* is higher than in other species. Recent research has shown how *A. baumannii* biofilm-forming capacity exerts its effect on resistance phenotypes, development of resistome, and dissemination of resistance genes within biofilms by conjugation or transformation, thereby making biofilm a hotspot for genetic exchange. Various genes control the formation of *A. baumannii* biofilms and a beneficial relationship between biofilm formation and “antimicrobial resistance” (AMR) exists in the organism. This review discusses these various attributes of the organism that act independently or synergistically to cause hospital infections. Evolution of AMR in *A. baumannii*, resistance mechanisms including both transmissible (hydrolyzing enzymes) and non-transmissible (efflux pumps and chromosomal mutations) are presented. Intrinsic factors [biofilm-associated protein, outer membrane protein A, chaperon-usher pilus, iron uptake mechanism, poly-β-(1, 6)-N-acetyl glucosamine, BfmS/BfmR two-component system, PER-1, quorum sensing] involved in biofilm production, extrinsic factors (surface property, growth temperature, growth medium) associated with the process, the impact of biofilms on high antimicrobial tolerance and regulation of the process, gene transfer within the biofilm, are elaborated. The infections associated with colonization of *A. baumannii* on medical devices are discussed. Each important device-related infection is dealt with and both adult and pediatric studies are separately mentioned. Furthermore, the strategies of preventing *A. baumannii* biofilms with antibiotic combinations, quorum sensing quenchers, natural products, efflux pump inhibitors, antimicrobial peptides, nanoparticles, and phage therapy are enumerated.

## Introduction

*Acinetobacter baumannii* occupies the upper echelon of the WHO priority pathogen list- “critical,” a position indicative of how important this nosocomial pathogen is, particularly when they are resistant to the “last resort” antibiotic, carbapenem (1–3). *Acinetobacter* causes a wide range of infections mostly acquired in clinical settings and is frequently associated with high morbidity and mortality rates (26–60%) (4, 5). The rate of mortality due to multidrug-resistant (MDR) and extensively drug-resistant (XDR) strains of *A. baumannii* infections is high and several outbreaks have been reported worldwide (5, 6). *A. baumannii* infections frequently occur in patients in ICUs on life-support systems prolonging their stay in hospitals and treatment failures are frequently encountered (7). Outside the hospital environment, *A. baumannii* has been isolated from a wide range of environmental samples including soil, aquatic environment, animals, humans, food items including raw vegetables, gardens, inanimate objects and even from body lice samples of homeless people, which serve as the reservoirs for this bacterium (8, 9). This species has now gone beyond hospitals and is being reported to cause community-acquired infections (10). Infections in both paediatric and adult populations indicate that as with other pathogens, the vulnerable are targeted (11). All these factors clearly indicate why *A. baumannii* is considered a critical pathogen.

Pathogens can grow and replicate even under unfavorable conditions. The tools that they use are diverse and many, sometimes as simple as the ability to persist in an environment. This ability to persist, particularly in adverse conditions such as hospitals where the use of antibiotics and disinfectants are high, gives *Acinetobacter* a clear advantage. *Acinetobacter* can survive in hostile environments (desiccation, antimicrobial therapies, nutrient unavailability) and can colonize biotic and abiotic surfaces for prolonged periods of time due to their ability to form complex structures called biofilms (12, 13). Biofilm formation is an important virulence mechanism and a hallmark characteristic of *A. baumannii*. Numerous microbial features (e.g., adhesins, capsular polysaccharides, surface appendages, virulence genes, resistance determinants), physicochemical factors (temperature, growth media, surface hydrophobicity, pH, oxygen concentration), and various other factors [biofilm-associated protein (Bap), the outer membrane protein A (OmpA), chaperon-usher pilus (Csu), iron uptake mechanism, poly-β-(1, 6)-N-acetyl glucosamine (PNAG), two-component system (BfmS/BfmR), PER-1], facilitate the formation and maintenance of the *A. baumannii* biofilms (13).

*Acinetobacter spp*. can form biofilm at both air-liquid and solid-liquid interface. The biofilm formation rate in *A. baumannii* at the solid-liquid interface is 80–91%, which is 3 times higher than other *Acinetobacter* species (5–24%) (14–16). In addition, these isolates are able to form biofilm at the air-liquid interface, known as pellicle, which increases the surface-associated motility of the bacterium. However, pellicle formation is a rare trait in *A. baumannii* and a limited number of genes are essential for the expression of this phenotype, but within the ACB-complex, pellicle formation was almost four times higher for *A. baumannii* than other *Acinetobacter* genospecies (15, 17). *Acinetobacter* infections may be more difficult to treat when forming a biofilm and may be readily transmissible from patient to patient, making outbreaks that are difficult to control. Hospital surfaces and surfaces of medical devices such as cardiac valves, artificial joints, ventilators, urinary or intravascular catheters, endotracheal tubes made of polystyrene, polypropylene, polytetrafluoroethylene, and glass are excellent for biofilm formation (18). Indwelling devices provide pathogens a mode of entry into the body, therefore patients admitted to the hospitals are at high risk of *Acinetobacter* infection as *Acinetobacter* can colonize on abiotic surfaces efficiently. A recent study suggested that the clinical isolates of *A. baumannii* have better ability to form biofilm on abiotic surfaces than non-clinical isolates. Therefore, the high capability of *A. baumannii* to colonize and form biofilm on abiotic surfaces is considered an important factor contributing to chronic and persistent infections in hospital settings (19). This subsequently enhances the risk of infectious diseases such as cystic fibrosis, periodontitis, bloodstream infections, urinary & respiratory tract infections, burn-wound infections, chronic non-healing injury, endocarditis, necrotizing fasciitis, etc. (18, 20, 21). Moreover, *A. baumannii* is able to maintain its virulence even after long periods of survival in the hospital environment, which could facilitate infections (22).

In addition to the ability to form biofilms, the deft with which *Acinetobacter* acquires antibiotic resistance genes and also transmits them provides the species with an additional advantage in hospitals where the use of antibiotics is always higher than in other environments. Studies have been showing horizontal gene transfer (HGT) of antibiotic resistance genes (carbapenemases, oxacillinases, metallo-beta-lactamases, or metal resistance genes) *via* conjugation, transformation, bacteria phage-mediated, nanotube-mediated, or *via* outer membrane vesicles (23–29). The mechanism of resistance is similar to other Gram-negative bacteria (GNB) which employ hydrolyzing enzymes or modifying enzymes, pumps to efflux antibiotics, and decreased entry of antibiotics. *Acinetobacter* is reported to possess numerous pumps and several enzymes (30). The ability to transmit these determinants is in no way lesser than the ability to acquire; transformation, conjugation, and outer membrane vesicles contribute to the spread of resistance determinants.

*Acinetobacter* utilizes its abilities to form biofilm and acquire antibiotic resistance determinants to evade the immune system, which provides one-thousand times more tolerance to antimicrobials by shielding the bacteria from treatment with antibiotics (13, 18, 21). Numerous studies have reported a constructive relationship between biofilm formation and antibiotic resistance in *A. baumannii* isolates (13, 21, 31, 32). Cells in the biofilm not only tolerate antibiotic pressure but biofilm formation enhance their ability to acquire foreign genetic material through lateral gene transfer that promotes their survival in presence of antibiotics (13, 31).

With the increasing importance of *A. baumannii* as a nosocomial pathogen, the factors that give this organism an advantage have been reviewed. The review gives a holistic view of presently available information about (i) the aspect of antibiotic resistance and biofilm production by *A. baumannii;* (ii) its clinical significance; (iii) how biofilm production and antibiotic resistance add to the challenges of biofilm-mediated nosocomial infection; and (iv) recent developments in potential approaches to prevent *A. baumannii* biofilm formation by disrupting components of the biofilm matrix.

## *A. baumannii* and the Tryst With Antibiotics

The genus *Acinetobacter* was discovered in 1911 by Beijerinck as *Micrococcus calcoaceticus* from the soil on a calcium acetate-mineral medium (33) and eventually, in the 1950s it became known as *Acinetobacter* (34). The genus *Acinetobacter* (the name came from the Greek word akinetos, i.e., non-motile), was originally suggested by Brisou and Prevot (35). Since then, more than 32 *Acinetobacter spp*. have been reported of which *A. baumannii* is most prevalent in clinical settings (4). Nosocomial infections, higher mortality among patients, and a higher degree of antimicrobial resistance are mostly encountered in *A. baumannii* compared to non-*baumannii* species (36).

### Evolution of Antimicrobial Resistance: The Timeline

**Figure 1:**
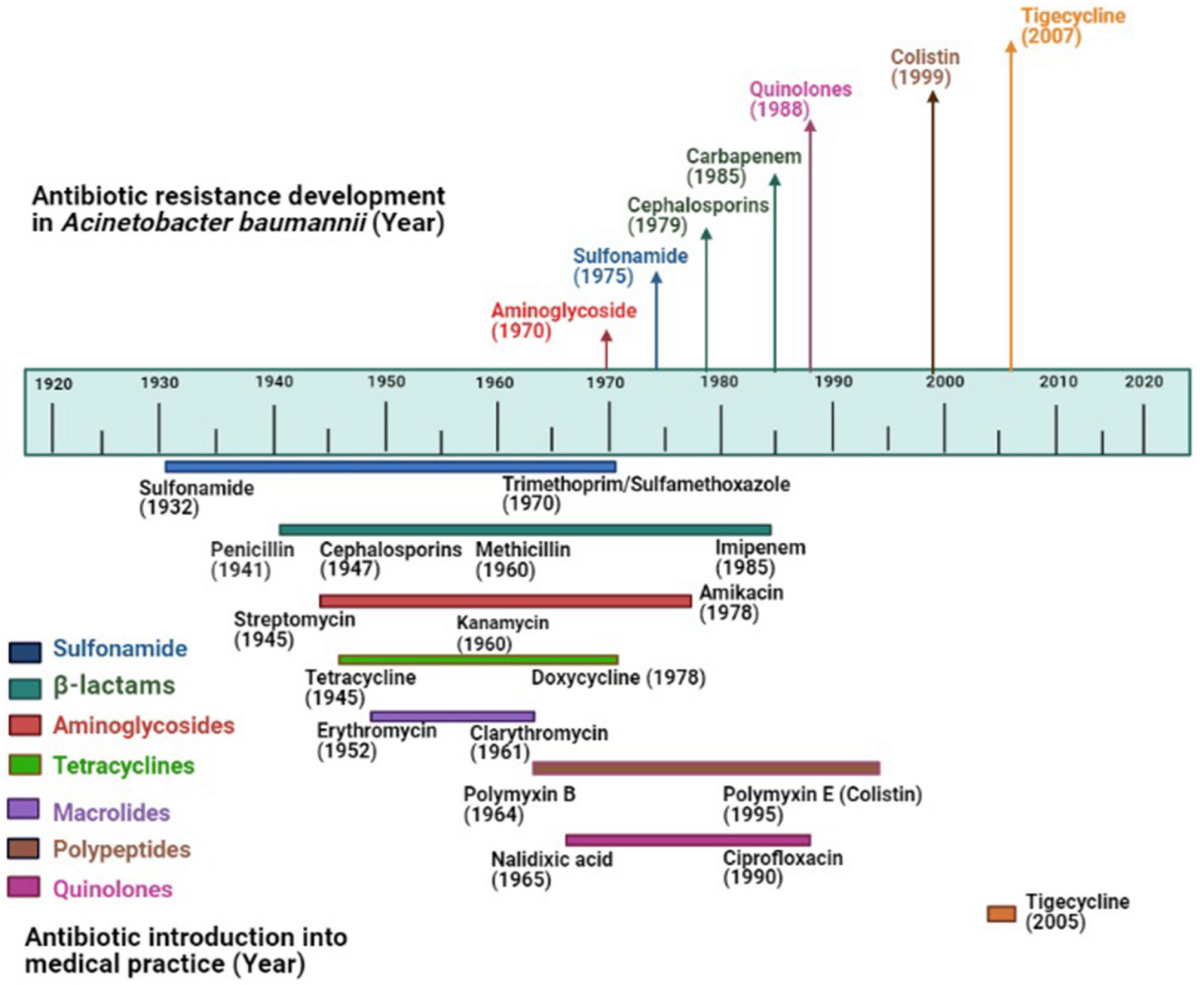
Evolution of antimicrobial resistance among *Acinetobacter baumannii*: Top portion of the diagram shows the year of the first report of antimicrobial resistance in *A. baumannii*; the lower portion shows the year of introduction of antimicrobials (approximate year) in the market where colored lines indicate different antimicrobial groups.

Up to the early 1970s, *Acinetobacter* strains showed susceptibility to most antibiotics including ampicillin, carbenicillin, nalidixic acid, and gentamicin (37). The resistance to sulfonamide, β-lactam, and aminoglycoside was noticed among *Acinetobacter* by the end of the 1970s as these drugs had already been used in clinical practice long before 1970 (38). Increased rate of resistance to many classes of antimicrobials was noticed among *Acinetobacter* in the 1980s including quinolones. Carbapenem resistance was first detected in 1985, the same year imipenem was discovered (39). Carbapenem-resistant *A. baumannii* is being frequently reported all over the world and the prevalence of carbapenem-resistant *A. baumannii* is high in developing countries including India, Pakistan, Chile, Korea, Portugal compared to the developed countries (40, 41). With the emergence of carbapenem resistance, as an alternative, colistin had been deployed to treat carbapenem-resistant bacteria, resulting in colistin resistance. The first report of colistin-resistant *Acinetobacter* came from the Czech Republic in 1999 (42). Since then, colistin resistance in *A. baumannii* has been reported worldwide including the USA, Europe, Spain, Korea, Iran, India (43). Apart from carbapenem and colistin resistance, resistance to tigecycline, the first member of the family of glycycline was reported in 2005 from Israel (44), the same year when it was approved by the US Food and Drug Administration (45). Tigecycline resistance has now been reported from all over the world (46). The timeline of the introduction of antimicrobials (approximate year) into the clinical practice and evolution of antimicrobial resistance in *A. baumannii* has been shown in Figure 1. For the creation of this figure, information was taken from different studies (47–49).

### Enzymatic Mechanisms of Resistance: Degradation and Modification

The emergence of multidrug-resistant *A. baumannii* has been attributed to its ability to rapidly accumulate resistance determinants as well as being well-suited for genetic exchange. Recent studies have shown that *A. baumannii* has natural competence to incorporate exogenous DNA and its genome has foreign DNA at high frequencies, implying frequent HGT in this pathogen (50–52). Therefore, *Acinetobacter* belongs to a unique class of GNB that are characterized as “naturally transformable” and a large number of β-lactamases have been identified in this human pathogen (53, 54). These β-lactamases, depending on their amino acid sequences, can be grouped into four classes (Ambler group A, B, C, and D) among which classes A, C, and D contain serine at the active site and class B have Zn in the catalytic site (55). Class A β-lactamases are capable of hydrolyzing cephalosporins, penicillins and are inhibited by clavulanic acid (56). Many class A β-lactamases are reported in *A. baumannii*, such as TEM, SHV, CTX-M, GES, PER, VEB, SCO, or KPC. Of these, most are broad-spectrum β-lactamases (Extended Spectrum β-lactamases, ESBLs) (SHV-5, TEM-92, CTX-M-2, CTX-M-15, PER-1, PER-2, PER-7, VEB-1, and GES-14) while TEM-1 and SCO-1 are narrow-spectrum (30, 57, 58). In *A. baumannii*, another mechanism of resistance to third-generation cephalosporins is the overexpression of chromosomally-mediated AmpC which is a class C β-lactamase (10, 30, 59). In several clinical isolates of *A. baumannii, ampC* gene was found to be transcribed from a strong promoter contained within a putative insertion sequence element (IS*Aba1*-like sequence), resulting in high resistance to ceftazidime (60). However, it was found that the exact contribution of ESBLs is complicated by the simultaneous presence of AmpC enzymes (53). Class D β-lactamases are also present in *A. baumannii* known as OXAs (oxacillinases) because they commonly hydrolyze isoxazolylpenicillin, oxacillin, much faster than benzylpenicillin (61). More than 400 OXA-type enzymes have been identified and many variants actually possess carbapenemase activity. OXA-23, OXA-24/40, OXA-58, OXA-143, and OXA-235 are examples of oxacillinases that are detected in *A. baumannii* and are able to hydrolyze carbapenems (10, 30, 62–64). Insertion of IS*Aba1* in the *bla*_OXA−23_ promoter sequence has been reported to be associated with overexpression of *bla*_OXA−23_, *bla*_OXA−51_, or *bla*_OXA−58_ in *A. baumannii* (65, 66). Class B β-lactamases which are very different from the other classes and known as MBLs (metallo-β-lactamases), can be inhibited by EDTA as they possess Zn at their active site (30). Several MBLs have been reported in *A. baumannii* such as IMP (imipenemases), VIM (Verona integron-encoded MBL), SPM (Sao Paolo MBL), SIM (Seoul imipenemase), GIM (imipenemase from Germany), and NDM (New Delhi MBL) (30, 67–71). These MBLs are the primary reason for carbapenem resistance in *A. baumannii* along with oxacillinases.

Apart from hydrolysis of antimicrobials by β-lactamases, enzymatic modification of the antibiotics is another mechanism of enzymatic resistance in *A. baumannii*. One of the best examples is the presence of three different aminoglycoside-modifying enzymes (acetyl transferases, nucleotidyl transferases, and phosphotransferases) which modify amino or hydroxyl- groups of the aminoglycosides (67).

### Non-enzymatic Mechanisms of Resistance: The Active Pumps and More

Most GNB including *A. baumannii* also possess several non-enzymatic mechanisms of resistance which include efflux pumps, modifications of drug binding sites, and permeability defects.

To date, different categories of efflux pumps have been identified in *A. baumannii*: RND-family (resistance-nodulation-division), MFS-family (major facilitator superfamily), MATE-family (multidrug and toxic compound extrusion), and SMR-family (small multidrug resistance). The RND system more actively participates in antimicrobial resistance in *A. baumannii* and this family includes the AdeABC, AdeIJK, and AdeFGH efflux pumps (72). These efflux pumps are controlled by certain regulators such as AdeRS (two-component system), AdeL, and AdeN (72). Some other efflux pumps detected in *A. baumannii* include MATE-family (AbeM and CraA), MFS-family (AmvA, AbaF, and AbaQ), and SMR- family (AbeS) (72). tet(A), tet(B), and tet(G) are specific transposon-mediated efflux pumps also detected in *A. baumannii* (73).

Random point mutations, which are an important mechanism of bacterial resistance, alter the target site of antibiotic binding. The examples of such mechanisms among *A. baumannii* are (i) fluoroquinolone resistance due to spontaneous mutations in gyrase and topoisomerase IV encoding genes, i.e., *gyrA, gyrB*, and *parC, parE*; (ii) rifampin resistance due to point mutations in the RNA polymerase encoding gene *rpoB*; (iii) colistin resistance due to mutations in PmrAB two-component system and *lpxA, lpxC, lpxD* genes; and (iv) aminoglycoside resistance due to mutations in 16S ribosomal RNA gene *armA* (10, 30, 67, 74). Moreover, carbapenem resistance in *A. baumannii* is also associated with mutations in Penicillin Binding Protein PBP-2 (30, 67, 75).

**Figure 2:**
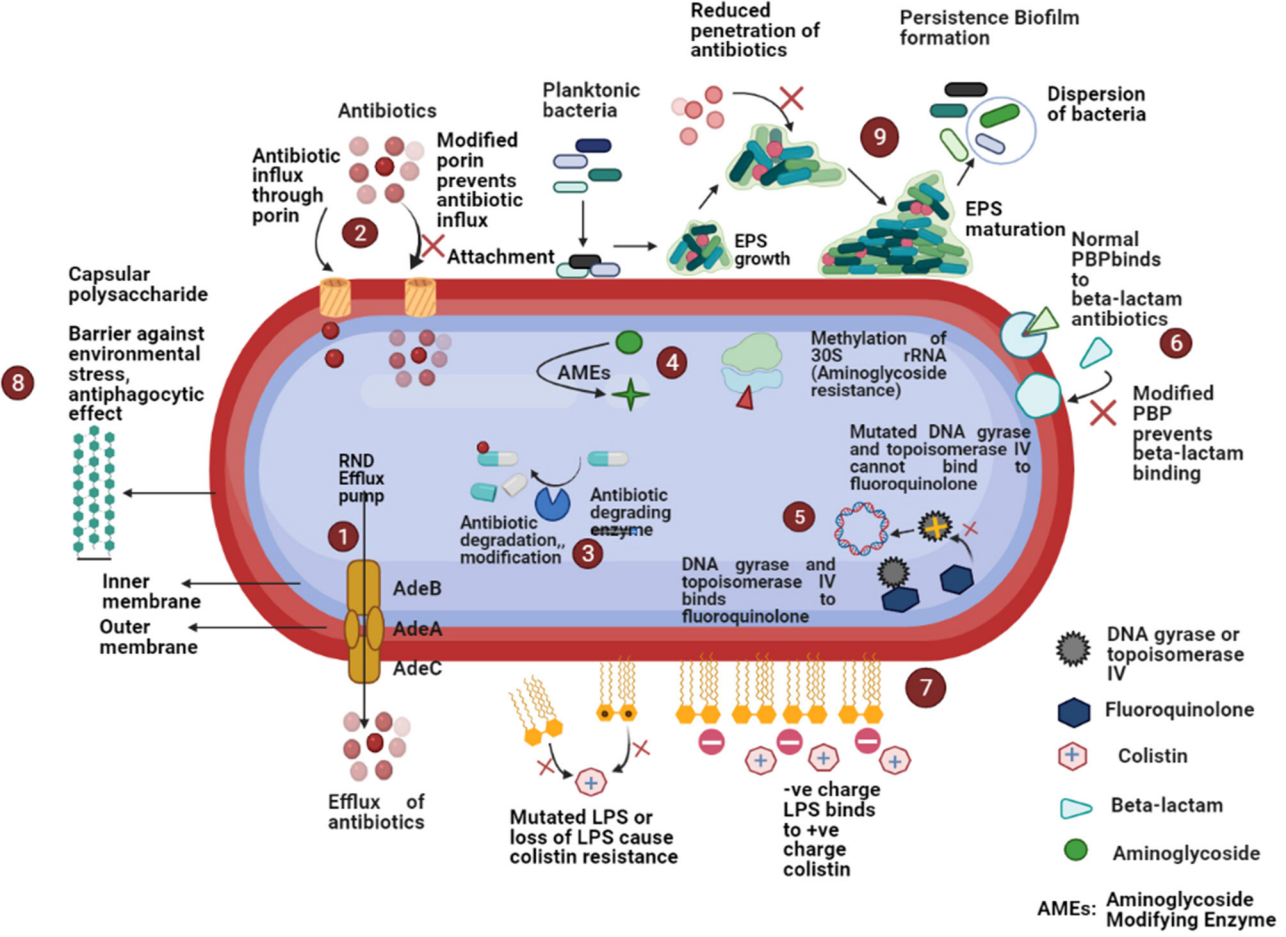
Schematic diagram of different antimicrobial resistance mechanisms in *A. baumannii*: (1) increased expression of efflux pumps that expel out antibiotics from the bacterial cell; (2) reduced expression of porin or porin loss results in the decreased antibiotic entry; (3) β-lactamases cause enzymatic inactivation of antibiotics; (4) aminoglycoside modifying enzymes decrease the affinity of aminoglycoside antibiotics for ribosomal subunit or methylation of 30S rRNA decrease the binding of aminoglycosides; (5) mutations in topoisomerase IV and DNA gyrase decrease the binding of fluoroquinolones; (6) modification of penicillin-binding-proteins (PBPs) prevent the bindings of β-lactams; (7) modification of lipopolysaccharides (LPS) cause decreased binding of colistin; (8) presence of capsular polysaccharide acts as a barrier against environmental stress, anti-phagocytic effect, etc.; (9) ability to form biofilm cause high antimicrobial resistance.

Porins also play a significant role in antimicrobial resistance among *A. baumannii*. Decreased expression of several porins (Omp22–23, Omp33–36, Omp37, Omp43, Omp44, Omp47, OmpA, and CarO) has been noted in carbapenem-resistant *A. baumannii* (30, 76). A schematic diagram of the several antimicrobial resistance mechanisms has been depicted in Figure 2.

## *A. baumannii* and Biofilm: Involvement of Intrinsic and Extrinsic Factors

Biofilm is a complex multicellular three-dimensional structure of the assembled population of bacterial cells which is enclosed within an exopolymer matrix called extracellular polymeric substance (EPS) (77). The EPS comprises nucleic acids, carbohydrates, proteins, and other macromolecules. In the EPS matrix, the most abundant carbohydrates are glucose, galactose, and mannose followed by fucose, arabinose, xylose, rhamnose, galacturonic acid, and *N-*acetyl-glucosamine. The complexity of the biofilm is enhanced by the presence of extracellular proteins which stabilize the biofilms and extracellular DNA (eDNA) secreted by the cells (77).

Formation of the biofilms, also known as the biofilm cycle, involves six major stages: (i) reversible attachment of planktonic bacteria with surfaces followed by settlement; (ii) proliferation and aggregation of the adherent bacteria (irreversible attachment); (iii) formation of microcolonies which either look like mushrooms or towered structures; (iv) initiation of quorum sensing (communication pathway between cells) at a critical cell density facilitating biofilm formation, positioning of cells in the microcolonies, formation of water channels within the biofilm and detachment of cells; (v) biofilm maturation (maximum antibiotic tolerance can be observed at this stage due to the presence of thick polysaccharide matrix surrounding them); and (vi) detachment and dispersion of cells to colonize in another location (78).

## Intrinsic Factors Associated With *A. baumannii* Biofilm Formation: The Tools Within

**Figure 3:**
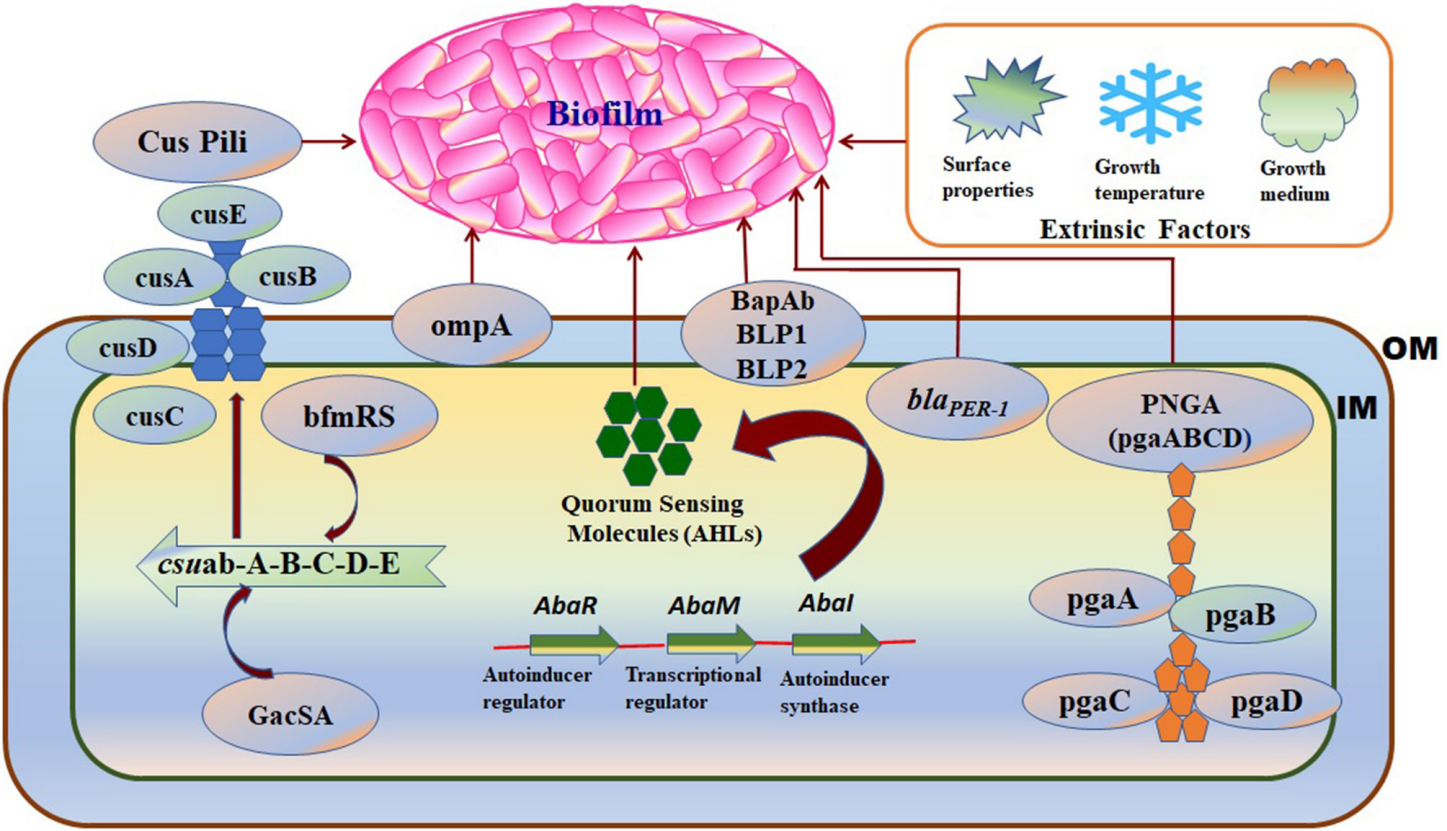
A schematic diagram representing the intrinsic factors (genes) and the extrinsic factors that regulate biofilm formation in *A. baumannii:* OM, Outer membrane; IM, Inner membrane. **Intrinsic factors:** PNAG, Poly-(1–6)-N-acetylglucosamine; Csu, Chaperon/usher pilus system; OmpA, Outer membrane protein A; *bla*__*PER*_−_ 1, Beta-lactamase PER-1; bap-Ab, *A. baumannii* biofilm-associated protein; AHLs, N-acyl homoserine lactones; **Extrinsic factors:** surface properties, growth temperature, and growth medium.

Biofilm formation in *A. baumannii* on biotic and abiotic surfaces is regulated and influenced by several intrinsic factors such as virulence genes or proteins, cellular structures, and phenotypic or genotypic features. The factors associated with biofilm formation and regulation in *A. baumannii* are summarized in Table 1 and shown in Figure 3.

**Table 1:** Factors implicated in *Acinetobacter baumannii* biofilm formation and regulation.

**Effectors**	**Gene determinants**	**Functions**	**References**
*A. baumannii* Biofilm-associated protein (BAP)	*bap-Ab*	Bap is a surface exposed protein, plays an important role in cell-cell adhesion, interactions, biofilm formation, and maturation.	(79, 80)
Poly-β-(1, 6)-N-acetlyglucosamine (PNAG)	*pgaA, pgaB, pgaC, and pgaD*	PNAG is a polymeric exopolysaccharide essential for cell–cell adherence, biofilm formation, and thickness of biofilm.	(81, 82)
Beta-lactamase PER1	*bla_*PER*−1_*	*bla_*PER*−1_* is a broad-spectrum of β-lactamase gene responsible for adhesion and biofilm formation to both biotic and abiotic surfaces.	(83)
Csuab-A-B-C-D-E. chaperone-usher pilli assembly system	*csuA, csuB, csuC, csuD and csuE, bfmRS (bfmR and bfmS), gacSA*	Cus pili are surface homo or heteropolymer protein structures, play a key role in the adhesion, pili production and assembly, biofilm formation, and maintenance on abiotic surfaces.	(84, 85)
Outer membrane protein A	*ompA*	The *OmpA* are key virulence factors in adhesion, invasion, biofilm formation, and cytotoxicity in biotic surfaces.	(86)
Quorum Sensing (QS)	*abaR, abaM, and abaI*	QS produces the signaling molecules, autoinducers to maintain bacterial cell-to-cell communication, population density, synchronized behavior, and interaction. QS is also responsible for activation and regulation of gene expression of virulence factors, motility, plasmid transfer, drug resistance, and biofilm formation.	(87, 88)

### Biofilm Associated Protein (Bap)

Biofilm-associated protein (Bap) is a high-molecular-weight protein (8,620 amino acids), essential for cell-cell interactions, biofilm formation, and maturation on various surfaces including polypropylene, polystyrene, and titanium (89). BAP was first identified in *A. baumannii* by Loehfelm et al. and is identical to the *Staphylococcus aureus* Bap protein (79). *A. baumannii* Bap mainly targets carbohydrates in the host cells and is secreted *via* a type I secretion system. Several studies have documented the presence of Bap in *A. baumannii* strains and its association with strong biofilm formation (80, 90). Loehfelm et al. showed that Bap protein increases adherence to both normal human neonatal keratinocytes and bronchial epithelial cells (79). Many Bap-like proteins, BLP1 and BLP2 are also harbored by *A. baumannii* which coordinately contribute toward mature biofilm formation and adhesiveness to epithelial cells in a similar fashion as Bap (80).

### Poly-β-(1, 6)-N-Acetlyglucosamine (PNAG)

Poly-β-(1, 6)-N-acetlyglucosamine (PNAG) is one of the major components of polysaccharides and is important for the development of biofilm in both GPB and GNB (81). PNAG is encoded by a cluster of four genes (*pgaA, pgaB, pgaC*, and *pgaD*) (82) which share similarities with *E. coli* and *Y. pestis* (91). *pgaA* plays an important role in protein-protein interaction and contains a porin domain which facilitates PNAG translocation through the outer membrane, *pgaB* involves in PNAG exportation *via* the outer membrane lipoprotein, *pgaC* helps in the synthesis of PNAG and *pgaD* restricts the cytoplasm and promotes *pgaC* in the synthesis of PNAG (91). Numerous studies showed PNAG is essential for maintaining the integrity of *A. baumannii* biofilms in a more dynamic and stressful environment (81, 91).

### PER-1 β-lactamase

*Acinetobacter baumannii* adhesion and biofilm formation in both biotic and abiotic surfaces are enhanced by the presence and the expression of the *bla*_PER−1_ gene which is a class A extended-spectrum β-lactamase (92). Several studies found that increased cell adhesiveness and biofilm formation was higher in strains harboring the *bla*_PER−1_ gene than in those that do not harbor this genetic trait (83, 92, 93). However, Bardbari et al. reported no relationship between biofilm formation and production of *PER*-1 β-lactamase (94). Therefore, the presence of *bla*_PER−1_ probably increases the adhesion property of cells carrying this gene but does not necessarily contribute to biofilm formation.

### *Csuab-A-B-C-D-E* Chaperone-Usher Pilli Assembly System

Chaperon–Usher secretion (CUS) system is required for bacterial attachment on abiotic surfaces that results in microcolony formation and development of biofilm (95). Csu pili are poly-cistronic in nature, adhesive surface organelles that consist of a tip fibrillum and adhesion protein (84). *A. baumannii* strains produce type I Csu pili that is encoded by an operon: *Csuab-A-B-C-D-E*. Furthermore, the expression of the *Csuab-A-B-C-D-E* operon in *A. baumannii* is regulated by a two-component system BfmRS where BfmR acts as a response regulator and BfmS acts as a sensor kinase (85, 96). Moreover, a second two-component system GacSA controls the Csu pilli gene expression and is indirectly involved in biofilm formation in *A. baumannii* (97). Other putative chaperone usher pili systems and Pap pili systems, which are homologous to the P pili of *E. coli*, have also been implicated in *A. baumannii* biofilm formation and maintenance (98).

### Outer Membrane Proteins

Porins are the outer membrane proteins (Omps) that modulate cellular permeability, have an essential role in adaptation, environmental communication, and also play an important role in microbial virulence through drug exclusion mechanisms across the outer membrane channels (86). The outer membrane protein A (OmpA) is a well-recognized and well-characterized virulence factor of *A. baumannii* and is necessary for the development of robust biofilms on abiotic surfaces (86). The two-component system BfmS/R regulates biofilm formation, pilus, and OmpA expression, along with serum sensitivity (99). Another outer membrane protein of *A. baumannii* is the Omp 33–36 kDa protein which acts as a channel for water and is associated with resistance to carbapenem antibiotics. Moreover, knockout of *omp 33–36* gene in *A. baumannii* strains had defective growth rate and significantly reduced capability of adherence, invasion, biofilm formation, and cytotoxicity in biotic surfaces (100).

### Quorum Sensing (QS)

Quorum sensing (QS) is a cell-to-cell communication process that depends on the bacterial population density. Several small diffusible signaling molecules are involved in QS which activate the expression of genes that control a variety of functions such as virulence, motility, biofilm formation, bioluminescence, and sporulation (87, 101). These diffusible signaling molecules termed autoinducers include oligopeptides in GPB and N acyl-homoserine lactones (AHLs) in GNB (102). The predominant AHL produced by *A. baumannii* is 3-hydroxy-C12- homoserine lactone. The QS system in *A. baumannii* is regulated by a two-component system, AbaI/AbaR which is homologous to the typical LuxI/LuxR system found in GNB. *abaI* encodes the autoinducer synthases which catalyze the synthesis of AHL and AbaR that functions as receptor proteins for AHLs (103). A previous study had shown that mutation of the AHL synthase AbaI affects the surface-associated motility and biofilm formation in *A. baumannii*. A functional QS system is required for surface-associated motility and robust biofilm formation in *A. baumannii* ATCC®17978 (104). In addition, another gene named *abaM* is an uncharacterized member of the RsaM protein family located between *abaR* and *abaI*, has been found to play a key role in regulating *A. baumannii* QS, virulence, surface motility, and biofilm formation (88). The expression and upregulation of another two-component system BfmS/R has also been linked to the QS molecules that enhance the ability of *A. baumannii* to form biofilm on abiotic surfaces (105). Quorum sensing deficiency causes thinner biofilm formation and lower EPS production, thereby increasing the susceptibility to antibiotics.

### Efflux Pumps

Efflux pumps are membrane proteins, that can extrude a wide group of substrates, including antibiotics, detergents, dyes, toxins, and waste metabolites. Several studies suggest that efflux pumps play major roles in biofilm formation and maturation by different mechanisms: efflux of EPSs and quorum quenching (QQ) molecules to facilitate biofilm matrix formation; indirect regulation of genes involved in biofilm formation and efflux of antibiotics or metabolic intermediates (106, 107). There are three types of RND efflux pumps associated with *A. baumannii*: AdeABC, AdeFGH, and AdeIJK. Yoon et al. reported that mutant strains of AdeABC, AdeFGH, and AdeIJK efflux pumps have significantly reduced biofilm formation in comparison with the wild-type strain. Therefore, biofilm formation in *A. baumannii* requires expression of efflux pump genes to initiate and maintain biofilm. Another study reported that the mutation of AdeABC and AdeIJK efflux genes were associated with lower expression of several pilus system-encoding proteins CsuA/B, CsuC, and FimA. These proteins play a key role in the initial stages of adhesion, surface colonization, and biofilm formation in *A. baumannii* (108). Richmond et al. presented that knockout of AdeAB efflux pumps in *A. baumannii* mutant strain caused significant reduction in biofilm formation on mucosal tissue compared with wild type strain. Therefore, the over-expression of AdeABC and AdeIJK efflux pump regulate the expression of pilus genes and biofilm production, and altered membrane composition in *A. baumannii* (108, 109). The third RND-type efflux pump AdeFGH is regulated by a LysR-type AdeL transcriptional regulator system. The over-expression of this efflux pump confers multidrug resistance and is linked to the synthesis and transport of autoinducer molecules during biofilm formation in *A. baumannii*.

## Extrinsic Factors Associated With *A. baumannii* Biofilm Formation: the External Influences

Certain environmental factors that affect *A. baumannii* biofilm formation are summarized in Table 1 and shown in Figure 3.

### Surface Property

Several factors such as roughness, physicochemical properties of a surface, and the presence of biological materials influence the attachment of *A. baumannii* to abiotic surfaces and the formation of biofilm (110). The ability of *A. baumannii* to form mature biofilms on polypropylene, polystyrene, titanium, and other medical-associated devices has been associated with several factors including pH, ionic composition, and biomaterial of protein adsorption (111). The presence of biomaterial such as blood, tears, urine, saliva, interstitial fluid, wound cultures and respiratory secretions influence the attachment of bacteria to its surface and promote the formation of biofilm (112). Polycarbonate surfaces are known to develop statistically more biofilm mass than glass, rubber, porcelain, and polypropylene (110). Latex catheters are low-priced and have more elasticity but are prone to bacterial adhesion and biofilm formation. Hence, silicone catheters are preferred over latex catheters (113).

### Growth Temperature

Temperature also has an effect on biofilm formation. *A. baumannii* successfully survived at −20 to 44°C (114). However, different studies have been reported different optimum temperatures for biofilm formation in *A. baumannii* including 30°C at pH 7 in a medium containing sodium chloride or 25°C (115, 116). Another study showed that biofilm formation in *A. baumannii* on plastic surfaces was high at 28°C due to the upregulation of certain biofilm-associated proteins (BAPs), Csu pili, and iron-uptake proteins (111).

### Growth Medium

The growth medium is also a factor that affects biofilm formation. It has been reported that a static environment with high nutrient containing medium (Tryptic Soy Broth or Brain Heart Infusion Broth) and supplemented with glucose, carbon, and cation sources (Na + sodium, Ca^2+^ calcium, Fe^3+^ ferric ion) influences the formation of *A. baumannii* biofilm than in hydrodynamic environment (117). These modulatory properties of medium and supplemented sources also influence the structural and mechanical properties of *A. baumannii* biofilms by lowering stiffness and increasing adhesiveness (117). However, clinical isolates of *A. baumannii* showed a significant reduction in adhesiveness and biofilm formation in the presence of an iron-chelating agent and ethanol on abiotic surfaces (83, 118).

## *A. baumannii* Biofilms and Antibiotic Resistance: A Dangerous Liaison

### Antimicrobial Resistance by Biofilm Cells: Understanding the Mechanisms

**Figure 4:**
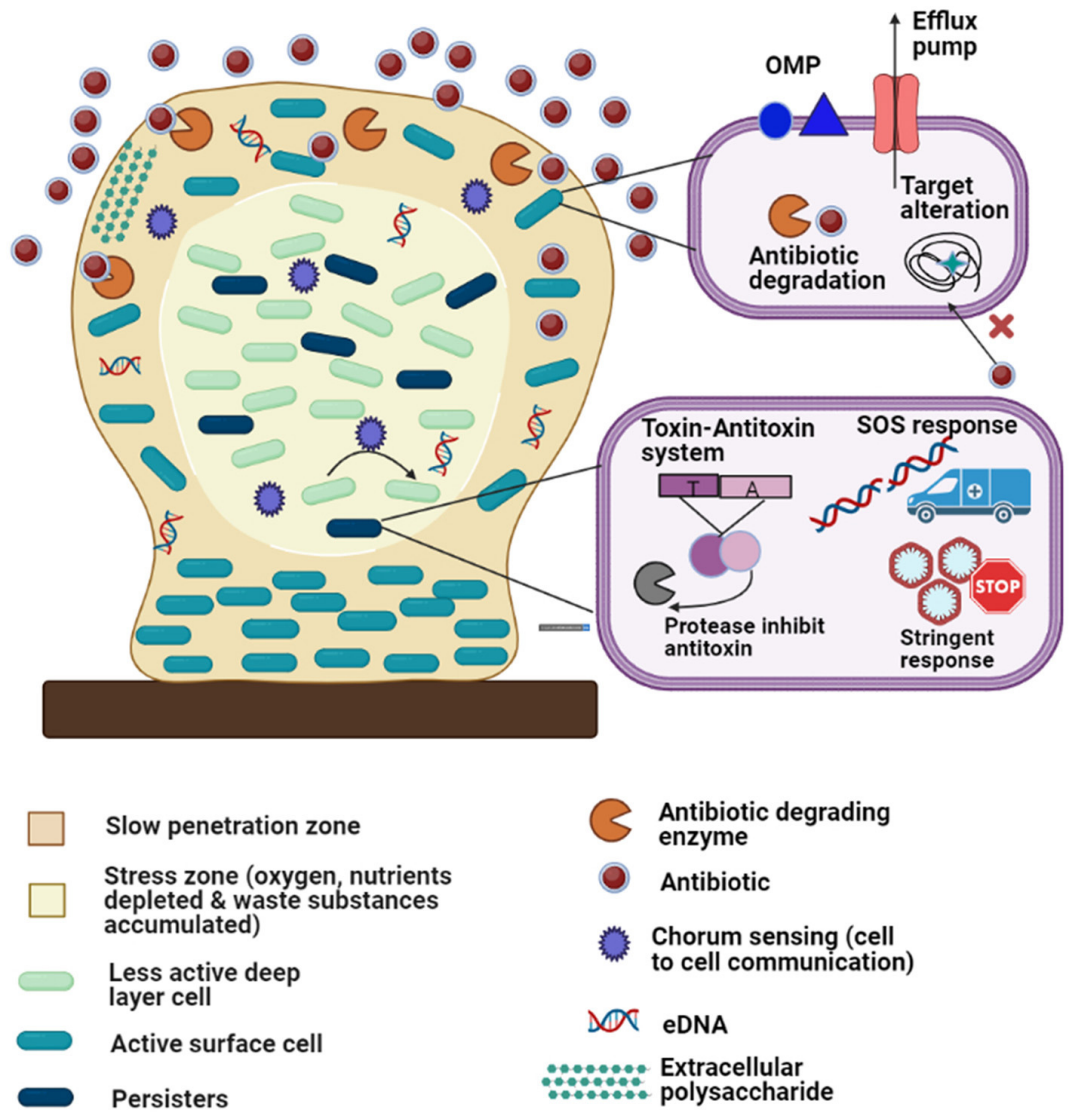
Diagrammatic representation of the antibiotic resistance mechanisms of biofilm-embedded bacterial cells: The biofilm is attached to a biotic or abiotic surface (brown rectangle). Development of persister cells (dark green) and less active deep layer cells (light green) in the stress zone (the core of the biofilm, light cream color) where fewer nutrients are available. The various resistant mechanisms depicted in the figure are as follows: (1) matrix exopolysaccharides cause slow penetration of antibiotics; (2) extracellular DNA (eDNA); (3) multidrug efflux pumps; (4) outer membrane protein; (5) antibiotic degrading enzymes and target modifications (6) quorum sensing; (7) stress responses (oxidative stress response, stringent response, etc.); (8) toxin-antitoxin system and (9) SOS responses.

The term “biofilm resistance” signifies the survival of cells embedded within biofilms for long periods of time in presence of antimicrobials. Biofilm resistance does not indicate that biofilm cells show increased MIC compared to antibiotic-resistant planktonic cells (119). Biofilms are better equipped to evade antimicrobials than planktonic cells because biofilms are not easily destroyed by antimicrobials. Factors that are responsible for biofilm resistance and explain better survival of biofilm cells compared to planktonic cells in presence of antimicrobials are described below (Figure 4).

### Diffusion Inhibition of Antibiotics: Restricted Entry

Inhibition of antibiotic diffusion within biofilms can be attained *via* several mechanisms such as the presence of EPS matrix that serves as the diffusion barrier for antibiotic entry, antibiotic degradation with the help of production of exoenzymes (β-lactamases, β-galactosidase), metal immobilization or chelation, extracellular signaling, a mutation in the antibiotic target site and oxidation-mediated inactivation of antibiotics (120). These processes can lower antibiotic concentration to sublethal levels, leading to the selection of antibiotic-resistant cells within a biofilm. In addition, negatively charged eDNA facilitates the lowering of antibiotic concentrations within biofilms by binding to positively charged antibiotics such as aminoglycosides and gathers antibiotics up to 25% of its weight (121). eDNA can also enhance antibiotic resistance within biofilms by chelating cations such as Mg2+ and creating a cation-depleted environment leading to activation of two-component systems which are linked to AMR (121). It facilitates the transfer of antibiotic resistance genes within biofilms.

eDNA not only comes from lyzed cells but is also actively secreted which indicates that eDNA has an important role in biofilm formation. Its negative charge works as a repulsive force in the initial attachment, but when the distance between cell and surface becomes a few nanometers, eDNA interacts with receptors on the substratum surface to facilitate adhesion (122). Sahu et al. characterized eDNA from a multidrug-resistant clinical strain of *A. baumannii* and demonstrated its role in *in vitro* biofilm formation on abiotic surfaces. They supplemented biofilms with eDNA in various preparations, for example, whole-cell lysate, cell-free supernatant, MV suspension, and purified eDNA to mimic its natural availability during growth. Their experiment showed that eDNA supplemented in any of the given forms was able to augment the biofilms on polystyrene microtiter surface significantly (224.64%), whereas biofilm inhibition was 59.41% after DNase I treatment: confirming that eDNA facilitates biofilm formation in *A. baumannii* (123).

### Heterogeneity and Decreased Growth Rate: The Environment Within Matters

The growth rate of organisms embedded in biofilms varies due to oxygen and nutrient depletion especially inside the microcolonies and in the deep cell layer, leading to gradients of nutrients that result in different growth rates such as fast/slow-growing cells, dormant cells, persister cells. These differences in growth rates cause population heterogeneity within the biofilms (124). The slow growth of the bacteria has been observed in mature biofilms (125). Generally, antimicrobials kill rapidly growing cells. Therefore, slow growth undoubtedly contributes to biofilm resistance (126). The phase of the cell division cycle is also known to influence antibiotic resistance within biofilms (127). Apart from heterogeneity observed within biofilms, population heterogeneity has been noted in *A. baumannii* which exists as opaque-virulent (VIR-O) and translucent-avirulent (AV-T) colonies. The AV-T cells produce denser biofilms than VIR-O cells, thereby showing an antibiotic resistance phenotype (128).

### Development of Persisters Within Biofilm: Temporary Resistance

The capacity of microorganisms to grow and survive at higher antibiotic concentrations than their MIC is called antibiotic tolerance. In contrast to resistance, tolerance is basically a transient phase and after exposure to antibiotics for a long time, antibiotics kill the bacteria. Bacterial populations in biofilms that exhibit increased antimicrobial tolerance are called “persister cells” (126). Persister cells are actually responsible for biofilm-associated infections (126, 129). The formation of biofilms and antibiotic-tolerant persisters contributes to the heterogeneity of *A. baumannii* populations, facilitating their adaptation to fluctuating environments. It was proposed that environmental stress (such as desiccation) causes the death of the main stressed population within the biofilm, where few viable surviving bacteria (persister cells) can resume growth and restore the original population, once the environmental conditions are suitable. This strategy is called the “bust-and-boom” strategy and *A. baumannii* follows this strategy (130, 131). Multiple mechanisms trigger antibiotic tolerance and development of persisters in biofilm such as stress triggered by antibiotics, the host immune responses, high osmolarity, ROS, changes in pH, efflux pumps, quorum signaling, oxidative stress responses, desiccation, or nutrient starvation. In addition, stringent response (SR), SOS response, and toxin-antitoxin (TA) modules can be activated during persister formation. These pathways result in decreased metabolism, protein aggregation, depletion of ATP, and inhibition of translation (132, 133). In the stringent response, the production of (p)ppGpp (also known as alarmone) is activated by the (p)ppGpp synthetases such as RelA and SpoT. (p)ppGpp regulates various transcriptional and metabolic pathways, such as phosphate, lipid, and amino-acid metabolism (134). Ultimately, the SR shuts down almost all metabolic processes leading to increase tolerance to antibiotics. The formation of *A. baumannii* persister cells which showed tolerance to rifampicin and colistin due to the deficiency of ppGpp has also been reported (135). DNA damage leading to activation of the SOS response which is also associated with antibiotic tolerance has been noted (136). Together, SR and SOS response molecules activate the TA system in which one gene encodes toxin that shows activities against DNA, RNA, membrane, cell wall synthesis, ATP, and the other encodes antitoxin that binds and inhibits the toxin. Alkasir et al. (137) reported that up-regulation of two toxin-antitoxin systems HigB/HigA, and DUF1044/RelB resulted in high ceftazidime tolerance among *A. baumannii* persister cells.

### Change in Bacterial Morphology due to Environmental Stress: Cause of Resistance

Stress responses result in physiological changes that control the composition and arrangement of the cellular envelope. Such changes in cell morphology can protect cells from nutrition deficiency, cold shock, heat shock, pH change, and also increase their non-susceptibility to several antimicrobial agents (138). *A. baumannii* embedded within biofilms also face stress due to high osmolarity that causes induction of porins such as OMP33–36 and CarO, leading to carbapenem resistance (30).

## Is There an Association Between Biofilm Formation and Antimicrobial Resistance in *A. baumannii*?

The type of correlation that exists between antibiotic resistance phenotypes and biofilm formation among *A. baumannii* is controversial.

Several studies have shown that antibiotic-resistant *Acinetobacter spp*. form strong biofilms compared to susceptible bacteria, indicating a positive correlation between antibiotic resistance and biofilm formation (83, 92–94, 115, 139–143) (Table 2). In the above studies, a statistically significant difference was found between strains with the high biofilm-forming ability and those with low/no biofilm-forming ability with resistance to several categories of antimicrobial agents (β-lactam group, cephalosporin group, aminoglycosides, quinolones, tetracycline, oxytetracycline, aztreonam, etc.). A particular study revealed high biofilm-forming ability among MDR *Acinetobacter* strains isolated from ICU patients compared to *Acinetobacter* strains isolated from non-ICU patients which showed resistance to fewer antibiotics (144). Similarly, another study reported high biofilm production among *A. baumannii* isolated from burn units. These isolates showed high resistance to antibiotics including carbapenems and also showed co-production of AmpC and ESBLs (145). Bardbari et al. compared biofilm-production ability between clinical and environmental *A. baumannii*. Clinical strains showed strong biofilm production ability compared to environmental strains (58.7 vs. 31.2%). However, a significant correlation was observed between the MDR phenotype and biofilm formation ability in both groups (*P* = 0.008) (94). Few of the above-mentioned studies also showed a high prevalence of biofilm-related genes including *omp*A*, bfm*S*, bap, csuE, bla*_*PER*−1_, and *epsA* in MDR *A. baumannii* with a high biofilm-forming ability (83, 92, 93, 139–141).

**Table 2:** Association between biofilm formation and antimicrobial resistance in *Acinetobacter spp*.

**Sr. no**	**Study**	**Country**	**Strain (numbers)**	**Sources of isolation**	**% of biofilm formers**	**Observations**	**References**
**Positive correlation between biofilm formation and antibiotic resistance**							
1.	Rao et al., 2008	India	*A. baumannii* (50)	Endotracheal aspirates, cerebrospinal fluid, wound swabs, urine, blood	62%-high biofilm former	• Resistance to four antibiotics such as amikacin (82 vs. 17.6%, *P* < 0.001), cephotaxime (88 vs. 11%, *P* < 0.001), ciprofloxacin (70 vs. 29%, *P* = 0.005), and aztreonam (38 vs. 11%, *P* = 0.039) was comparatively higher among biofilm producers than non-biofilm producers. • *bla*_PER−1_-horbouring *A. baumannii* was able to form strong biofilm in comparison to the isolates that did not possess the gene.	(92)
2.	Lee et al., 2008	Korea	*A. baumannii* (23)	Blood, sputum, urine	100%- biofilms former	• Cell adhesiveness and biofilm formation were significantly higher in isolates carrying the *bla*_PER−1_ as compared with isolates without this gene (*P* < 0.005 and *P* < 0.001, respectively). • RT-PCR showed a positive correlation between the level of expression of the *bla*_PER−1_ and the level of biofilm formation (*P* < 0.0001).	(83)
3.	Pour et al., 2011	India	• *A. baumannii* (47); • *A. lowffii* (3)	Urine samples, urinary catheters	• 12%- strong biofilm former • 10%-low biofilms former	• High biofilm forming strains exhibited high resistance to 27 antibiotics from different groups including β-lactam group (83.3%), cephalosporin group (94.4%), aminoglycosides (97%), quinolones (75%), tetracycline (66.6%) and oxytetracycline, and imipenem (33.3%).	(115)
4.	Nahar et al., 2013	Bangladesh	• *A. baumannii* (32) from ICU patients • *A. baumannii* (20) from non-ICU patients	Tracheal aspirates, blood, central venous catheter, peripheral blood, urine, wound swab, pus, throat swab, endotracheal tubes, burn samples, ascitic fluid, sputum, aural swab, oral swab, cerebrospinal fluid, and catheter tip	• 87.5%- biofilm former from ICU patients • 55%- biofilm former from non-ICU patients	• Resistance to antibiotics such as gentamicin (100 vs. 88.9%), amikacin (85.7 vs. 55.6%), netilmicin (85.7 vs. 11.1%), ciprofloxacin (82.1 vs. 54.4%), imipenem (81.0 vs. 22.2%) and colistin (7.1 vs. 0%) was higher among biofilm forming *Acinetobacter spp*. isolated from ICU than non-ICU isolates.	(144)
5.	Emami and Eftekhar, 2015	Iran	• *A. baumannii* (30) from burn unit • *A. baumannii* (30) from non-burn unit	• The burn isolates were mostly from wounds, blood, urine. • Non-burn isolates were from sputum, wound specimens, catheters, blood, cerebral spinal fluid, trachea	• 55.5%- biofilm former in non-burn isolates • 40.5%- biofilm former in burn isolates	• Non-burn strains significantly produced more biofilm compared to the burn strains (*P* < 0.05). • Biofilm-producing non-burn isolates were significantly more resistant to amikacin, meropenem, and tobramycin compared to the biofilm negative strains within the same group (*P* < 0.05). • AmpC and ESBL was much higher among the non-burn isolates compared to the burn samples (33.0 vs. 3.3%, *P* < 0.05).	(145)
6.	Thummeepak et al., 2016	Thiland	*A. baumannii* (221)	Sputum, urine, pus, blood, pleural fluid, ascetic fluid, and wound	76.9%- biofilm former	• The association between biofilm forming ability and gentamicin resistance was found to be significant (*P* = 0.017). • Antibiotic-resistant isolates possessed *omp*A (84.4%), *bfm*S (84%), *bap* (48%), *bla_*PER*−1_* (30.2%) and *eps*A genes (30.2%). However, biofilm formation related genes *omp*A and *bap* were associated with multidrug-resistant *A. baumannii* strains.	(139)
7.	Bardbari et al., 2017	Iran	• *A. baumannii* (75) from clinical samples • *A. baumannii* (32) from environmental samples	• Sputum, bronchoalveolar lavage, endotracheal aspiratesventilators, sink • Area, floor, hand staff, trolleys and bedside table, pillow and linens, and other fomites	• 31.2%- strong biofilm forming clinical isolates • 58.7%- strong biofilm forming environmental isolates	• Clinical strains showed strong biofilm production ability compared to environmental strains (58.7 vs. 31.2%). • Significant correlation was observed between the frequency of multidrug-resistant isolates and biofilm formation ability in both clinical and environmental strains (*P* = 0.008). • The study revealed the presence of *bla*_OXA−51_, *bla*_OXA−23_, *bla*_OXA−24_, *bla*_OXA−58_, and *bla*_PER−1_ among biofilm forming *A. baumannii*.	(94)
8.	Khamari et al., 2019	India	*A. baumannii* (14)	Blood, pus, urine, pleural fluid, endotracheal tube	• 100%- biofilm former • 71.4%-strong biofilm former	• *bla*_TEM_, *bla*_OXA_, *bla*_NDM_, *bla*_VIM_, *bla*_SIM_, and *bla*_PER−1_; class 1 integron were detected among the isolates.	(93)
9.	Yang et al., 2019	Taiwan	*A. baumannii* (152)	No data available	• 45.4%- strong biofilm former • 32.5%- moderate biofilm former • 15.6%- weak biofilm former	• A positive correlation was observed between biofilm forming capacity and resistance to ticarcillin, amikacin, gentamicin, ceftazidime, piperacillin, imipenem, and sulfamethoxazole-trimethoprim antibiotics (*P* = 0.018, 0.004, 0.003, 0.003, 0.033, 0.017, 0.007, respectively). • The study also revealed that biofilms-related genes such as *bap*, *bla*_PER_, *omp*A, and *csu*E genes were found in 81, 39, 91, and 69% of the biofilm producers, respectively. The strains carrying these genes formed stronger biofilm than the isolates without these genes.	(140)
10.	Ranjbar et al., 2019	Iran	*A. baumannii* (161)	Burn wood infections	• 70.6%- strong biofilm former • 12.2%- moderate biofilm former • 17.2%- weak biofilm former	• A significant association was observed between biofilm-forming ability and XDR phenotype (*P* = 0.001). • Multiple genes (*bla*_OXA−23−like_/*bla*_OXA−40−like_/*bla*_OXA−51_, *bla*_PER−1_/*bla*_VEB−1_, *bla*_IMP_, and *bla*_VIM_ and *tetB*) were found to be responsible for detection of drug-resistance in burn patients.	(141)
11.	Celik et al., 2020	Turkey	*A. baumannii* (60)	Tracheal aspirates, blood, urine, wound, sputum, CSF, abscess, bronchoalveolarlavagefluid	90%- biofilm former	• In biofilm-positive strains, antibiotic resistance was significantly higher against ampicillin/sulbactam, cefoperazone-sulbactam, chloramphenicol, piperacillin/tazobactam, and ciprofloxacin (*P* = 0.008, 0.038, 0.017, 0.027, 0.005, respectively).	(142)
12.	Asaad et al., 2021	Egypt	*A. baumannii* (161)	Sputum, endotracheal aspirate, wound swab	• 20.2%- strong biofilm former • 34%- moderate biofilm former • 16%- weak biofilm former	• Biofilm-producing isolates showed statistically significant higher resistance rate to ceftazidime, ampicillin/sulbactam, piperacillin/tazobactam, piperacillin, gentamycin, trimethoprim/sulfamethoxazole, tigecycline, and imipenem (*P* = 0.041, < 0.001, 0.006, 0.034, 0.028, 0.002, 0.002, and 0.02, respectively). • Presence of *omp*A gene (*P* = 0.002), *bap* gene (*P* = 0.012), MDR (*P* = 0.017), and XDR (*P* = 0.002) was significantly associated with biofilm-producing capability of the isolates, compared to non-biofilm producing capabilities.	(143)
**Negative correlation between biofilm formation and antibiotic resistance**							
1.	Rodríguez-Ba no et al., 2008	Spain	*A. baumannii* (92)	No data available	63%- biofilm former	• In comparison to non-biofilm forming *A. baumannii*, biofilm forming isolates were less frequently resistant to ciprofloxacin and imipenem (47 vs. 25%, *P* = 0.04; and 94 vs. 66%, *P* = 0.004, respectively).	(146)
2.	Han et al., 2014	China	*A. baumannii* (70)	No data available	• 50%- strong biofilm former • 29%- moderate biofilm former • 21%- weak biofilm former	• Resistance to levofloxacin (85.71%, 45.00%, 38.24%, *P* = 0.010), cefepime (71.43%, 45.00%, 29.41%, *P* = 0.027), and gentamicin (78.57%, 55.00%, 38.24%, *P* = 0.037) significantly decreased when biofilm-forming ability was strong.	(147)
3.	Zhang et al., 2016	China	*A. baumannii* (120)	Sputum	• 27.3%- strong biofilm former • 54.5%- moderate biofilm former • 18.2%- weak biofilm former	• Isolates which produced strong biofilm exhibited low-level resistance to gentamicin, minocycline, and ceftazidime (*P* < 0.05).	(148)
4.	Qi et al., 2016	China	*A. baumannii* (268)	No data available	• 23%- strong biofilm former • 74.7%- weak biofilm former	• Among the strong biofilm-formers, 79.4% were non-MDR isolates and, 20.6% were MDR/XDR ones. • Among the weak biofilm-formers, 12.4% non-MDR and 87.6% MDR/XDR isolates. • Strains that were negative for biofilm formation consisted of 8.7% non-MDR and 91.3% MDR/XDR isolates.	(31)
5.	Krzysciak et al., 2017	Poland	*A. baumannii* (15)	Blood, central nervous system, pulmonary	80–90%- biofilm former	• Strains showing sensitivity to amikacin, tobramycin, trimethoprim/sulfamethoxazole and ciprofloxacin from ICU patients produced more biofilm than strains showing resistance to these antibiotics.	(149)
6.	Wang et al., 2018	Taiwan	*A. baumannii* (269)	Blood	26%- biofilm former	• MDR isolates was significantly lower (*P* = 0.006) in the biofilm-forming group. • Biofilm-forming isolates were significantly more susceptible to most commonly used antibiotics including amikacin, gentamicin, ceftazidime, cefepime, ciprofloxacin, imipenem, and meropenem (*P* = 0.040,0.043, 0.003, 0.009, 0.001, 0.035, 0.018, respectively).	(150)
7.	Shenkutie et al., 2020	China	*A. baumannii* (104)	Sputum, blood, urine, soft tissue, hospital environments	• 25%- strong biofilm former • 14.4%- moderate biofilm former • 20.2%- weak biofilm former	• Non-MDR strains (66.1%) showed strong biofilm formation.	(151)

*Studies have been arranged in chronological order*.

The diversity and abundance of antibiotic resistance genes (ARGs) in biofilms of *Acinetobacter spp*. have been investigated by several authors to highlight the fact that the probability of accumulation of ARGs (*bla*_OXA−51_, _OXA−23_, _OXA−58_, _OXA−72, OXA−24/40_-like genes, *bla*_TEM_, *bla*_VIM_, *bla*_NDM_, *bla*_SIM_, *ompA, xerC*, and *gyrA*) or IS elements (IS*Aba1*, IS*Aba3*) in biofilms is higher rather than in the planktonic cells (93, 94, 141).

Though most studies have shown a positive association between biofilm formation and antibiotic resistance in *Acinetobacter spp*., some studies have documented an inverse relationship between the biofilm formation capacity of clinical strains of *A. baumannii* and MDR/XDR phenotype (146–152). These studies are comparatively fewer than the studies that show a positive correlation. These studies have been described in detail in Table 2.

## HGT Within Biofilms of *Acinetobacter spp*.: Exchange Matters

Antibiotic resistance genes (ARGs) are often encoded in mobile genetic elements (MGEs) such as conjugative and non-conjugative plasmids, integrative and conjugative elements, transposons, and bacteriophages (153–156). Plasmids and MGEs can easily be transferred to closely related or distantly related bacteria *via* HGT. HGT also occurs in biofilms and certain factors that enhance the HGT within biofilms are the EPS matrices of biofilm that limit bacterial motility, increased cell-to-cell contact, quorum sensing, high cell density that helps in bacterial interactions. Apart from these, the presence of eDNA, oxygen availability, the SOS response, extracytoplasmic stress, and biomass surface increase the efficiency of plasmid transfer (157).

During conjugation, transfer of plasmid occurs through conjugation pilus which is proteinaceous in nature and serves as a link between donor and recipient cells (155). Transfer of ARGs within a biofilm is mainly associated with conjugation because biofilm cells are attached to a matrix, located close together that provides direct cell to cell contact. Cells remain metabolically active and are embedded within the EPS matrix in which the cells are protected against the harsh environment. These factors enhance HGT within biofilms *via* conjugation and thereby are considered as HGT hotspots (158–161). Apart from conjugation, transformation is another important mechanism that transfers naked genes within biofilms (162). Free DNA which is released by cell lysis can serve as the donor for transformation.

All AMR genes can be transferred *via* HGT within the biofilm. However, there are limited studies that show the transmission of specific genes/resistance plasmids within *A. baumannii* biofilm *via* conjugation or transformation. Willium et al. provided the first evidence of the natural transformation of *Acinetobacter* BD413 cells in river biofilms with a mercury resistance plasmid pQM17 (163). Hendrics et al. showed effective natural transformation in biofilms of *Acinetobacter* spp. strain BD413. The nature of transformants changed with the change in the amount of exogenous DNA. When the amount of DNA was low, transformants formed at the biofilm attachment surface while with an increasing amount of DNA, the accumulation of transformants was observed at the bottom of the biofilm (23). Another study showed both conjugal transfer and natural transformation of plasmids from *A. baumannii* to *E. coli* HB 101 and *A. baylyi*, respectively (115). The spread of a highly promiscuous carbapenem hydrolyzing gene *bla*_NDM−1_ was noted from *E. coli* J53-*bla*_NDM−1_ transconjugant to *A. baumannii* biofilms *via* conjugation (24).

Apart from conjugation and transformation, the transfer of plasmid DNA is also mediated *via* the formation of nanotubes (elongated extracellular structure employed cell to cell contact and composed of proteins), outer membrane vesicles, and bacteriophages in *Acinetobacter spp*. (25–29) but their role in the transfer of ARGs among biofilm-embedded *Acinetobacter* has not been elucidated yet. HGT also occurs in the microbial ecosystem in the human intestinal tract. HGT in the human gut microbiome can occur *via* different mechanisms like transduction or conjugation. Different bioinformatics tools and experimental approaches have been developed to determine the association and transfer of antibiotic resistance genes in the gut microbiome (164). The HGT in the gut microbiome may lead to the development and spread of antibiotic resistance genes among commensals and opportunistic pathogens (165).

## Resistance of *A. baumannii* Biofilm-Associated Cells Toward Disinfectants

Disinfectants are chemical agents used to play a key role in the prevention of nosocomial transmission of infectious pathogens (166). The commonly used disinfectants against infectious pathogens in the hospital or industrial environments are 70% ethanol, chlorhexidine, sodium hypochlorite, quaternary ammonium compounds, benzalkonium chloride (BZK), benzethonium chloride (BZT), phenolic disinfectants, hydrogen peroxide, and silver ions (167). Several factors lead to the enhanced resistance toward disinfectants in *A. baumannii*, e.g., reduced diffusion or reaction limitations of disinfectants in biofilms, overexpression of the EPS matrix, biofilm-specific efflux pumps, phenotypic adaptations of biofilm cells to sublethal concentrations of disinfectants, alterations in genotypic features like gene transfers and mutations due to stress responses, and specific microenvironment conditions that inactivate biocides (168–170). A study has reported that resistance to antiseptics and disinfectants in *A. baumannii* is largely mediated by efflux proteins encoded by *qac* genes. The *qacA/B* genes encode proteins of the MFS-family whereas *qacC, qacE, and qacF* encode efflux proteins of the SMR-family which are located on mobile elements, thus facilitating their spread and resistance to disinfectants (171). Indeed, inappropriate use of disinfectant solutions with an adequate concentration leads to the selection and emergence of microorganisms resistant to disinfectant in the hospital setting (172).

## *A. baumannii* Sneaks Into Hospitals: Adult and Paediatric Patients

Modern medicine and improved healthcare systems save numerous lives. However, these medical interventions have also provided conditions for microbial growth, entry into human bodies, and infections. Several indwelling devices in the hospital create breaches in the body's defense mechanisms allowing easy access for microorganisms to enter the body. The patients in ICUs already have underlying conditions and are vulnerable to infections; indwelling devices offer more opportunities for such infections. *A. baumannii* can survive for long periods in the hospital environment, particularly on inanimate surfaces, which may act as a reservoir for cross-colonization and infection outbreaks. Moreover, a previous study showed that *A. baumannii* can retain its virulence under stress (desiccation and/or starvation in hospital settings) which could facilitate infections (22). In healthcare setups, biofilms pose a serious problem due to the increased antimicrobial tolerance and the potential of biofilm-associated organisms to cause infections in patients with indwelling medical devices (173). *A. baumannii* easily acquires resistance and the biofilm formation rate in *A. baumannii* is higher than other species, making this organism a major cause of concern in ICUs. In most cases, indwelling medical device-related infections including bloodstream infections and urinary tract infections are biofilm-associated infections (173). Central venous catheters (CVCs) are the most common medical devices, followed by endotracheal tubes (ETT), ventilators, medical implants that pose a risk of device-related infection. Several *in vitro* and *in vivo* studies reported biofilm-formation of *A. baumannii* on several abiotic surfaces including hospital equipment and indwelling medical devices, such as catheters, endotracheal tubes etc (174–176). Carbapenem-resistant *A. baumannii* were also found to form biofilm on extracorporeal membrane oxygenation catheters (177). Several reports have also indicated the presence of *A. baumannii* biofilm on different hospital material, such as latex, anodized aluminum, stainless steel, and polycarbonate surfaces (86, 110, 174). Development of biofilm on medical devices depends on several factors including adherence of microorganisms for prolonged periods of time that results in irreversible attachment of organisms, physicochemical characteristics of the surface, cell density and types of the adherent cells, nutrient composition of the medium, flow rate of liquid through the device, drug concentration, ambient temperature and most importantly hydrophilicity and surface charge of the material of the medical devices (178). The surface proteins of microorganisms that act as virulence factors can specifically bind to host extracellular matrix proteins, such as fibrinogen, fibronectin, and collagen by van der Waals forces and H-bonds (179). These proteins have a high affinity for implants and become easily attached to the implant surface and develop as microcolonies over the entire surface of the host (179). The infections associated with colonization of *A. baumannii* on medical devices are discussed below. Each important device-related infection is dealt with separately and both adult and pediatric studies have been categorized (178).

### Ventilator-Associated Pneumonia (VAP)

Ventilators are devices that support breathing in seriously ill patients by forcing oxygen into the lungs. Some ventilators have tubes that are inserted into the bronchus bypassing an important defense of the lungs, the ciliated cells, introducing microbes easily into the lungs. This entry is facilitated in organisms that form biofilms. Biofilm formation in the oropharynx, tracheostomy, and endotracheal tubes of ventilated patients has been suggested to play a role in the development of ventilator-associated pneumonia (VAP) (180). Sometimes, microorganisms may directly reach the lower airways by inhalation as a result of contamination of medical equipment, and they may reach the lungs. These tubes are indwelling prostheses and are typically made from polyvinyl chloride, latex rubber, or silicone materials, providing a potential surface for the growth of bacteria, especially *A. baumannii*. A very recent study detected *A. baumnnii* as one of the most common organisms capable of forming a biofilm on tracheostomy tubes among critically ill patients (181). A large surveillance study from the United States showed the association of *A. baumannii* with ~ 5 and 10% of ICU-acquired pneumonia (182). Malacarne et al. (183) reported 28.6% of the cases of late-onset VAP due to *A. baumannii* which were preceded by tracheobronchial colonization with *A. baumannii*. The formation of biofilm on ETT and its association with VAP was analyzed in several studies where the most frequent bacteria were *A. baumannii* which lead to high mortality of patients (175, 184–189).

Ventilator-associated pneumonia (VAP) is the second most frequent cause of nosocomial infection in children in ICUs in developing countries (190). Children who develop VAP also have an increased risk of mortality and morbidity (191, 192). *A. baumannii* is increasingly recognized as an important pathogen causing VAP in neonatal and children ICUs with a trend of high resistance to broad-spectrum antibiotics including carbapenems and colistin (176, 193–196). These studies clearly indicate the association of *A. baumannii* and VAP.

### Bloodstream Infections (BSIs)

Bloodstream infections (BSIs) are primarily associated with the presence of the CVC or as a consequence of extensive hospital-acquired pneumonia (197). Catheters introduce microorganisms into the body as they are directly inserted in the vein to inject antibiotics or other medicines in hospitalized patients. The CVC-related infections are dependent on the patients' age and insertion procedure of CVC. The degree of severity of catheter-related bloodstream infections (CRBSI) is increased when microorganisms form biofilms. The best way to avoid CRBSI is to reduce the unnecessary catheterization, reduce the indwelling duration of CVC, use antibiotic-impregnated (like minocycline/rifampin) catheters, and use preventive locks (197). CRBSI among ICU patients due to *A. baumannii* biofilm formation on CVC has been noted (198, 199). Recent studies also showed BSI with highly virulent *A. baumannii* ST2 and ST191 belonging to International Clonal Lineage II that showed strong biofilm-forming ability (200, 201).

CVCs are also used in modern pediatric medication for various purposes including hemodynamic monitoring, infusion of vasoactive medication, hemodialysis, long-term use for chemotherapy, antibiotic treatment, or immunological diseases. CRBSI and central line-associated bloodstream infection (CLABSI) are also reported among children (202–204).

### Urinary Tract Infection (UTI)

Among adults, urinary tract infection (UTI) is mostly associated with urinary catheters. Catheter-associated UTIs (CAUTIs) represent about 40% of all nosocomial infections as most of the hospitalized patients need an indwelling urinary catheter throughout their hospital stay (205). The placement of the catheter leaves the sphincter open allowing unbridled access to pathogens present in the hospital environment, the washing action of the urine is also absent in catheter-fitted patients. The catheter also presents a perfect surface for micro-organisms to adhere to and start biofilm formation (206). Given these multiple factors, UTIs are common. Biofilm formation depends on the duration of catheterization as 10–50% of short-term catheterized patients (≤7 days) experience biofilm formation meanwhile biofilm is formed inevitably in all long-term catheterized patients (>28 days) (173, 178). *A. baumannii*-associated UTI causes serious medical problems because of treatment difficulties due to their resistance to carbapenems and third-generation cephalosporins. Various studies showed *Acinetobacter* as one of the biofilm-producing organisms associated with CAUTI (5, 207–212).

Urinary tract infections (UTIs) are common infections among children in the first 2 years of life and are considered a common disease in school and pre-school children. Most *A. baumannii* strains are capable of producing biofilm in percutaneous nephrostomy tubes or urinary catheters, therefore *A. baumannii* may contribute significantly to UTIs in hospital admitted children and also cause community-acquired UTIs (213, 214).

### Traumatic Battlefield, Wound, Burn, Skin, and Soft Tissue Infections

Biofilm formation on medical devices draw significant attention in healthcare settings, but the biofilm formation ability of *A. baumannii* on biotic surfaces such as on wound, burn, skin and soft tissue have also been noted. Such tissue-related infections in immunocompromised, cancer, and diabetic patients have raised serious concerns (215). Bacterial colonization in open wounds damages the healing process. It has been reported that biofilms are causative factors for many chronic non-healing ulcers (216). Nosocomial *A. baumannii* deep wound and burn wound infections have been reported in natural or man-made disasters (earthquakes, bombing, military operations) (217–219). Evidence of *A. baumannii* biofilm in wounds has also been provided by several other studies (220–224). Skin and Soft Tissue Infections (SSTIs) are often accompanied by *A. baumannii* bacteremia (225–228). The spectrum of infection can extend from cellulitis to necrotizing fasciitis.

### Orthopedic Implant-Related Infections

Orthopedic devices are commonly used worldwide for a wide number of procedures including hip or knee replacements, fracture treatment, joint, ligament, and tendon replacements, and other surgical processes. These procedures have become extremely common to restore the function of affected joints, bones, or limbs. Implant-associated infections remain a major problem in orthopedic procedures and is caused by surface-adhering bacteria that form biofilm. The reported rate of implant-related infection is about 5% (229). Implant-associated infection can either be early (within the first 2 months of surgery) or delayed (between the third and the 24th month). Among different microorganisms that cause implant-associated infections, *A. baumannii* has been diagnosed in the case of periprosthetic joint infections (230, 231).

### Neonatal Intensive Care Units

*Acinetobacter baumannii* has been a major cause of neonatal sepsis and several studies have reported outbreaks in neonatal units (232–234). Neonatal sepsis with drug-resistant and even carbapenem-resistant *A. baumanii* has also been reported (235, 236). Sepsis in neonates can also lead to meningitis causing high mortality rates. Several neonatal meningitis cases have also been reported due to MDR *A. baumannii* (237–241).

As with adult ICUs, neonatal ICUs also provide the same set of conditions and devices for *A. baumannii* to flourish. Most neonates in the ICUs are premature or of low birth weight and require prolonged hospitalization. As they are vulnerable and already fighting for life, they also require life-support systems such as ventilators. Prolonged stay at the hospital on life-support systems always increases the chances of infection particularly in neonates who are on antibiotics and have an immature immune system (242). Apart from infection, colonization of the gut with *A. baumannii* has also been reported in hospitalized neonates, increasing the possibility of subsequent sepsis due to translocation of the gut bacteria (243). A comparison of bacterial etiology of neonatal sepsis reveals that higher the level of care (Level III against Level II/I) greater the rate of *A. baumannii* sepsis. A recent study noted *A. baumannii* as the predominant cause of neonatal sepsis; this study was carried out in 3 tertiary care hospitals in New Delhi, all with level III care (244). Level III units are equipped with devices, which facilitate the formation of biofilms clearly indicating the link. Sources of infection could be varied and unexpected, the devices, the hospital staff, or even mothers who handle the neonate for breastfeeding or kangaroo care (193, 194, 245). Vigilance and infection control are of utmost importance particularly in units that care for neonates.

## Strategies of Preventing *A. baumannii* Biofilms: Fighting Back

**Figure 5:**
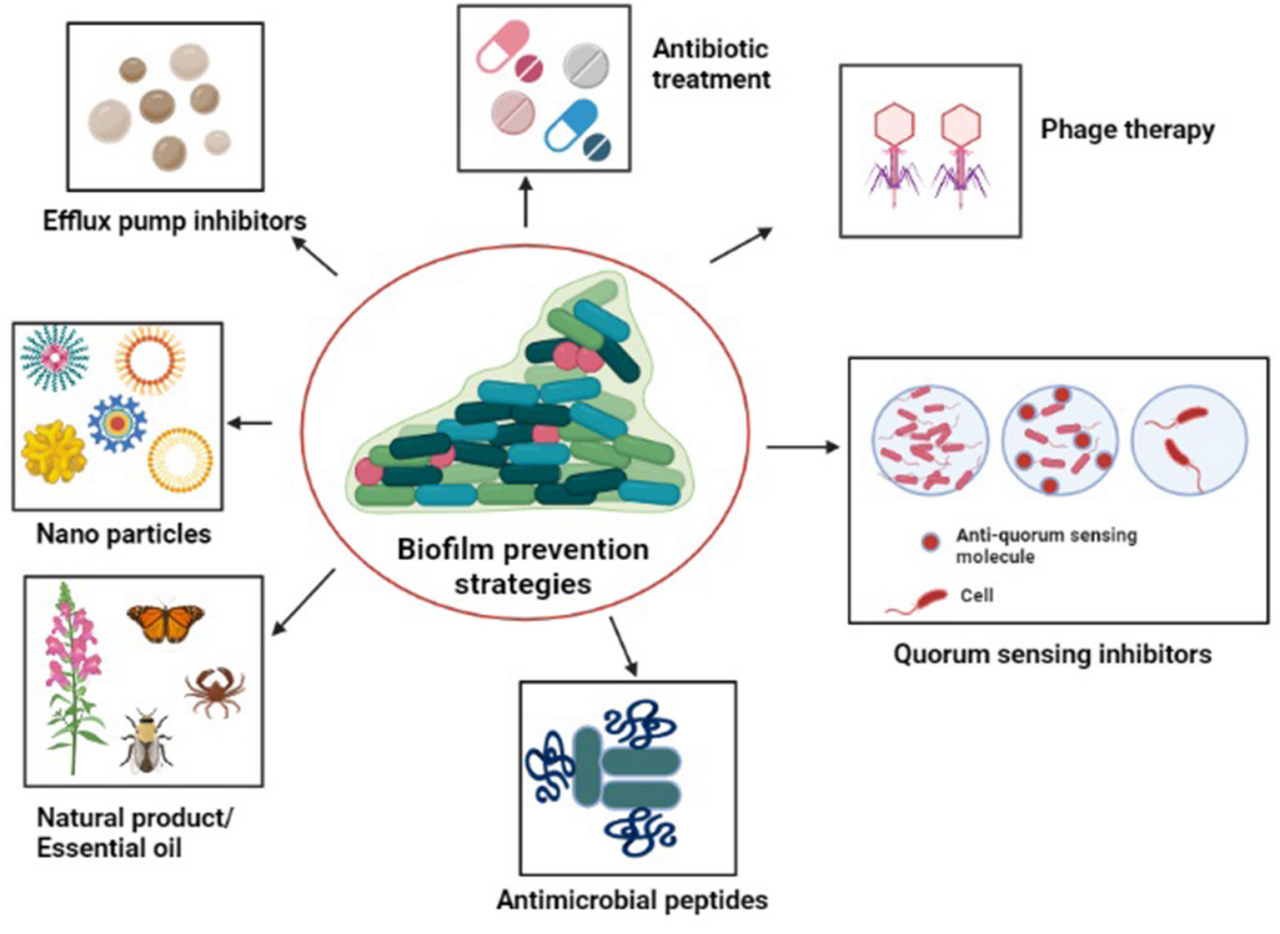
Diagrammatic representation of the strategies to tackle antibiotic-resistant biofilm communities: antibiotic treatment, quorum sensing inhibitors, natural products/essential oils, antimicrobials peptides, efflux pump inhibitors, nanoparticles, and phage therapy.

With the aim to limit *Acinetobacter* adhesion to biotic or abiotic surfaces and to inhibit biofilm growth, numerous effective novel anti-biofilm remedies have been developed, few of which have been discussed below (Figure 5).

### Antibiotic Treatment: Active Combinations

Due to the high antibiotic tolerance of biofilm cells and restricted penetration of antibiotics through the matrix, it is difficult to treat biofilm-associated infections. Few antibiotics including quinolones, rifampicin, tetracycline, and macrolides show higher penetration ability. Monotherapy is generally inappropriate because of the high antibiotic tolerance of biofilm-embedded cells. With no new antibiotics in the pipeline, antibiotic combinations are the most logical option for the treatment of biofilm infections (246). Combination therapies such as imipenem-rifampicin, colistin-rifampicin, imipenem-colistin-rifampicin, meropenem-sulbactam, and tigecycline-sulbactam have shown significant inhibition of *A. baumannii* biofilms (247–249). Rifampicin generates hydroxyl radicals, which are highly reactive oxygen forms and the major components of bactericidal agents. A particular study investigated the biofilm inhibitory effect of five biofilm inhibitors (zinc lactate, stannous fluoride, furanone, azithromycin, and rifampicin) either alone or in combination with one of the four antibiotics (imipenem, meropenem, tigecycline, and polymyxin B). These biofilm inhibitors in combination with antibiotics showed different degrees of *in vitro* synergistic and additive antibacterial effects against XDR *A. baumannii* under sub-minimal inhibitory concentrations (250). Different studies showed that antimicrobial combinations such as colistin-levofloxacin, colistin-tigecycline- and tigecycline-levofloxacin or these combinations with clarithromycin were used as catheter lock solutions, therefore, effective in the treatment of *A. baumannii* catheter-related infections (251). Use of minocycline-rifampicin impregnated bladder catheters, gentamicin-releasing urethral catheters, and norfloxacin along with blends of a copolymer of ethylene-vinyl acetate and polyethylene oxide as catheter coatings showed a reduction of CAUTIs (252–254). Clinical trials also showed that the use of medicated CVCs that contain antimicrobial solutions (minocycline-rifampicin, chlorhexidine/silver sulphadiazine, rifampicin-miconazole) on both the external and the luminal surfaces of catheters, exhibited significant inhibition of bacterial attachment but only the minocycline-rifampicin impregnated CVCs were able to reduce CRBSIs (255–257). TRL1068 (a human monoclonal antibody active against an epitope of DNABII proteins that stabilizes biofilm eDNA in both GPB and GNB) in combination with imipenem showed a significant reduction of *A. baumannii* adherence to catheters (258).

### Quorum Sensing Quenchers: Stopping the Communication

Since QS contributes to biofilm production, inhibition of QS signaling pathways result in a reduction of biofilm formation and can be used as a novel therapeutic strategy (259). Biofilm formation in *A. baumannii* is dependent on the activation of a LuxI/LuxR-type QS network involving AbaI synthase, AbaR receptor, and various AHLs. QS inhibition could be achieved by targeting the synthesis of AHLs. For example, mutation of the AHL synthase AbaI affects the surface-associated motility and robust biofilm formation in *A. baumannii* ATCC®17978 (104). A low concentration of triclosan (chemical with antibacterial properties) could inhibit the enoyl-acyl carrier protein reductase (FabI), which is important for AHL acyl chain formation (260). The use of natural compounds such as allin, patulin/clavacin, and vanillin were found to interfere with AHL receptors, leading to the prevention of QS signals (261–263). Moreover, AHL analogs, AbaR antagonists (such as streptomycin), anoR antagonist (virstain), and antagonists for di-guanylate cyclase enzyme that synthesize cyclic di-GMP were found to inhibit QS and subsequently biofilm formation in *A. baumannii* and *A. noscomialis* (264–268). A marine steroid (Siphonocholin) which has anti-QS activity was found to inhibit the EPS production, swarming motility, and biofilm formation in *A. baumannii* (269). Certain genetically engineered quorum quenching enzymes such as AHL lactonase and MomL could also successfully inhibit QS signal, leading to disruption of biofilm structure (270–272).

### Natural Products/Essential Oils: Nature to the Rescue

Natural products such as microbial, plant, and animal derivatives can effectively reduce *A. baumannii* infections. Metabolites produced by bacteria have been proved to be effective against *A. baumannii* biofilm (273). A study showed that *A. calcoaceticus* could be strongly inhibited by other bacterial (*Burkholderia cepacia, Methylo bacterium spp., Mycobacterium mucogenicum, Sphingomonas capsulata*, and *Staphylococcus spp*.) crude cell-free supernatants (274). Maipomycin A which is isolated from the metabolites of the marine actinomycete and acts as an iron chelator inhibits *A. baumannii* biofilm formation on medical materials including silicone catheters and endotracheal tubes (polyvinyl chloride) (275). Several sets of compounds derived from marine sponges showed inhibition of different bacterial biofilm including *A. baumannii* specifically through non-microbicidal mechanisms (276). 5-episinuleptolide isolated from *Sinularia leptoclados* (a genus of soft coral in the family Alcyoniidae), showed anti-biofilm activity against *A. baumannii* ATCC®19606TM and MDR *A. baumannii* strains by decreasing the expression of the *pgaABCD* locus, which encodes the extracellular polysaccharide (poly-PNAG) of the biofilm structure (277). A secreted serine protease, termed “PKF,” was identified in *A. baumannii* that suppressed *A. baumannii* biofilm formation (278). Myrtenol which is a bicyclic monoterpene isolated from various plants showed strong anti-biofilm properties against clinical strains of *A. baumannii* at a concentration of 200 μg/ml. Myrtenol specifically inhibited the mature biofilm, reduced the biofilm thickness, inhibited the biofilm-associated virulence factors, and improved the susceptibility of strains toward conventional antibiotics. Upon myrtenol treatment, suppression of the biofilm-associated genes such as *bfmR, csuA/B, bap, ompA, pgaA, pgaC*, and *katE* was found (279). Natural products such as essential oils (EO), plant secondary metabolites could exert broad-spectrum antibacterial activity by disrupting bacterial membrane integrity and also by inhibiting ATP synthesis that result in leakage of metabolites/ions (280). Different EOs with MBIC between 0.3–1.25 μl/ml derived from flowery plants such as *Mentha pulegieum* L. and *Ziziphora tenuior* L., could damage the *A. baumannii* biofilm structure to a great extent. D-isomenthone, pulegone, isopulegone, menthol, and piperitenone were found to be the major components of the plant extract (281, 282). Anti-biofilm properties of four essential oil components (carvacrol, eugenol, thymol, and vanillin) were assessed against the organisms adhering to food surfaces in the meat industry. Carvacrol and thymol showed the most remarkable antimicrobial effect against *A. baumannii* strains (283). Similarly, other studies also showed that different EOs derived from plants and rich with these essential oil components (Oregano oil rich in carvacrol and thymol contents; cinnamon oil rich in eugenol; Eucalyptus camaldulens oil rich in thymol content) were active against *A. baumannii* wound infections (284, 285). EO-based nanoemulsions, prepared from *Thymus daenensis* (plant), showed potent antibiofilm activity against *A. baumannii* on sublethal dose (56.43% inhibition in 1/2 MIC concentration) after 24 h of incubation (286). Shivaprasad et al. (287) reported the activity of different antibiotics such as imipenem, cefipime, cotrimoxazole, amikacin, ciprofloxacin, piperacillin/tazobactum, cefoperazone, and gentamicin was enhanced against MDR/XDR *A. baumannii* complex when used in combination with lemongrass EO which showed 65–79% of anti-biofilm activity at a dilution between 0.625–0.156 μl/ml. Most of the essential oils have been tested for *in vitro* activity against bacteria, only few were subsequently tested in cellular or animal models. Ismail et al. (288) reported that anti-biofilm activity of *Pimenta dioica* leaf EOs (85% inhibit) was higher than *Pimenta racemosa* leaf EOs (34%) against *A. baumannii* and both *P. diocia* and *P. racemosa* leaf EOs showed a bactericidal action against *A. baumannii* within 6 h at 2.08 μg·ml^−1^. In addition, a significant reduction of *A. baumannii* microbial load in the mice wound infection model was also found (288). Similarly, oregano oil showed significant antibacterial activity against 11 MDR clinical isolates including *A. baumannii* with a MIC ranging from 0.08 mg/ml to 0.64 mg/ml. An *in vivo* study demonstrated that oregano oil topically applied 24 h after bacterial inoculation, sufficiently reduced the bacterial load in the wounds by 3 log_10_ in 1 h (289).

### Antimicrobials Peptides: An Alternative to Antibiotics

Antimicrobial peptides (AMPs) are cationic peptides (15–30 amino acids long) produced by the innate immune response and target the negatively charged cell membrane of bacteria (290). Different AMPs of biological origins have been reported to inhibit *A. baumannii* biofilm. Human AMP LL37 is one such AMP that inhibits *A. baumannii* biofilm (291). Human milk lactoferrin which is an iron-chelating AMP also showed slightly more potent antibacterial activity than bovine milk lactoferrin against *A. baumannii* biofilm (292). Derivative (D-RR4) of a small synthetic peptide, RR (12 amino acids) exhibited potent antibacterial and anti-biofilm activity against *P. aeruginosa* and *A. baumannii* in macrophage cells as well as in a *Caenorhabditis elegans* model (293). Magainin 2 (an antimicrobial peptide consisting of 23 amino acids isolated from the skin of the African clawed frog *Xenopus laevis*), showed the ability to inhibit *A. baumannii* biofilm at a very low concentration (294). Potent activity against *A. baumannii* biofilm has also been observed with several AMPs derived from flies, such as cecropin AMP identified from *Musca domestica* and another complex of AMPs (Fly larvae immune peptides) from *Calliphora vicina* (295, 296). Jakiewicz et al. (297) investigated the antimicrobial activity of eight peptides (aurein 1.2, CAMEL, citropin 1.1., LL-37, omiganan, r-omiganan, pexiganan, and temporin A) of different biological origins against *A. baumannii* biofilm on tracheal tube fragments. Among these peptides, strong anti-biofilm activity was shown by CAMEL and pexiganan with MIC values of 2 μg/ml (297). More recently, four synthetically produced chimeric AMPs have been proven to show anti-biofilm activities against MDR *A. baumannii*. These AMPs worked synergistically with ciprofloxacin, cefotaxime, or erythromycin to inhibit *A. baumannii* biofilm (298). The activity of the cationic antimicrobial peptide WLBU2 (24-residue engineered cationic amphipathic peptide) showed promising activity in combination with imipenem and tobramycin against planktonic cells and biofilm of MDR *A. baumannii* (299). A synthetically produced cyclic peptide ZY4 (17 amino acid long) exhibited biofilm eradication activity by killing the persister cells of *A. baumannii* and *P. aeruginosa* within the biofilm. Moreover, the therapeutic potential of ZY4 was also determined in an *A. baumannii*-induced bacteremia mice model (300). Some AMPs are used as ointments for medical device surfaces or for the reduction of superficial tissue infections (301). In an *in vitro* experiment, Temporin-L showed an anti-biofilm effect without cell damage, representing the great potential for clinical application (302). AMPs have great potential in clinical treatment by effective removal of biofilms.

### Efflux Pump Inhibitors: Blocking an Important Machinery

Studies had shown that efflux pumps play various roles in biofilm formation in ESKAPE pathogens; hence, inhibiting their function by efflux pump inhibitors (EPI) could also inhibit biofilm formation. A compound can be considered as a potent EPI when it has a broad substrate specificity and low off-target toxicity (303). One of the most common EPIs that is usually used in *A. baumannii* is phenylalanine-arginine β-naphthylamide (PAβN) which was reported to inhibit *A. baumannii* biofilm formation (304). Two novel serum-associated EPIs known as ABEPI1 and ABEPI2 were identified that potentiated the activities of antibiotics against *A. baumannii* grown in human serum. Both compounds exhibited similar antibiotic potentiation profiles toward minocycline and ciprofloxacin and inhibited pump activity (305). Another study also generated a set of compounds (pharmacophores) consisting of 2-substituted benzothiazoles which showed significant inhibition of AdeABC efflux pumps in combination with ciprofloxacin (306). Krishnamoorthy et al. assessed the efficacy of two microbicides such as cetrimide and chlorhexidine which adversely modified the expression and function of AdeABC efflux pump in biofilm-associated *A. baumannii*. Furthermore, they established that these microbicides decreased the negative charges on *A. baumannii* cell membranes, causing dysregulation of the efflux pump, leading to cell death (307).

### Nanoparticles: Small Is Beautiful

Nanoparticles (NPs) are very small in size (<100 nm) with a large surface area and extremely reactive nature. They show broad-spectrum activities against both GPB and GNB and sometimes they have been preferred over antibacterial agents. NP can disrupt biofilm integrity by penetrating bacterial cell membrane, generating ROS, causing ATP depletion, and interacting with polysaccharides, eDNA, proteins, and lipids (308). Different studies have been carried out to understand the role of NPs to inhibit *A. baumannii* biofilm. A study showed the use of nitric oxide (NO) releasing NPs to treat *A. baumannii* biofilm-related wound infections *in vivo* in murine models (309). Another study showed the disruption of *A. baumannii* biofilm on exposure to nanoemulsion of cetylpyridinium chloride, a quaternary ammonium salt (310). NPs coupled with metals or natural product extracts have been shown to possess inhibitory activity against both planktonic and biofilm-associated cells. A particular study showed the action of silver NPs, gold NPs and silver-gold bimetallic NPs against *A. baumannii* biofilms. These NPs showed 88% of *A. baumannii* biofilm inhibition (311). Several other studies also showed the efficiency of silver NPs (AgNPs) in inhibition of *Acinetobacter* biofilms as these NPs easily penetrate the thick EPS in biofilms. Positively charged AgNPs interact with negatively charged eDNA that plays a major role in the inhibition of biofilms (312, 313). In addition, *A. baumannii* biofilm inhibition was also observed by selinium NPs, curcumin NPs, aluminum oxide NPs, etc. (314–316). NPs in combination with antibiotics have also been reported to possess substantial antibiofilm activity. AgNPs were found to act synergistically with imipenem, as imipenem lyses the cell wall of bacteria leading to increased penetration of AgNPs into the cells (317). NPs can also be used as drug delivery carriers or as catalysts to promote the penetration of drugs into biofilms; improve the solubility, stability, and biocompatibility of drugs (318).

### Phage Therapy: Cocktails That Work

The use of bacteriophages is another approach to control and remove biofilms. Different lytic bacteriophages such as AB7-IBB2 (family of Podoviridae), AB7-IBB1 (Siphoviridae), and vB_AbaMIME-AB2 were found to inhibit *A. baumannii* biofilms (10^8^ CFU/well) on abiotic and/or biotic surfaces (60–>80%) (319–321). Lood et al. identified 21 distinct lysins (prophages) induced from 13 diverse *A. baumannii*. Among these lysins, PlyF307 showed the greatest activity, and treatment with PlyF307 was able to significantly reduce planktonic and *A. baumannii* biofilm both *in vitro* and *in vivo* (322). Thandar et al. (323) showed that the C-terminal amino acids (15, 108–115, 117–138) of a phage lysin named P307, alone could efficiently kill *A. baumannii* (>3 logs) while its engineered derivative (P307SQ-8C) showed improved activity (>5-log kill) along with polymyxin B. Two different phages (B_AbaM_ISTD, and vB_AbaM_NOVI), isolated from Belgrade wastewaters, were found to inhibit *A. baumannii* biofilms (324). *A. baumannii* biofilm biomass was inhibited when an environmental phage cocktail was used in combination with antibiotics such as ciprofloxacin, trimethoprim/sulfamethoxazole, gentamicin, tobramycin, imipenem, and meropenem which are generally used in the treatment of UTI. While phage cocktail combined with levofloxacin and amikacin, did not act synergistically (325). An excellent method of inhibiting *A. baumannii* biofilm was devised by Ran et al. (326) by combining photodynamic bacteriophages (ABP) and Nile blue photosensitizers (NB). NB photosensitizer possessing sulfur atom displayed ROS production ability. Both *in vitro* and *in vivo* experiments proved that NB-phage bioconjugate (APNB) could bind to the main components of biofilms and reduce drug resistance caused by biofilms (326).

### Other Biofilm Inhibitors

Some other chemicals or compounds that do not belong specifically to the above-mentioned groups have also been reported to inhibit *A. baumannii* biofilms. In order to determine the effectiveness of biocides for the eradication of MDR *A. baumanni* biofilms, hydrogen peroxide, and hydrogen peroxide-based formulations were tested. Mixed-culture biofilm cells were found to be more resistant to some biocides, such as hydrogen peroxide and sulfathiazole, than the single-species *Acinetobacter* biofilm cells. Higher potential for biofilm removal and killing was found among oxidizing biocides, such as sodium hypochlorite and hydrogen peroxide, compared to non-oxidizing biocides (sulfathiazole and glutaraldehyde) (327, 328). Reverse-amide class of 2-aminoimidazole compounds were also found to inhibit *A. baumannii* biofilms by >95% at 100 μM and had the potential to readily increase the permeation of many conventional antibiotics into the bacterial cell membrane, therefore can be used as a new “drug delivery” mechanism in a variety of systems (329). The efficacy of octenidine dihydrochloride (OH) (disinfectant) was tested to reduce *A. baumannii* biofilms on polystyrene, stainless steel, catheters and was found to be effective in inhibiting *A. baumannii* biofilms at a concentration of 5, 10, and 15 mM (330). Biocidal activity of commonly used antiseptics and disinfectants [sodiumhypochlorite, chlorhexidine, orthophthalaldehyde (OPA), peracetic acid (PA), and peracetic acid] were tested against *A. baumanni*. About 78% of biofilm-producing *A. baumannii* became susceptible to all disinfectants and antiseptics tested (331). *A. baumannii* growth and biofilm formation in human serum was reduced by 16 μM of gallium nitrate (hydrated nitrate salt), whereas a higher concentration (64 μM) caused huge disruption of the preformed *A. baumannii* biofilms (332). *N*^2^, *N*^4^-disubstituted quinazoline-2,4-diamines which are dihydrofolate reductase inhibitors exhibited anti-biofilm activity (90%) against *A. baumannii* when the 6-position is replaced with a halide or an alkyl substituent (333). A clinical trial showed that the use of silver alloy on catheter material reduced bacterial colonization, thereby reducing CAUTIs (334). Different clinical trials showed that in addition to silver alloy-coated latex catheters, nitrofurazone-coated silicon catheters were also found to reduce CAUTI during short-term use (<30 days) (335).

## Surface Modification and Physical Therapy

Surface modification and physical therapy are the most successful treatment options for eradication or prevention of microbial adhesion on medical devices when antibiotic treatment fails to effectively eliminate medical device-related biofilm infections. An effective physical method is photodynamic therapy (PDT) for removing microbial adhesion and biofilm formation. PDT inhibits biofilm formation and also fights against biofilm infections by producing Reactive Oxygen Species (ROS) and inhibiting the production of some toxic factors that affect bacterial adhesion and biofilm matrix formation (336, 337). PDT technique is also used to combat other implant-related biofilm infections, such as prosthetic joint infections and infections caused by ventilator-associated pneumonia biofilms (338). Low-intensity ultrasound at the physiotherapy level showed effective removal of biofilm by enhancing the activity of antibacterial agents (339). Another effective physical method is the use of water jets for removing biofilms on the surface of implants through the mechanical action of pulse and pressure (340).

Advances in surface engineering have led to the development of antibacterial agents or antiadhesion agents to coat the surface of medical devices that can effectively inhibit the growth of microorganisms. The use of Sharklet™ surface made by shark skin and microscale ribs of various lengths are combined into a repeating diamond micropattern, preventing bacterial colonization and biofilm formation when incorporated into the surfaces of medical devices (341). Several antibacterial coatings, such as chlorhexidine, rifampicin, gentamicin, minocycline, silver sulfadiazine, amikacin, and vancomycin, have been widely used in clinical practice, showing the efficacy of preventing catheter-related and other implant-related infections (342). Hydrophilic polymers such as hyaluronic acid have been used to coat different medically relevant materials like polyurethane catheters and silicon shunts and have been shown to be effective in preventing biofilm formation on polystyrene surfaces. An isoeugenol coating has been shown to prevent the adhesion and formation of biofilms on stainless steel and polyethylene surfaces due to its good antibacterial activity (343). Various hydrogel coatings, heparin coatings, or bindings have been used in medical devices to reduce fibronectin deposition on vascular catheter surfaces due to high antiadhesive activity (344). In addition, inhibition of microbial growth on catheters also depends on the catheter constitutive polymers which are able to absorb large amounts of antibiotics. These polymers can be designed by introducing acidic or basic groups into the polymer side chains that are able to interact with different classes of drugs (345). Polyethylene glycol and bovine albumin could be incorporated into the polymer bulk together with antibiotic or antifungal molecules to increase and control drug release from the polymer matrices.

## Discussion

The genus *Acinetobacter* is not easy to deal with, both for researchers and clinicians alike, starting with the identification of the multiple species to its propensity to form biofilms and acquire antibiotic resistance genes readily (13, 30). *A. baumannii* has been particularly difficult in ICUs and notorious global clones ST1 and ST2 have also been reported from cases of neonatal sepsis (346, 347). Of the different species “baumannii” is most frequently reported; however, other species of *Acinetobacter* are also being reported to cause infections (348). The role of “non-baumannii” in human infection has been perceived recently because of the technological advances that allow correct identification of the bacteria at the species level. Identification of the bacteria at the species level heavily depends on the molecular methods or MALDI-TOF. The identification of the species is particularly challenging in low middle-income countries which lack the requisite infrastructure to differentiate the several species. Furthermore, *A. baumannii* and non-baumannii have distinct resistance mechanisms for antimicrobial agents though phenotypically they seem to be undifferentiated. Though this review primarily focused on baumannii, non-baumannii isolates could be lurching behind and due attention should be paid to them.

Despite all the effort that mankind has made to improve and save lives, it has been seen that microbes have found new strategies every time to evade technological or medical advancements. With the additional challenge of antibiotic resistance, new approaches need to be utilized to restrict the usage of antibiotics. Some remedies (such as antibiotics, nano antimicrobial compounds, natural products, bacteriophages) that directly act on pathogens have already been discussed in this review but antimicrobial remedies that act on the host (several innate immune-enhancing peptides) to potentiate antibiotic action should be explored (349). Similarly, probiotics also confer a health benefit on the host against MDR *A. baumannii* when administered in adequate amounts combined with immunomodulators, such as lysophosphatidylcholine and antibiotics such as clarithromycin, colistin, tigecycline, or imipenem by stimulating the immune response (350–352). Though advances in medical science are appreciated, there is no underestimating the power of prevention. Hospital infection control is as relevant as in earlier times and can be important in reducing nosocomial infections and interrupting the transmission chain of pathogens such as *Acinetobacter*.

The ability of *Acinetobacter* to attach to biotic and abiotic surfaces is useful for the organism, particularly in hospitals. It can colonize the patients (sometimes without any symptoms), caregivers, and hospital surfaces or devices that increase the chances of infection. This realization has prompted several studies on approaches to prevent the formation of biofilms in hospital devices. The approaches are diverse ranging from antibiotic-coated devices, quorum sensing quenchers, natural products, phage cocktails and nanoparticles. However, very few of these approaches have been tested in *in vivo* models (288, 289) and reached the patient; most are *in vitro* studies, which have not been carried forward. *In vivo* studies address many of the shortcomings of *in vitro* studies and one can better evaluate the safety, toxicity, and efficacy of a drug candidate in *in vivo* models. Thereby, emphasis needs to be on *in vivo* models and further clinical trials.

Development of persister cells, antibiotic tolerance, population heterogeneity, and biofilm-related infections should be considered as significant risk factors in the course of choosing an appropriate therapy particularly in the case of *A. baumannii*. The possible role of the diversity of its reservoirs, its resistance to drying, its capacity to accumulate resistance genes especially plasmid-mediated resistance genes are challenges associated with this organism. Plasmids carrying carbapenem-resistant genes such as *bla*_NDM_ also carry other resistant genes, which make a panel of antibiotics ineffective. The evolution of new variants of antibiotic-hydrolyzing enzymes, which have better efficiency and better stability, also has made it difficult to treat MDR strains, especially *A. baumannii*.

As the authors reviewed the aspect of biofilm formation alongside antibiotic resistance in *Acinetobacter*, it was realized that there is still a lack of clarity regarding the association. It is presumed that this controversy prevails due to the methodology used to test the association. Most studies have used clinical *Acinetobacter* isolates, carried out *in vitro* biofilm assays, and tested the antibiotic susceptibility separately. As most clinical isolates are also antibiotic-resistant (independent of their biofilm-forming capability) such studies may not be appropriate in understanding the association. The difference in the susceptibility of the isolates to antibiotics, when studied as planktonic growth as well as biofilms, will probably give a better understanding. As the entry of antibiotics is restricted within biofilms due to several mechanisms such as the presence of EPS matrix, antibiotic degradation, *Acinetobacter* as biofilms is resistant to the action of antibiotics but this phenomenon is irrespective of the presence of resistance determinants present.

Whole-genome sequencing has generated a vast amount of data that has enriched our understanding of pathogens. Analysis of such data can build an understanding of the epidemic clones, the ARGs or MGEs that are prevalent, their associations with particular clones, etc. The genome data can also be utilized to understand the strengths and weaknesses of antimicrobial agents. However, sequences are mere blueprints and different *in vivo* and *in vitro* laboratory assays need to be carried out along with genome sequences to understand potential therapeutic options. The focus should be on robust assays from the leads that are already identified.

## Conclusion

Most GNB have an underlying similarity, yet there are several features unique to each, making some of these bacteria such as *A. baumannii* more challenging than others. The determinants that drive any organism to be a successful pathogen are a consequence of several diverse factors. These factors include antibiotic use, infection control practices, climate change, human behavior, deforestation, availability of resources, and several others, that can over the years determine how these pathogens evolve. Future research will increase our understanding of this pathogen. The clinicians' experience of treating patients with *Acinetobacter* infection can further strengthen our future approaches to treatment. Harnessing the “clinical eye” with the “scientific fervor” may help devise new strategies to deal with a pathogen that has various tools at its disposal. The use of antimicrobials in hospitals dealing with COVID-19 patients has increased. However, the attention that antibiotic resistance has been drawing for the past few years has waned off, at least temporarily. The strides that we have taken in controlling resistance need to be fostered to effectively control *A. baumannii* infections.

## Author Contributions

SR and GC performed literature search, wrote the manuscript, and prepared all the figures and tables. SB conception, design, wrote the introduction, and discussion along with a critical revision of the manuscript. AM and SD critically revised the manuscript. All authors read and approved the final manuscript.

## Funding

This study was supported by the Indian Council of Medical Research (ICMR) intramural funding. SR was supported by a fellowship (Senior Research Associateship) from the Council of Scientific and Industrial Research (CSIR). GC was supported by a fellowship (Scientist C) from Okayama University, Okayama.

## Conflict of Interest

The authors declare that the research was conducted in the absence of any commercial or financial relationships that could be construed as a potential conflict of interest.

## Publisher's Note

All claims expressed in this article are solely those of the authors and do not necessarily represent those of their affiliated organizations, or those of the publisher, the editors and the reviewers. Any product that may be evaluated in this article, or claim that may be made by its manufacturer, is not guaranteed or endorsed by the publisher.

## References

[1] WHO. Global Antimicrobial Resistance Surveillance System (GLASS) Report: Early Implementation 2016–2017. Geneva: World Health Organization (2017).

[2] Asokan GV Ramadhan T Ahmed E Sanad H. WHO global priority pathogens list: a bibliometric analysis of medline-pubmed for knowledge mobilization to infection prevention and control practices in Bahrain. Oman Med J. (2019) 34:184–93. 10.5001/omj.2019.37 31110624 PMC6505350

[3] Centers for Disease Control and Prevention. Antibiotic resistant threats in the United States 2019. (2019). Available online at: https://www.cdc.gov/drugresistance/pdf/threats-report/2019-ar-threats-report-508.pdf (accessed January 15, 2020).

[4] Peleg AY Seifert H Paterson DL. *Acinetobacter baumannii*: emergence of a successful pathogen. Clin Microbiol Rev. (2008) 21:538–82. 10.1128/CMR.00058-07 18625687 PMC2493088

[5] Xiao D Wang L Zhang D Xiang D Liu Q Xing X. Prognosis of patients with *Acinetobacter baumannii* infection in the intensive care unit: a retrospective analysis. Exp Ther Med. (2017) 13:1630–3. 10.3892/etm.2017.4137 28413520 PMC5377569

[6] Weinberg SE Villedieu A Bagdasarian N Karah N Teare L Elamin WF. Control and management of multidrug resistant *Acinetobacter baumannii*: a review of the evidence and proposal of novel approaches. Infect Prev Pract. (2020) 2:100077. 10.1016/j.infpip.2020.100077 34368717 PMC8336160

[7] Dijkshoorn L Nemec A Seifert H. An increasing threat in hospitals: multidrug-resistant *Acinetobacter baumannii*. Nat Rev Microbiol. (2007) 5:939–51. 10.1038/nrmicro1789 18007677

[8] Eveillard M Kempf M Belmonte O Pailhories H Joly-Guillou ML. Reservoirs of *Acinetobacter baumannii* outside the hospital and potentialinvolvement in emerging human community-acquired infections. Int J Infect Dis. (2013) 17:e802–5. 10.1016/j.ijid.2013.03.021 23672981

[9] Howard A O'Donoghue M Feeney A Sleator RD. *Acinetobacter baumannii*: an emerging opportunistic pathogen. Virulence. (2012) 3:243–50. 10.4161/viru.19700 22546906 PMC3442836

[10] Lin MF Lan CY. Antimicrobial resistance in *Acinetobacter baumannii*: from bench to bedside. World J Clin Cases. (2014) 16:787–814. 10.12998/wjcc.v2.i12.787 25516853 PMC4266826

[11] Kurcik-Trajkovska B. *Acinetobacter spp.* - a serious enemy threatening hospitals worldwide. Maced J Med Sci. (2009) 2:157–62. 10.3889/MJMS.1857-5773.2009.0043

[12] Litzler PY Benard L Barbier-Frebourg N Vilain S Jouenne T Beucher E. Biofilm formation on pyrolytic carbon heart valves: influence of surface free energy, roughness, and bacterial species. J Thorac Cardiovasc Surg. (2007) 134:1025–32. 10.1016/j.jtcvs.2007.06.013 17903524

[13] Eze EC Chenia HY El Zowalaty ME. *Acinetobacter baumannii* biofilms: effects of physicochemical factors, virulence, antibiotic resistance determinants, gene regulation, and future antimicrobial treatments. Infect Drug Resist. (2018) 11:2277–99. 10.2147/IDR.S169894 30532562 PMC6245380

[14] Youn Sung J. Molecular characterization and antimicrobial susceptibility of biofilm-forming *Acinetobacter baumannii* clinical isolates from Daejeon, Korea. Korean J Clin Lab Sci. (2018) 50:100–9. 10.15324/kjcls.2018.50.2.100

[15] Martí S Rodríguez-Baño J Catel-Ferreira M Jouenne T Vila J Seifert H. Biofilm formation at the solid-liquid and air-liquid interfaces by *Acinetobacter* species. BMC Res Notes. (2011) 4:5. 10.1186/1756-0500-4-5 21223561 PMC3023692

[16] Zeighami H Valadkhani F Shapouri R Samadi E Haghi F. Virulence characteristics of multidrug resistant biofilm forming *Acinetobacter baumannii* isolated from intensive care unit patients. BMC Infect Dis. (2019) 19:629. 10.1186/s12879-019-4272-0 31315572 PMC6637494

[17] Giles SK Stroeher UH Eijkelkamp BA Brown MH. Identification of genes essential for pellicle formation in *Acinetobacter baumannii*. BMC Microbiol. (2015) 15:116. 10.1186/s12866-015-0440-6 26047954 PMC4457973

[18] Pompilio A Scribano D Sarshar M Di Bonaventura G Palamara AT Ambrosi C. Gram-negative bacteria holding together in a biofilm: the *Acinetobacter baumannii* way. Microorganisms. (2021) 9:1353. 10.3390/microorganisms9071353 34206680 PMC8304980

[19] Jacobs AC Blanchard CE Catherman SC Dunman PM Murata Y. An ribonuclease T2 family protein modulates *Acinetobacter baumannii* abiotic surface colonization. PLoS ONE. (2014) 9:e85729. 10.1371/journal.pone.0085729 24489668 PMC3904860

[20] Dallo SF Weitao T. Insights into acinetobacter war-wound infections, biofilms, and control. Adv Skin Wound Care. (2010) 23:169–74. 10.1097/01.ASW.0000363527.08501.a3 20299843

[21] Gedefie A Demsis W Ashagrie M Kassa Y Tesfaye M Tilahun M. *Acinetobacter baumannii* biofilm formation and its role in disease pathogenesis: a review. Infect Drug Resist. (2021) 14:3711–9. 10.2147/IDR.S332051 34531666 PMC8439624

[22] Chapartegui-González I Lázaro-Díez M Bravo Z Navas J Icardo JM Ramos-Vivas J. *Acinetobacter baumannii* maintains its virulence after long-time starvation. PLoS ONE. (2018) 13:e0201961. 10.1371/journal.pone.0201961 30133491 PMC6104976

[23] Hendrickx L Hausner M Wuertz S. Natural genetic transformation in monoculture *Acinetobacter* sp. strain BD413 biofilms. Appl Environ Microbiol. (2003) 69:1721–7. 10.1128/AEM.69.3.1721-1727.2003 12620864 PMC150042

[24] Tanner WD Atkinson RM Goel RK Toleman MA Benson LS Porucznik CA. Horizontal transfer of the blaNDM-1 gene to *Pseudomonas aeruginosa* and *Acinetobacter baumannii* in biofilms. FEMS Microbiol Lett. (2017) 364:fnx048. 10.1093/femsle/fnx048 28333234

[25] Pande S Shitut S Freund L Westermann M Bertels F Colesie C. Metabolic cross-feeding via intercellular nanotubes among bacteria. Nat Commun. (2015) 6:6238. 10.1038/ncomms7238 25703793

[26] Krahn T Wibberg D Maus I Winkler A Bontron S Sczyrba A. Intraspecies transfer of the chromosomal *Acinetobacter baumannii* blaNDM-1 carbapenemase gene. Antimicrob Agents Chemother. (2016) 60:3032–40. 10.1128/AAC.00124-16 26953198 PMC4862468

[27] Wachino JI Jin W Kimura K Arakawa Y. Intercellular transfer of chromosomal antimicrobial resistance genes between *Acinetobacter baumannii* strains mediated by prophages. Antimicrob Agents Chemother. (2019) 63:e00334–19. 10.1128/AAC.00334-19 31138576 PMC6658751

[28] Rumbo C Fernández-Moreira E Merino M Poza M Mendez JA Soares NC. Horizontal transfer of the OXA-24 carbapenemase gene via outer membrane vesicles: a new mechanism of dissemination of carbapenem resistance genes in *Acinetobacter baumannii*. Antimicrob Agents Chemother. (2011) 55:3084–90. 10.1128/AAC.00929-10 21518847 PMC3122458

[29] Chatterjee S Mondal A Mitra S Basu S. *Acinetobacter baumannii* transfers the blaNDM-1 gene via outer membrane vesicles. J Antimicrob Chemother. (2017) 72:2201–7. 10.1093/jac/dkx131 28505330

[30] Kyriakidis I Vasileiou E Pana ZD Tragiannidis A. *Acinetobacter baumannii* antibiotic resistance mechanisms. Pathogens. (2021) 10:373. 10.3390/pathogens10030373 33808905 PMC8003822

[31] Qi L Li H Zhang C Liang B Li J Wang L. Relationship between antibiotic resistance, biofilm formation, and biofilm-specific resistance in *Acinetobacter baumannii*. Front Microbiol. (2016) 7:483. 10.3389/fmicb.2016.00483 27148178 PMC4828443

[32] Colquhoun JM Rather PN. Insights into mechanisms of biofilm formation in *Acinetobacter baumannii* and implications for uropathogenesis. Front Cell Infect Microbiol. (2020) 10:253. 10.3389/fcimb.2020.00253 32547965 PMC7273844

[33] Dijkshoorn L. The diversity of the genus *Acinetobacter*. In: Gerischer U, editor. Acinetobacter Molecular Biology. CaisterAcademic Press (2008).

[34] Munoz-Price LS Weinstein RA. Acinetobacter infection. N Engl J Med. (2008) 358:1271–81. 10.1056/NEJMra070741 18354105

[35] Brisou J Prevot A. Studies on bacterial taxonomy. X. The revision of species under Acromobacter group. Ann Inst Pasteur. (1954) 86:722–8. 13197842

[36] Chuang YC Sheng WH Li SY Lin YC Wang JT Chen YC. Influence of genospecies of *Acinetobacter baumannii* complex on clinical outcomes of patients with acinetobacter bacteremia. Clin Infect Dis. (2011) 52:352–60. 10.1093/cid/ciq154 21193494

[37] Bergogne-Bérézin E Towner KJ. *Acinetobacter spp.* as nosocomial pathogens: microbiological, clinical, and epidemiological features. Clin Microbiol Rev. (1996) 9:148–65. 10.1128/CMR.9.2.148 8964033 PMC172888

[38] Morohoshi T Saito T. beta-Lactamase and beta-lactam antibiotics resistance in acinetobacter anitratum (syn: *A. calcoaceticus)*. J Antibiot. (1977) 30:969–73. 10.7164/antibiotics.30.969 201599

[39] Paton R Miles RS Hood J Amyes SG Miles RS Amyes SG. ARI 1: beta-lactamase-mediated imipenem resistance in *Acinetobacter baumannii*. Int J Antimicrob Agents. (1993) 2:81–7. 10.1016/0924-8579(93)90045-7 18611526

[40] Higgins PG Dammhayn C Hackel M Seifert H. Global spread of carbapenem-resistant *Acinetobacter baumannii*. J Antimicrob Chemother. (2010) 65:233–8. 10.1093/jac/dkp428 19996144

[41] Hamidian M Nigro SJ. Emergence, molecular mechanisms and global spread of carbapenem-resistant *Acinetobacter baumannii*. Microb Genom. (2019) 5:e000306. 10.1099/mgen.0.000306 31599224 PMC6861865

[42] Hejnar P Kolár M Hájek V. Characteristics of Acinetobacter strains (phenotype classification, antibiotic susceptibility and production ofbeta-lactamases) isolated from haemocultures from patients at theTeaching Hospital in Olomouc. Acta Univ Palacki Olomuc Fac Med. (1999) 142:73–77. 10743729

[43] Cai Y Chai D Wang R Liang B Bai N. Colistin resistance of *Acinetobacter baumannii*: clinical reports, mechanisms and antimicrobial strategies. J Antimicrob Chemother. (2012) 67:1607–15. 10.1093/jac/dks084 22441575

[44] Sader HS Jones RN Stilwell MG Dowzicky MJ Fritsche TR. Tigecycline activity tested against 26,474 bloodstream infection isolates: a collection from 6 continents. Diagn Microbiol Infect Dis. (2005) 52:181–6. 10.1016/j.diagmicrobio.2005.05.005 16105562

[45] Stein GE Babinchak T. Tigecycline: an update. Diagn Microbiol Infect Dis. (2013) 75:331–6. 10.1016/j.diagmicrobio.2012.12.004 23357291

[46] Navon-Venezia S Leavitt A Carmeli Y. High tigecycline resistance in multidrug-resistant *Acinetobacter baumannii*. J Antimicrob Chemother. (2007) 59:772–4. 10.1093/jac/dkm018 17353223

[47] Gonzalez-Villoria AM Valverde-Garduno V. Antibiotic-resistant *Acinetobacter baumannii* increasing success remains a challenge as a nosocomial pathogen. J Pathog. (2016) 2016:7318075. 10.1155/2016/7318075 26966582 PMC4757776

[48] Kisil OV Efimenko TA Gabrielyan NI Efremenkova OV. Development of antimicrobial therapy methods to overcome the antibiotic resistance of *Acinetobacter baumannii*. Acta Naturae. (2020) 12:34–45. 10.32607/actanaturae.10955 33173595 PMC7604900

[49] Clatworthy AE Pierson E Hung DT. Targeting virulence: a new paradigm for antimicrobial therapy. Nat Chem Biol. (2007) 3:541–8. 10.1038/nchembio.2007.24 17710100

[50] Ramirez MS Don M Merkier AK Bistué AJ Zorreguieta A Centrón D. Naturally competent *Acinetobacter baumannii* clinical isolate as a convenient model for genetic studies. J Clin Microbiol. (2010) 48:1488–90. 10.1128/JCM.01264-09 20181905 PMC2849597

[51] Touchon M Cury J Yoon EJ Krizova L Cerqueira GC Murphy C. The genomic diversification of the whole Acinetobacter genus: origins, mechanisms, and consequences. Genome Biol Evol. (2014) 6:2866–82. 10.1093/gbe/evu225 25313016 PMC4224351

[52] Traglia GM Chua K Centron D Tolmasky ME Ramirez MS. Whole-genome sequence analysis of the naturally competent *Acinetobacter baumannii* clinical isolate A118. Genome Biol Evol. (2014) 6:2235–9. 10.1093/gbe/evu176 25164683 PMC4202317

[53] Perez F Hujer AM Hujer KM Decker BK Rather PN Bonomo RA. Global challenge of multidrug-resistant *Acinetobacter baumannii*. Antimicrob Agents Chemother. (2007) 51:3471–84. 10.1128/AAC.01464-06 17646423 PMC2043292

[54] Munita JM Arias CA. Mechanisms of antibiotic resistance. Microbiol Spectr. (2016) 4:2015. 10.1128/microbiolspec.VMBF-0016-2015 27227291 PMC4888801

[55] Ambler RP. The structure of beta-lactamases. Philos Trans R Soc Lond B Biol Sci. (1980) 289:321–31. 10.1098/rstb.1980.0049 6109327

[56] Naas T Dortet L Iorga BI. Structural and functional aspects of class a carbapenemases. Curr Drug Targets. (2016) 17:1006–28. 10.2174/1389450117666160310144501 26960341 PMC5405625

[57] Alyamani EJ Khiyami MA Booq RY Alnafjan BM Altammami MA Bahwerth FS. Molecular characterization of extended-spectrum beta-lactamases (ESBLs) produced by clinical isolates of *Acinetobacter baumannii* in Saudi Arabia. Ann Clin Microbiol Antimicrob. (2015) 14:38. 10.1186/s12941-015-0098-9 26290183 PMC4545919

[58] Abdar MH Taheri-Kalani M Taheri K Emadi B Hasanzadeh A Sedighi A. Prevalence of extended-spectrum beta-lactamase genes in *Acinetobacter baumannii* strains isolated from nosocomial infections in Tehran, Iran. GMS Hyg Infect Control. (2019) 14:318. 10.3205/dgkh000318 30834190 PMC6388673

[59] Hamidian M Hall RM. Resistance to third-generation cephalosporins in *Acinetobacter baumannii* due to horizontal transfer of a chromosomal segment containing ISAba1-ampC. J Antimicrob Chemother. (2014) 69:2865–6. 10.1093/jac/dku202 24917581

[60] Lee CR Lee JH Park M Park KS Bae IK Kim YB. Biology of *Acinetobacter baumannii*: pathogenesis, antibiotic resistance mechanisms, and prospective treatment options. Front Cell Infect Microbiol. (2017) 7:55. 10.3389/fcimb.2017.00055 28348979 PMC5346588

[61] Jeon JH Lee JH Lee JJ Park KS Karim AM Lee CR. Structural basis for carbapenem-hydrolyzing mechanisms of carbapenemases conferring antibiotic resistance. Int J Mol Sci. (2015) 16:9654–92. 10.3390/ijms16059654 25938965 PMC4463611

[62] Aksoy MD Çavuşlu S Tugrul HM. Investigation of metallo beta lactamases and oxacilinases in carbapenem resistant *Acinetobacter baumannii* strains isolated from inpatients. Balkan Med J. (2015) 32:79–83. 10.5152/balkanmedj.2015.15302 25759776 PMC4342142

[63] Périchon B Goussard S Walewski V Krizova L Cerqueira G Murphy C. Identification of 50 class D β-lactamases and 65 Acinetobacter-derived cephalosporinases in *Acinetobacter spp*. Antimicrob Agents Chemother. (2014) 58:936–49. 10.1128/AAC.01261-13 24277043 PMC3910822

[64] Brown S Amyes S. OXA (beta)-lactamases in Acinetobacter: the story so far. J Antimicrob Chemother. (2006) 57:1–3. 10.1093/jac/dki425 16332731

[65] Turton JF Woodford N Glover J Yarde S Kaufmann ME Pitt TL. Identification of *Acinetobacter baumannii* by detection of the blaOXA-51-like carbapenemase gene intrinsic to this species. J Clin Microbiol. (2006) 44:2974–6. 10.1128/JCM.01021-06 16891520 PMC1594603

[66] Vijayakumar S Gopi R Gunasekaran P Bharathy M Walia K Anandan S. Molecular characterization of invasive carbapenem-resistant *Acinetobacter baumannii* from a Tertiary Care Hospital in South India. Infect Dis Ther. (2016) 5:379–87. 10.1007/s40121-016-0125-y 27553951 PMC5019981

[67] Vrancianu CO Gheorghe I Czobor IB Chifiriuc MC. Antibiotic resistance profiles, molecular mechanisms and innovative treatment strategies of *Acinetobacter baumannii*. Microorganisms. (2020) 8:935. 10.3390/microorganisms8060935 32575913 PMC7355832

[68] Bonnin RA Poirel L Naas T Pirs M Seme K Schrenzel J. Dissemination of New Delhi metallo-β-lactamase-1-producing *Acinetobacter baumannii* in Europe. Clin Microbiol Infect. (2012) 18:E362–5. 10.1111/j.1469-0691.2012.03928.x 22738206

[69] Poirel L Nordmann P. Carbapenem resistance in *Acinetobacter baumannii*: mechanisms and epidemiology. Clin Microbiol Infect. (2006) 12:826–36. 10.1111/j.1469-0691.2006.01456.x 16882287

[70] Nikibakhsh M Firoozeh F Badmasti F Kabir K Zibaei M. Molecular study of metallo-β-lactamases and integrons in *Acinetobacter baumannii* isolates from burn patients. BMC Infect Dis. (2021) 21:782. 10.1186/s12879-021-06513-w 34372787 PMC8353788

[71] Rahman M Prasad KN Gupta S Singh S Singh A Pathak A. Prevalence and molecular characterization of New Delhi metallo-beta-lactamases in multidrug-resistant *Pseudomonas aeruginosa* and *Acinetobacter baumannii* from India. Microb Drug Resist. (2018) 24:792–98. 10.1089/mdr.2017.0078 29058515

[72] Coyne S Courvalin P Périchon B. Efflux-mediated antibiotic resistance in *Acinetobacter spp*. Antimicrob Agents Chemother. (2011) 55:947–53. 10.1128/AAC.01388-10 21173183 PMC3067115

[73] Ribera A Roca I Ruiz J Gibert I Vila J. Partial characterization of a transposon containing the tet(A) determinant in a clinical isolate of *Acinetobacter baumannii*. J Antimicrob Chemother. (2003) 52:477–80. 10.1093/jac/dkg344 12888597

[74] ELsheredy A Yousif Z Elghazzawi E Elmenshawy A Ghazal A. Prevalence of genes encoding aminoglycoside-modifying enzymes and armA among *Acinetobacter baumannii* clinical isolates in alexandria, Egypt. Infect Disord Drug Targets. (2021) 24:3041. 10.2174/1871526521666210225113041 33632111

[75] Vashist J Tiwari V Das R Kapil A Rajeswari MR. Analysis of penicillin-binding proteins (PBPs) in carbapenem resistant *Acinetobacter baumannii*. Indian J Med Res. (2011) 133:332–8. 21441690 PMC3103161

[76] Vila J Martí S Sánchez-Céspedes J. Porins, efflux pumps and multidrug resistance in *Acinetobacter baumannii*. J Antimicrob Chemother. (2007) 59:1210–5. 10.1093/jac/dkl509 17324960

[77] Costerton JW Stewart PS Greenberg EP. Bacterial biofilms: a common cause of persistent infections. Science. (1999) 284:1318–22. 10.1126/science.284.5418.1318 10334980

[78] Stoodley P Hall-Stoodley L Lappin-Scott HM. Detachment, surface migration, and other dynamic behavior in bacterial biofilms revealed by digital time-lapse imaging. Methods Enzymol. (2001) 337:306–19. 10.1016/s0076-6879(01)37023-4 11398439

[79] Loehfelm TW Luke NR Campagnari AA. Identification and characterization of an *Acinetobacter baumannii* biofilm-associated protein. J Bacteriol. (2008) 190:1036–44. 10.1128/JB.01416-07 18024522 PMC2223572

[80] De Gregorio E Del Franco M Martinucci M Roscetto E Zarrilli R Di Nocera PP. Biofilm-associated proteins: news from Acinetobacter. BMC Genomics. (2015) 16:933. 10.1186/s12864-015-2136-6 26572057 PMC4647330

[81] Itoh Y Rice JD Goller C Pannuri A Taylor J Meisner J. Roles of pgaABCD genes in synthesis, modification, and export of the *Escherichia coli* biofilm adhesin poly-beta-1,6-N-acetyl-D-glucosamine. J Bacteriol. (2008) 190:3670–80. 10.1128/JB.01920-07 18359807 PMC2394981

[82] Flannery A Le Berre M Pier GB O'Gara JP Kilcoyne M. Glycomics microarrays reveal differential *in situ* presentation of the biofilm polysaccharide poly-*N*-acetylglucosamine on *Acinetobacter baumannii* and *Staphylococcus aureus* cell surfaces. Int J Mol Sci. (2020) 21:2465. 10.3390/ijms21072465 32252300 PMC7177611

[83] Lee HW Koh YM Kim J Lee JC Lee YC Seol SY. Capacity of multidrug-resistant clinical isolates of *Acinetobacter baumannii* to form biofilm and adhere to epithelial cell surfaces. Clin Microbiol Infect. (2008) 14:49–54. 10.1111/j.1469-0691.2007.01842.x 18005176

[84] Pakharukova N Tuittila M Paavilainen S Malmi H Parilova O Teneberg S. Structural basis for *Acinetobacter baumannii* biofilm formation. Proc Natl Acad Sci USA. (2018) 115:5558–63. 10.1073/pnas.1800961115 29735695 PMC6003481

[85] Gaddy JA Actis LA. Regulation of *Acinetobacter baumannii* biofilm formation. Future Microbiol. (2009) 4:273–8. 10.2217/fmb.09.5 19327114 PMC2724675

[86] Gaddy JA Tomaras AP Actis LA. The *Acinetobacter baumannii* 19606 OmpA protein plays a role in biofilm formation on abiotic surfaces and in the interaction of this pathogen with eukaryotic cells. Infect Immun. (2009) 77:3150–60. 10.1128/IAI.00096-09 19470746 PMC2715673

[87] Bhargava N Sharma P Capalash N. Quorum sensing in *Acinetobacter*: an emerging pathogen. Crit Rev Microbiol. (2010) 36:349–60. 10.3109/1040841X.2010.512269 20846031

[88] López-Martín M Dubern JF Alexander MR Williams P. AbaM regulates quorum sensing, biofilm formation, and virulence in *Acinetobacter baumannii*. J Bacteriol. (2021) 203:e00635–20. 10.1128/JB.00635-20 33495249 PMC8088503

[89] Lasa I Penadés JR. Bap: a family of surface proteins involved in biofilm formation. Res Microbiol. (2006) 157:99–107. 10.1016/j.resmic.2005.11.003 16427771

[90] Azizi O Shahcheraghi F Salimizand H Modarresi F Shakibaie MR Mansouri Sh. Molecular analysis and expression of *bap* gene in biofilm-forming multi-drug-resistant *Acinetobacter baumannii*. Rep Biochem Mol Biol. (2016) 5:62–72. 28070537 PMC5214686

[91] Choi AH Slamti L Avci FY Pier GB Maira-Litrán T. The pgaABCD locus of *Acinetobacter baumannii* encodes the production of poly-beta-1-6-N-acetylglucosamine, which is critical for biofilm formation. J Bacteriol. (2009) 191:5953–63. 10.1128/JB.00647-09 19633088 PMC2747904

[92] Rao RS Karthika RU Singh SP Shashikala P Kanungo R Jayachandran S. Correlation between biofilm production and multiple drug resistance in imipenem resistant clinical isolates of *Acinetobacter baumannii*. Indian J Med Microbiol. (2008) 26:333–7. 10.4103/0255-0857.43566 18974485

[93] Khamari B Lama M Pachi Pulusu C Biswal AP Lingamallu SM Mukkirla BS. Molecular analyses of biofilm-producing clinical *Acinetobacter baumannii* isolates from a South Indian Tertiary Care Hospital. Med Princ Pract. (2020) 29:580–7. 10.1159/000508461 32380504 PMC7768151

[94] Bardbari AM Arabestani MR Karami M Keramat F Alikhani MY Bagheri KP. Correlation between ability of biofilm formation with their responsible genes and MDR patterns in clinical and environmental *Acinetobacter baumannii* isolates. Microb Pathog. (2017) 108:122–8. 10.1016/j.micpath.2017.04.039 28457900

[95] Tomaras AP Dorsey CW Edelmann RE Actis LA. Attachment to and biofilm formation on abiotic surfaces by *Acinetobacter baumannii*: involvement of a novel chaperone-usher pili assembly system. Microbiology. (2003) 149:3473–84. 10.1099/mic.0.26541-0 14663080

[96] Tomaras AP Flagler MJ Dorsey CW Gaddy JA Actis LA. Characterization of a two-component regulatory system from *Acinetobacter baumannii* that controls biofilm formation and cellular morphology. Microbiology. (2008) 154:3398–409. 10.1099/mic.0.2008/019471-0 18957593

[97] Harding CM Hennon SW Feldman MF. Uncovering the mechanisms of *Acinetobacter baumannii* virulence. Nat Rev Microbiol. (2018) 16:91–102. 10.1038/nrmicro.2017.148 29249812 PMC6571207

[98] Selasi GN Nicholas A Jeon H Na SH Kwon HI Kim YJ. Differences in biofilm mass, expression of biofilm-associated genes, and resistance to desiccation between epidemic and sporadic clones of carbapenem-resistant *Acinetobacter baumannii* sequence type 191. PLoS ONE. (2016) 11:e0162576. 10.1371/journal.pone.0162576 27622249 PMC5021322

[99] McConnell MJ Actis L Pachón J. *Acinetobacter baumannii*: human infections, factors contributing to pathogenesis and animal models. FEMS Microbiol Rev. (2013) 37:130–55. 10.1111/j.1574-6976.2012.00344.x 22568581

[100] Rumbo C Tomás M Fernández ME Soares NC Carvajal M Santillana E. The *Acinetobacter baumannii* Omp33-36 porin is a virulence factor that induces apoptosis and modulates autophagy in human cells. Infect Immun. (2014) 82:4666–80. 10.1128/IAI.02034-14 25156738 PMC4249306

[101] Ng WL Bassler BL. Bacterial quorum-sensing network architectures. Annu Rev Genet. (2009) 43:197–222. 10.1146/annurev-genet-102108-134304 19686078 PMC4313539

[102] Dou Y Song F Guo F Zhou Z Zhu C Xiang J. *Acinetobacter baumannii* quorum-sensing signalling molecule induces the expression of drug-resistance genes. Mol Med Rep. (2017) 15:4061–8. 10.3892/mmr.2017.6528 28487993 PMC5436197

[103] Oh MH Han K. AbaR is a LuxR type regulator essential for motility and the formation of biofilm and pellicle in *Acinetobacter baumannii*. Genes Genomics. (2020) 42:1339–46. 10.1007/s13258-020-01005-8 33025548

[104] Mayer C Muras A Parga A Romero M Rumbo-Feal S Poza M. Quorum sensing as a target for controlling surface associated motility and biofilm formation in *Acinetobacter baumannii* ATCC® 17978^TM^. Front Microbiol. (2020) 11:565548. 10.3389/fmicb.2020.565548 33101239 PMC7554515

[105] Luo LM Wu LJ Xiao YL Zhao D Chen ZX Kang M. Enhancing pili assembly and biofilm formation in *Acinetobacter baumannii* ATCC19606 using non-native acyl-homoserine lactones. BMC Microbiol. (2015) 15:62. 10.1186/s12866-015-0397-5 25888221 PMC4381447

[106] Rumbo-Feal S Gomez MJ Gayoso C Álvarez-Fraga L Cabral MP Aransay AM. Whole transcriptome analysis of *Acinetobacter baumannii* assessed by RNA-sequencing reveals different mRNA expression profiles in biofilm compared to planktonic cells. PLoS ONE. (2013) 8:e72968. 10.1371/journal.pone.0072968 24023660 PMC3758355

[107] Alav I Sutton JM Rahman KM. Role of bacterial efflux pumps in biofilm formation. J Antimicrob Chemother. (2018) 73:2003–20. 10.1093/jac/dky042 29506149

[108] He X Lu F Yuan F Jiang D Zhao P Zhu J. Biofilm formation caused by clinical *Acinetobacter baumannii* isolates is associated with overexpression of the AdeFGH efflux pump. Antimicrob Agents Chemother. (2015) 59:4817–25. 10.1128/AAC.00877-15 26033730 PMC4505227

[109] Roca I Espinal P Martí S Vila J. First identification and characterization of an AdeABC-like efflux pump in Acinetobacter genomospecies 13TU. Antimicrob Agents Chemother. (2011) 55:1285–6. 10.1128/AAC.01142-10 21199925 PMC3067064

[110] Greene C Wu J Rickard AH Xi C. Evaluation of the ability of *Acinetobacter baumannii* to form biofilms on six different biomedical relevant surfaces. Lett Appl Microbiol. (2016) 63:233–9. 10.1111/lam.12627 27479925 PMC7057210

[111] Eze EC El Zowalaty ME. Combined effects of low incubation temperature, minimal growth medium, and low hydrodynamics optimize *Acinetobacter baumannii* biofilm formation. Infect Drug Resist. (2019) 12:3523–36. 10.2147/IDR.S203919 31814741 PMC6863185

[112] Khatoon Z McTiernan CD Suuronen EJ Mah TF Alarcon EI. Bacterial biofilm formation on implantable devices and approaches to its treatment and prevention. Heliyon. (2018) 4:e01067. 10.1016/j.heliyon.2018.e01067 30619958 PMC6312881

[113] Jones DS Gorman SP. Medical device composition and biological secretion influences on biofilm formation. In: Biofilms, Infection, and Antimicrobial Therapy. CRC Press (2005). p. 62–81.

[114] Dekic S Hrenovic J Ivankovic T van Wilpe E. Survival of ESKAPE pathogen *Acinetobacter baumannii* in water of different temperatures and pH. Water Sci Technol. (2018) 78:1370–6. 10.2166/wst.2018.409 30388093

[115] Pour NK Dusane DH Dhakephalkar PK Zamin FR Zinjarde SS Chopade BA. Biofilm formation by *Acinetobacter baumannii* strains isolated from urinary tract infection and urinary catheters. FEMS Immunol Med Microbiol. (2011) 62:328–38. 10.1111/j.1574-695X.2011.00818.x 21569125

[116] De Silva PM Chong P Fernando DM Westmacott G Kumar A. Effect of incubation temperature on antibiotic resistance and virulence factors of acinetobacter baumannii ATCC 17978. Antimicrob Agents Chemother. (2017) 62:e01514-17. 10.1128/AAC.01514-17 29061747 PMC5740336

[117] Martinez LR Casadevall A. Cryptococcus neoformans biofilm formation depends on surface support and carbon source and reduces fungal cell susceptibility to heat, cold, and UV light. Appl Environ Microbiol. (2007) 73:4592–601. 10.1128/AEM.02506-06 17513597 PMC1932807

[118] Nwugo CC Arivett BA Zimbler DL Gaddy JA Richards AM Actis LA. Effect of ethanol on differential protein production and expression of potential virulence functions in the opportunistic pathogen *Acinetobacter baumannii*. PLoS ONE. (2012) 7:e51936. 10.1371/journal.pone.0051936 23284824 PMC3527336

[119] Gilbert P Das J Foley I. Biofilm susceptibility to antimicrobials. Adv Dent Res. (1997) 11:160–7. 10.1177/08959374970110010701 9524452

[120] Harrison JJ Ceri H Turner RJ. Multimetal resistance and tolerance in microbial biofilms. Nat Rev Microbiol. (2007) 5:928–38. 10.1038/nrmicro1774 17940533

[121] Montanaro L Poggi A Visai L Ravaioli S Campoccia D Speziale P. Extracellular DNA in biofilms. Int J Artif Organs. (2011) 34:824–31. 10.5301/ijao.5000051 22094562

[122] Das T Sharma PK Busscher HJ van der Mei HC Krom BP. Role of extracellular DNA in initial bacterial adhesion and surface aggregation. Appl Environ Microbiol. (2010) 76:3405–8. 10.1128/AEM.03119-09 20363802 PMC2869138

[123] Sahu PK Iyer PS Oak AM Pardesi KR Chopade BA. Characterization of eDNA from the clinical strain *Acinetobacter baumannii* AIIMS 7 and its role in biofilm formation. Sci World J. (2012) 973436. 10.1100/2012/973436 22593716 PMC3346689

[124] Stewart PS Franklin MJ. Physiological heterogeneity in biofilms. Nat Rev Microbiol. (2008) 6:199–210. 10.1038/nrmicro1838 18264116

[125] Brown MR Allison DG Gilbert P. Resistance of bacterial biofilms to antibiotics: a growth-rate related effect? J Antimicrob Chemother. (1988) 22:777–80. 10.1093/jac/22.6.777 3072331

[126] Conlon BP Rowe SE Lewis K. Persister cells in biofilm associated infections. Adv Exp Med Biol. (2015) 831:1–9. 10.1007/978-3-319-09782-4_1 25384659

[127] Desai M Bühler T Weller PH Brown MR. Increasing resistance of planktonic and biofilm cultures of Burkholderia cepacia to ciprofloxacin and ceftazidime during exponential growth. J Antimicrob Chemother. (1998) 42:153–60. 10.1093/jac/42.2.153 9738832

[128] Tipton KA Dimitrova D Rather PN. Phase-variable control of multiple phenotypes in *Acinetobacter baumannii* strain AB5075. J Bacteriol. (2015) 197:2593–9. 10.1128/JB.00188-15 26013481 PMC4518826

[129] Fauvart M De Groote VN Michiels J. Role of persister cells in chronic infections: clinical relevance and perspectives on anti-persister therapies. J Med Microbiol. (2011) 60:699–709. 10.1099/jmm.0.030932-0 21459912

[130] Gayoso CM Mateos J Méndez JA Fernández-Puente P Rumbo C Tomás M. Molecular mechanisms involved in the response to desiccation stress and persistence in *Acinetobacter baumannii*. J Proteome Res. (2014) 13:460–76. 10.1021/pr400603f 24299215

[131] Rittershaus ES Baek SH Sassetti CM. The normalcy of dormancy: common themes in microbial quiescence. Cell Host Microbe. (2013) 13:643–51. 10.1016/j.chom.2013.05.012 23768489 PMC3743100

[132] Harding CM Hennon SW Feldman MF. Uncovering the mechanisms of *Acinetobacter baumannii* virulence. Nat Rev Microbiol. (2018) 16:91–102. 10.1038/nrmicro.2017 29249812 PMC6571207

[133] Barth VC Jr Rodrigues BÁ Bonatto GD Gallo SW Pagnussatti VE Ferreira CA. Heterogeneous persister cells formation in *Acinetobacter baumannii*. PLoS ONE. (2013) 8:e84361. 10.1371/journal.pone.0084361 24391945 PMC3877289

[134] Kushwaha GS Oyeyemi BF Bhavesh NS. Stringent response protein as a potential target to intervene persistent bacterial infection. Biochimie. (2019) 165:67–75. 10.1016/j.biochi.2019.07.006 31302165

[135] Pérez-Varela M Tierney ARP Kim JS Vázquez-Torres A Rather P. Characterization of RelA in *Acinetobacter baumannii*. J Bacteriol. (2020) 202:e00045–20. 10.1128/JB.00045-20 32229531 PMC7253615

[136] Dörr T Lewis K Vulić M. SOS response induces persistence to fluoroquinolones in *Escherichia coli*. PLoS Genet. (2009) 5:e1000760. 10.1371/journal.pgen.1000760 20011100 PMC2780357

[137] Alkasir R Ma Y Liu F Li J Lv N Xue Y. Characterization and transcriptome analysis of *Acinetobacter baumannii* persister cells. Microb Drug Resist. (2018) 24:1466–74. 10.1089/mdr.2017.0341 29902105

[138] Hengge-Aronis R. Regulation of gene expression during entry into stationary phase. In: Neidhardt FC Curtiss R III Ingraham JL Lin ECC Low KB Magasanik B Reznikoff WS Riley M Schaechter M Umbarger HE. Escherichia coli Salmonella: Cellular Molecular Biology, 2nd Edn. Washington, DC: ASM Press (1996). p. 1497–512.

[139] Thummeepak R Kongthai P Leungtongkam U Sitthisak S. Distribution of virulence genes involved in biofilm formation in multi-drug resistant *Acinetobacter baumannii* clinical isolates. Int Microbiol. (2016) 19:121–9. 10.2436/20.1501.01.270 27845499

[140] Yang CH Su PW Moi SH Chuang LY. Biofilm formation in *Acinetobacter Baumannii*: genotype-phenotype correlation. Molecules. (2019) 24:1849. 10.3390/molecules24101849 31091746 PMC6572253

[141] Ranjbar R Farahani A. Study of genetic diversity, biofilm formation, and detection of Carbapenemase, MBL, ESBL, and tetracycline resistance genes in multidrug-resistant *Acinetobacter baumannii* isolated from burn wound infections in Iran. Antimicrob Resist Infect Control. (2019) 8:172. 10.1186/s13756-019-0612-5 31719975 PMC6836547

[142] Celik B. Evaluation of the correlation between biofilm formation and drug resistance in clinical isolates of *Acinetobacter baumannii*. Int J Pathog Res. (2020) 5:16–27. 10.9734/ijpr/2020/v5i130124

[143] Asaad AM Ansari S Ajlan SE Awad SM. Epidemiology of biofilm producing *Acinetobacter baumannii* nosocomial isolates from a tertiary care hospital in Egypt: a cross-sectional study. Infect Drug Resist. (2021) 14:709–17. 10.2147/IDR.S261939 33654415 PMC7914062

[144] Nahar A Anwar S Miah MR. Association of biofilm formation with antimicrobial resistance among the acinetobacter species in a tertiary care hospital in Bangladesh. J Med. (2013) 14:28–32. 10.3329/jom.v14i1.14533

[145] Emami S Eftekhar F. The correlation between biofilm formation and drug resistance in nosocomial isolates of *Acinetobacter baumannii*. Avicenna J Clin Microbiol Infect. (2015) 2:e23954. 10.17795/ajcmi-23954

[146] Rodríguez-Baño J Martí S Soto S Fernández-Cuenca F Cisneros JM Pachón J. Biofilm formation in *Acinetobacter baumannii*: associated features and clinical implications. Clin Microbiol Infect. (2008) 14:276–8. 10.1111/j.1469-0691.2007.01916.x 18190568

[147] Han X Li Q Shen L Hu D Qu Y. Correlation between the biofilm-forming ability, biofilm-related genes and antimicrobial resistance of *Acinetobacter baumannii*. Zhonghua Wei Zhong Bing Ji Jiu Yi Xue. (2014) 26:639–43. 10.3760/cma.j.issn.2095-4352.2014.09.007 25230865

[148] Zhang D Xia J Xu Y Gong M Zhou Y Xie L. Biological features of biofilm-forming ability of *Acinetobacter baumannii* strains derived from 121 elderly patients with hospital-acquired pneumonia. Clin Exp Med. (2016) 16:73–80. 10.1007/s10238-014-0333-2 25543268

[149] Krzyściak P Chmielarczyk A Pobiega M Romaniszyn D Wójkowska-Mach J. *Acinetobacter baumannii* isolated from hospital-acquired infection: biofilm production and drug susceptibility. APMIS. (2017) 125:1017–26. 10.1111/apm.12739 28913903

[150] Wang YC Huang TW Yang YS Kuo SC Chen CT Liu CP. Biofilm formation is not associated with worse outcome in *Acinetobacter baumannii* bacteraemic pneumonia. Sci Rep. (2018) 8:7289. 10.1038/s41598-018-25661-9 29740176 PMC5940913

[151] Shenkutie AM Yao MZ Siu GK Wong BKC Leung PH. Biofilm-induced antibiotic resistance in clinical *Acinetobacter baumannii* isolates. Antibiotics. (2020) 9:817. 10.3390/antibiotics9110817 33212840 PMC7698371

[152] Donadu MG Mazzarello V Cappuccinelli P Zanetti S Madléna M Nagy ÁL. Relationship between the biofilm-forming capacity and antimicrobial resistance in clinical *Acinetobacter baumannii* isolates: results from a laboratory-based *in vitro* study. Microorganisms. (2021) 9:2384. 10.3390/microorganisms9112384 34835509 PMC8618777

[153] Carattoli A. Plasmids and the spread of resistance. Int J Med Microbiol. (2013) 303:298–304. 10.1016/j.ijmm.2013.02.001 23499304

[154] Wozniak RA Waldor MK. Integrative and conjugative elements: mosaic mobile genetic elements enabling dynamic lateral gene flow. Nat Rev Microbiol. (2010) 8:552–63. 10.1038/nrmicro2382 20601965

[155] Partridge SR Kwong SM Firth N Jensen SO. Mobile genetic elements associated with antimicrobial resistance. Clin Microbiol Rev. (2018) 31:e00088–17. 10.1128/CMR.00088-17 30068738 PMC6148190

[156] Calero-Cáceres W Ye M Balcázar JL. Bacteriophages as environmental reservoirs of antibiotic resistance. Trends Microbiol. (2019) 27:570–7. 10.1016/j.tim.2019.02.008 30905524

[157] Hathroubi S Mekni MA Domenico P Nguyen D Jacques M. Biofilms: microbial shelters against antibiotics. Microb Drug Resist. (2017) 23:147–56. 10.1089/mdr.2016.0087 27214143

[158] Hausner M Wuertz S. High rates of conjugation in bacterial biofilms as determined by quantitative *in situ* analysis. Appl Environ Microbiol. (1999) 65:3710–3. 10.1128/AEM.65.8.3710-3713.1999 10427070 PMC91555

[159] Molin S Tolker-Nielsen T. Gene transfer occurs with enhanced efficiency in biofilms and induces enhanced stabilisation of the biofilm structure. Curr Opin Biotechnol. (2003) 14:255–61. 10.1016/s0958-1669(03)00036-3 12849777

[160] Aminov RI. Horizontal gene exchange in environmental microbiota. Front Microbiol. (2011) 2:158. 10.3389/fmicb.2011.00158 21845185 PMC3145257

[161] Madsen JS Burmølle M Hansen LH Sørensen SJ. The interconnection between biofilm formation and horizontal gene transfer. FEMS Immunol Med Microbiol. (2012) 65:183–95. 10.1111/j.1574-695X.2012.00960.x 22444301

[162] Davey ME O'toole GA. Microbial biofilms: from ecologyto molecular genetics. Microbiol Mol Biol R. (2000) 67:847–67. 10.1128/MMBR.64.4.847-867.2000 11104821 PMC99016

[163] Williams HG Day MJ Fry JC Stewart GJ. Natural transformation in river epilithon. Appl Environ Microbiol. (1996) 62:2994–8. 10.1128/aem.62.8.2994-2998.1996 8702292 PMC168086

[164] McInnes RS McCallum GE Lamberte LE van Schaik W. Horizontal transfer of antibiotic resistance genes in the human gut microbiome. Curr Opin Microbiol. (2020) 53:35–43. 10.1016/j.mib.2020.02.002 32143027

[165] Maeusli M Lee B Miller S Reyna Z Lu P Yan J. Horizontal gene transfer of antibiotic resistance from *Acinetobacter baylyi* to *Escherichia coli* on lettuce and subsequent antibiotic resistance transmission to the gut microbiome. mSphere. (2020) 5:e00329–20. 10.1128/mSphere.00329-20 32461272 PMC7253597

[166] Bridier A Briandet R Thomas V Dubois-Brissonnet F. Resistance of bacterial biofilms to disinfectants: a review. Biofouling. (2011) 27:1017–32. 10.1080/08927014.2011.626899 22011093

[167] Hayashi M Kawamura K Matsui M Suzuki M Suzuki S Shibayama K. Reduction in chlorhexidine efficacy against multi-drug-resistant *Acinetobacter baumannii* international clone II. J Hosp Infect. (2017) 95:318–23. 10.1016/j.jhin.2016.12.004 28159381

[168] Ivanković T Goić-Barišić I Hrenović J. Reduced susceptibility to disinfectants of *Acinetobacter baumannii* biofilms on glass and ceramic. Arh Hig Rada Toksikol. (2017) 68:99–108. 10.1515/aiht-2017-68-2946 30500776

[169] Tipton KA Chin CY Farokhyfar M Weiss DS Rather PN. Role of capsule in resistance to disinfectants, host antimicrobials, and desiccation in *Acinetobacter baumannii*. Antimicrob Agents Chemother. (2018) 62:e01188–18. 10.1128/AAC.01188-18 30297362 PMC6256795

[170] Nor A'shimi MH Alattraqchi AG Mohd Rani F A Rahman NI Ismail S Abdullah FH. Biocide susceptibilities and biofilm-forming capacities of *Acinetobacter baumannii* clinical isolates from Malaysia. J Infect Dev Ctries. (2019) 13:626–33. 10.3855/jidc.11455 32065820

[171] Wassenaar TM Ussery D Nielsen LN Ingmer H. Review and phylogenetic analysis of qac genes that reduce susceptibility to quaternary ammonium compounds in Staphylococcus species. Eur J Microbiol Immunol. (2015) 5:44–61. 10.1556/EUJMI-D-14-00038 25883793 PMC4397847

[172] Zarrilli R. *Acinetobacter baumannii* virulence determinants involved in biofilm growth and adherence to host epithelial cells. Virulence. (2016) 7:367–8. 10.1080/21505594.2016.1150405 26856342 PMC4871643

[173] Donlan RM. Biofilm formation: a clinically relevant microbiological process. Clin Infect Dis. (2001) 33:1387–92. 10.1086/322972 11565080

[174] Djeribi R Bouchloukh W Jouenne T Menaa B. Characterization of bacterial biofilms formed on urinary catheters. Am J Infect Control. (2012) 40:854–9. 10.1016/j.ajic.2011.10.009 22325732

[175] Gil-Perotin S Ramirez P Marti V Sahuquillo JM Gonzalez E Calleja I. Implications of endotracheal tube biofilm in ventilator-associated pneumonia response: a state of concept. Crit Care. (2012) 16:R93. 10.1186/cc11357 22621676 PMC3580639

[176] Ferreira TO Koto RY Leite GF Klautau GB Nigro S Silva CB. Microbial investigation of biofilms recovered from endotracheal tubes using sonication in intensive care unit pediatric patients. Braz J Infect Dis. (2016) 20:468–75. 10.1016/j.bjid.2016.07.003 27513530 PMC9425476

[177] Yeo HJ Yoon SH Lee SE Cho WH Kim D Jeon D. Bacterial biofilms on extracorporeal membrane oxygenation catheters. ASAIO J. (2018) 64:e48–54. 10.1097/MAT.0000000000000750 29356690

[178] Donlan RM. Biofilms and device-associated infections. Emerg Infect Dis. (2001) 7:277–81. 10.3201/eid0702.010226 11294723 PMC2631701

[179] Francolini I Donelli G. Prevention and control of biofilm-based medical-device-related infections. FEMS Immunol Med Microbiol. (2010) 59:227–38. 10.1111/j.1574-695X.2010.00665.x 20412300

[180] Rello J Soñora R Jubert P Artigas A Rué M Vallés J. Pneumonia in intubated patients: role of respiratory airway care. Am J Respir Crit Care Med. (1996) 154:111–5. 10.1164/ajrccm.154.1.8680665 8680665

[181] Raveendra N Rathnakara SH Haswani N Subramaniam V. Bacterial biofilms on tracheostomy tubes. Indian J Otolaryngol Head Neck Surg. (2021) 6:1–5. 10.1007/s12070-021-02598-6 33972925 PMC8100750

[182] Gaynes R Edwards JR National Nosocomial Infections Surveillance System. Overview of nosocomial infections caused by gram-negative bacilli. Clin Infect Dis. (2005) 41:848–54. 10.1086/432803 16107985

[183] Malacarne P Corini M Maremmani P Viaggi B Verdigi S. Diagnostic characteristics of routine surveillance cultures of endotracheal aspirate samples in cases of late-onset ventilator-associated pneumonia due to *Acinetobacter baumannii*. Infect Control Hosp Epidemiol. (2007) 28:867–9. 10.1086/518728 17564991

[184] Bouza E Pérez A Muñoz P Jesús Pérez M Rincón C Sánchez C. Ventilator-associated pneumonia after heart surgery: a prospective analysis and the value of surveillance. Crit Care Med. (2003) 31:1964–70. 10.1097/01.ccm.0000084807.15352.93 12847390

[185] Hayon J Figliolini C Combes A Trouillet JL Kassis N Dombret MC. Role of serial routine microbiologic culture results in the initial management of ventilator-associated pneumonia. Am J Respir Crit Care Med. (2002) 165:41–6. 10.1164/ajrccm.165.1.2105077 11779728

[186] Depuydt P Benoit D Vogelaers D Decruyenaere J Vandijck D Claeys G. Systematic surveillance cultures as a tool to predict involvement of multidrug antibiotic resistant bacteria in ventilator-associated pneumonia. Intensive Care Med. (2008) 34:675–82. 10.1007/s00134-007-0953-z 18066522

[187] Tsakiridou E Makris D Daniil Z Manoulakas E Chatzipantazi V Vlachos O. *Acinetobacter baumannii* infection in prior ICU bed occupants is an independent risk factor for subsequent cases of ventilator-associated pneumonia. Biomed Res Int. (2014) 2014:193516. 10.1155/2014/193516 25101265 PMC4101956

[188] Souza LCD Mota VBRD Carvalho AVDSZ Corrêa RDGCF Libério SA Lopes FF. Association between pathogens from tracheal aspirate and oral biofilm of patients on mechanical ventilation. Braz Oral Res. (2017) 31:e38. 10.1590/1807-3107BOR-2017.vol31.0038 28591237

[189] Natham H Kondagadapu S Kadiyala V Mohan A Chaudhury A Samantaray A. Bacterial colonisation and antibiotic sensitivity profile of endotracheal tubes in mechanically ventilated patients. J Clin Diagn Res. (2019) 13:UC01–6. 10.7860/JCDR/2019/37402.12457

[190] Centers for Disease Control and Prevention. Pneumonia (Ventilator-Associated [VAP] and Nonventilator-Associated Pneumonia [PNEU]) Event. Available online at: https://www.cdc.gov/nhsn/pdfs/pscmanual/6pscvapcurrent.pdf (accessed January 2021).

[191] Ebenezer K James EJ Michael JS Kang G Verghese VP. Ventilator-associated *Acinetobacter baumannii* pneumonia. Indian Pediatr. (2011) 48:964–6. 10.1007/s13312-011-0152-4 21719944

[192] Chen J Yang Y Xiang K Li D Liu H. Combined rifampin and sulbactam therapy for multidrug-resistant acinetobacter baumannii ventilator-associated pneumonia in pediatric patients. J AnesthPerioper Med. (2018) 5:176–85. 10.24015/JAPM.2018.0072 31819924 PMC6901084

[193] Thatrimontrichai A Techato C Dissaneevate S Janjindamai W Maneenil G Kritsaneepaiboon S. Risk factors and outcomes of carbapenem-resistant *Acinetobacter baumannii* ventilator-associated pneumonia in the neonate: a case-case-control study. J Infect Chemother. (2016) 22:444–9. 10.1016/j.jiac.2016.03.013 27229539

[194] Iosifidis E Pitsava G Roilides E. Ventilator-associated pneumonia in neonates and children: a systematic analysis of diagnostic methods and prevention. Future Microbiol. (2018) 13:1431–46. 10.2217/fmb-2018-0108 30256161

[195] Asadian M Azimi L Alinejad F Ostadi Y Lari AR. Molecular characterization of *Acinetobacter baumannii* isolated from ventilator-associated pneumonia and burn wound colonization by random amplified polymorphic DNA polymerase chain reaction and the relationship between antibiotic susceptibility and biofilm production. Adv Biomed Res. (2019) 8:58. 10.4103/abr.abr_256_18 31673531 PMC6777144

[196] Kara SS Polat M Tapisiz A Kalkan G Simsek H Tezer H. Ventilator associated pneumonia due to carbapenem resistant microorganisms in children. Minerva Pediatr. (2019) 71:349–57. 10.23736/S0026-4946.17.04284-0 31268280

[197] Gominet M Compain F Beloin C Lebeaux D. Central venous catheters and biofilms: where do we stand in 2017? APMIS. (2017) 125:365–75. 10.1111/apm.12665 28407421

[198] Storti A Pizzolitto AC Pizzolitto EL. Detection of mixed microbial biofilms on central venous catheters removed from intensive care unit patients. Braz J Microbiol. (2005) 36:275–80. 10.1590/S1517-83822005000300013

[199] Basher H Farhana A Alim S Khan S. Detection of catheter related blood stream infections in an ICU of a tertiary care center in Northern India. IJMR. (2021) 8:102–7. 10.18231/j.ijmr.2021.020

[200] Yoon EJ Kim D Lee H Lee HS Shin JH Uh Y. Counter clinical prognoses of patients with bloodstream infections between causative *Acinetobacter baumannii* clones ST191 and ST451 belonging to the international clonal lineage II. Front Public Health. (2019) 7:233. 10.3389/fpubh.2019.00233 31475131 PMC6707333

[201] Yu K Zeng W Xu Y Liao W Xu W Zhou T. Bloodstream infections caused by ST2 *Acinetobacter baumannii*: risk factors, antibiotic regimens, and virulence over 6 years period in China. Antimicrob Resist Infect Control. (2021) 10:16. 10.1186/s13756-020-00876-6 33461617 PMC7814448

[202] Downes KJ Metlay JP Bell LM McGowan KL Elliott MR Shah SS. Polymicrobial bloodstream infections among children and adolescents with central venous catheters evaluated in ambulatory care. Clin Infect Dis. (2008) 46:387–94. 10.1086/525265 18181737

[203] Ozdemir H Kendirli T Ergün H Ciftçi E Tapisiz A Güriz H. Nosocomial infections due to *Acinetobacter baumannii* in a pediatric intensive care unit in Turkey. Turk J Pediatr. (2011) 53:255–60. 21980805

[204] Thomas D Parameswaran N Harish BN. Catheter related blood stream infections in the paediatric intensive care unit: a descriptive study. Indian J Crit Care Med. (2013) 17:135–9. 10.4103/0972-5229.117038 24082609 PMC3777366

[205] Nicolle LE. Catheter-related urinary tract infection. Drugs Aging. (2005) 22:627–39. 10.2165/00002512-200522080-00001 16060714

[206] Gilmore BF Carson L. Bioactive biomaterials for controlling biofilms. In: Barnes L Cooper I, editors. Biomaterials and Medical Device-Associated Infections. 1st ed., Chapter 8. Woodhead (2014). p. 281.

[207] Morris FC Dexter C Kostoulias X Uddin MI Peleg AY. The mechanisms of disease caused by *Acinetobacter baumannii*. Front Microbiol. (2019) 10:1601. 10.3389/fmicb.2019.01601 31379771 PMC6650576

[208] De Francesco MA Ravizzola G Peroni L Negrini R Manca N. Urinary tract infections in Brescia, Italy: etiology of uropathogens and antimicrobial resistance of common uropathogens. Med Sci Monit. (2007) 13:BR136–44. 17534228

[209] Sievert DM Ricks P Edwards JR Schneider A Patel J Srinivasan A. Antimicrobial-resistant pathogens associated with healthcare-associated infections: summary of data reported to the National Healthcare Safety Network at the Centers for Disease Control and Prevention, 2009-2010. Infect Control Hosp Epidemiol. (2013) 34:1–14. 10.1086/668770 23221186

[210] Alves MJ Barreira JCM Carvalho I Trinta L Perreira L Ferreira ICFR. Propensity for biofilm formation by clinical isolates from urinary tract infections: developing a multifactorial predictive model to improve antibiotherapy. J Med Microbiol. (2014) 63:471–7. 10.1099/jmm.0.071746-0 24430252

[211] Maharjan G Khadka P Siddhi SG Chapagain G Dhungana GR. Catheter-associated urinary tract infection and obstinate biofilm producers. Can J Infect Dis Med Microbiol. (2018) 2018:7624857. 10.1155/2018/7624857 30224941 PMC6129315

[212] Bagińska N Cieślik M Górski A Jończyk-Matysiak E. The role of antibiotic resistant *A. baumannii* in the pathogenesis of urinary tract infection and the potential of its treatment with the use of bacteriophage therapy. Antibiotics. (2021) 10:281. 10.3390/antibiotics10030281 33803438 PMC8001842

[213] Mashouf RY Babalhavaeji H Yousef J. Urinary tract infections: bacteriology and antibiotic resistance patterns. Indian Pediatr. (2009) 46:617–20. 19430071

[214] Madhu GN AnjumAara CA Mahamud S. Prevalence and antibiotic susceptibility pattern of pathogens in children with urinary tract infection in a tertiary care hospital. Int J Contemporary Pediatrics. (2020) 7:1513–8. 10.18203/2349-3291.ijcp20202607

[215] Srinivasan R Santhakumari S Poonguzhali P Geetha M Dyavaiah M Xiangmin L. Bacterial biofilm inhibition: a focused review on recent therapeutic strategies for combating the biofilm mediated infections. Front Microbiol. (2021) 12:676458. 10.3389/fmicb.2021.676458 34054785 PMC8149761

[216] Bjarnsholt T Kirketerp-Møller K Jensen PØ Madsen KG Phipps R Krogfelt K. Why chronic wounds will not heal: a novel hypothesis. Wound Repair Regen. (2008) 16:2–10. 10.1111/j.1524-475X.2007.00283.x 18211573

[217] Davis KA Moran KA McAllister CK Gray PJ. Multidrug-resistant Acinetobacter extremity infections in soldiers. Emerg Infect Dis. (2005) 11:1218–24. 10.3201/1108.050103 16102310 PMC3320488

[218] Oncül O Keskin O Acar HV Küçükardali Y Evrenkaya R Atasoyu EM. Hospital-acquired infections following the 1999 Marmara earthquake. J Hosp Infect. (2002) 51:47–51. 10.1053/jhin.2002.1205 12009820

[219] Kennedy PJ Haertsch PA Maitz PK. The Bali burn disaster: implications and lessons learned. J Burn Care Rehabil. (2005) 26:125–31. 10.1097/01.bcr.0000155532.31639.0d 15756113

[220] James GA Swogger E Wolcott R Pulcini Ed Secor P Sestrich J. Biofilms in chronic wounds. Wound Repair Regen. (2008) 16:37–44. 10.1111/j.1524-475X.2007.00321.x 18086294

[221] Sheppard FR Keiser P Craft DW Gage F Robson M Brown TS. The majority of US combat casualty soft-tissue wounds are not infected or colonized upon arrival or during treatment at a continental US military medical facility. Am J Surg. (2010) 200:489–95. 10.1016/j.amjsurg.2010.03.001 20887842

[222] Nair MS Lau P Liu Y Venkitanarayanan K. Efficacy of selenium in controlling *Acinetobacter baumannii* associated wound infections. Wound Med. (2019) 26:100165. 10.1016/j.wndm.2019.100165

[223] Williams DL Kawaguchi B Taylor NB Allyn G Badham MA Rogers JC. In vivo efficacy of a unique first-in-class antibiofilm antibiotic for biofilm-related wound infections caused by *Acinetobacter baumannii. Biofilm*. (2020) 2:100032. 10.1016/j.bioflm.2020.100032 33447817 PMC7798455

[224] Fekrirad Z Darabpour E Kashef N. Eradication of *Acinetobacter baumannii* planktonic and biofilm cells through erythrosine-mediated photodynamic inactivation augmented by acetic acid and chitosan. Curr Microbiol. (2021) 78:879–86. 10.1007/s00284-021-02350-x 33512576 PMC7845581

[225] Sebeny PJ Riddle MS Petersen K. *Acinetobacter baumannii* skin and soft-tissue infection associated with war trauma. Clin Infect Dis. (2008) 47:444–9. 10.1086/590568 18611157

[226] Guerrero DM Perez F Conger NG Solomkin JS Adams MD Rather PN. *Acinetobacter baumannii* -associated skin and soft tissue infections: recognizing a broadening spectrum of disease. Surg Infect. (2010) 11:49–57. 10.1089/sur.2009.022 19788383 PMC2956563

[227] Sinha N Niazi M Lvovsky D. A fatal case of multidrug resistant acinetobacter necrotizing fasciitis: the changing scary face of nosocomial infection. Case Rep Infect Dis. (2014) 2014:705279. 10.1155/2014/705279 25349748 PMC4202280

[228] Ali A Botha J Tiruvoipati R. Fatal skin and soft tissue infection of multidrug resistant *Acinetobacter baumannii*: a case report. Int J Surg Case Rep. (2014) 5:532–6. 10.1016/j.ijscr.2014.04.019 25016080 PMC4147652

[229] Campoccia D Montanaro L Arciola CR. The significance of infection related to orthopedic devices and issues of antibiotic resistance. Biomaterials. (2006) 27:2331–9. 10.1016/j.biomaterials.2005.11.044 16364434

[230] Zimmerli W Moser C. Pathogenesis and treatment concepts of orthopaedic biofilm infections. FEMS Immunol Med Microbiol. (2012) 65:158–68. 10.1111/j.1574-695X.2012.00938.x 22309166

[231] Staats A Li D Sullivan AC Stoodley P. Biofilm formation in periprosthetic joint infections. Ann Joint. (2020) 6:43. 10.21037/aoj-20-85 34859164 PMC8635410

[232] Gramatniece A Silamikelis I Zahare I Urtans V Zahare I Dimina E. Control of *Acinetobacter baumannii* outbreak in the neonatal intensive care unit in Latvia: whole-genome sequencing powered investigation and closure of the ward. Antimicrob Resist Infect Control. (2019) 22:8:84. 10.1186/s13756-019-0537-z 31143444 PMC6532256

[233] Duenas M. Outbreak of multidrug-resistant *Acinetobacter baumannii* in neonatal intensive care unit in El salvador. Open Forum Infectious Diseases. (2015) 2:1778. 10.1093/ofid/ofv133.1328

[234] Dalili H Nayeri F Shariat M Farrokhzad N Sahebi L. The outbreak of *Acinetobacter baumannii* over a 19-month period in a teaching hospital Neonatal intensive care unit. Clin Pediatr OA. (2019) 4:150. 10.35248/2572-0775.19.4.150

[235] Chatterjee S Datta S Roy S Ramanan L Saha A Viswanathan R. Carbapenem resistance in *Acinetobacter baumannii* and other *Acinetobacter spp*. causing neonatal sepsis: focus on NDM-1 and its linkage to ISAba125. Front Microbiol. (2016) 7:1126. 10.3389/fmicb.2016.01126 27551277 PMC4976090

[236] Roy S Basu S Dasgupta S Singh AK Viswanathan R. Carbapenem resistance in *Acinetobacter baumannii* isolated from blood of neonates with sepsis. Indian J Med Microbiol. (2010) 28:416–7. 10.4103/0255-0857.71814 20966591

[237] Lee SY Lee JW Jeong DC Chung SY Chung DS Kang JH. Multidrug-resistant Acinetobacter meningitis in a 3-year-old boy treated with i.v. colistin. Pediatr Int. (2008) 50:584–5. 10.1111/j.1442-200X.2008.02677.x 18937759

[238] Ozaki T Nishimura N Arakawa Y Suzuki M Narita A Yamamoto Y. Community-acquired *Acinetobacter baumannii* meningitis in a previously healthy 14-month-old boy. J Infect Chemother. (2009) 15:322–4. 10.1007/s10156-009-0704-x 19856071

[239] Shah I Kapdi M. Multidrug-resistant *Acinetobacter* meningitis in children. J Family Med Prim Care. (2016) 5:858–9. 10.4103/2249-4863.201169 28349005 PMC5353828

[240] Xiao J Zhang C Ye S. *Acinetobacter baumannii* meningitis in children: a case series and literature review. Infection. (2019) 47:643–9. 10.1007/s15010-018-1234-1 30328074

[241] Ozdemir H Tapisiz A Ciftçi E Ince E Mokhtari H Güriz H. Successful treatment of three children with post-neurosurgical multidrug-resistant *Acinetobacter baumannii* meningitis. Infection. (2010) 38:241–4. 10.1007/s15010-010-0018-z 20358244

[242] Clark R Powers R White R Bloom B Sanchez P Benjamin DK Jr. Nosocomial infection in the NICU: a medical complication or unavoidable problem? J Perinatol. (2004) 24:382–8. 10.1038/sj.jp.7211120 15116140

[243] Roy S Viswanathan R Singh A Das P Basu S. Gut colonization by multidrug-resistant and carbapenem-resistant *Acinetobacter baumannii* in neonates. Eur J Clin Microbiol Infect Dis. (2010) 29:1495–500. 10.1007/s10096-010-1030-z 20730467

[244] Investigators of the Delhi Neonatal Infection Study (DeNIS) collaboration. Characterisation and antimicrobial resistance of sepsis pathogens in neonates born in tertiary care centres in Delhi, India: a cohort study. Lancet Glob Health. (2016) 4:e752–60. 10.1016/S2214-109X(16)30148-6 27633433

[245] Lawrence RM. Transmission of infectious diseases through breast milk and breastfeeding. Breastfeeding. (2011) 406–73. 10.1016/B978-1-4377-0788-5.10013-6

[246] Ciofu O Rojo-Molinero E Macià MD Oliver A. Antibiotic treatment of biofilm infections. APMIS. (2017) 125:304–19. 10.1111/apm.12673 28407419

[247] Wang Y Bao W Guo N Chen H Cheng W Jin K. Antimicrobial activity of the imipenem/rifampicin combination against clinical isolates of *Acinetobacter baumannii* grown in planktonic and biofilm cultures. World J Microbiol Biotechnol. (2014) 30:3015–25. 10.1007/s11274-014-1728-7 25298216

[248] Song JY Cheong HJ Noh JY Kim WJ. *In vitro* comparison of anti-biofilm effects against carbapenem-resistant *Acinetobacter baumannii*: imipenem, colistin, tigecycline, rifampicin and combinations. Infect Chemother. (2015) 47:27–32. 10.3947/ic.2015.47.1.27 25844260 PMC4384457

[249] Wang YC Kuo SC Yang YS Lee YT Chiu CH Chuang MF. Individual or combined effects of meropenem, imipenem, sulbactam, colistin, and tigecycline on biofilm-embedded *Acinetobacter baumannii* and biofilm architecture. Antimicrob Agents Chemother. (2016) 60:4670–6. 10.1128/AAC.00551-16 27216052 PMC4958180

[250] Peng Q Lin F Ling B. In vitro activity of biofilm inhibitors in combination with antibacterial drugs against extensively drug-resistant *Acinetobacter baumannii. Sci Rep*. (2020) 10:18097. 10.1038/s41598-020-75218-y 33093606 PMC7581519

[251] Ozbek B Mataraci E. *In vitro* effectiveness of colistin, tigecycline and levofloxacin alone and combined with clarithromycin and/or heparin as lock solutions against embedded *Acinetobacter baumannii* strains. J Antimicrob Chemother. (2013) 68:827–30. 10.1093/jac/dks472 23203948

[252] Cho YW Park JH Kim SH Cho YH Choi JM Shin HJ. Gentamicin-releasing urethral catheter for short-term catheterization. J Biomater Sci Polym Ed. (2003) 14:963–72. 10.1163/156856203322381447 14661873

[253] Darouiche RO Smith JA Jr Hanna H Dhabuwala CB Steiner MS Babaian RJ. Efficacy of antimicrobial-impregnated bladder catheters in reducing catheter-associated bacteriuria: a prospective, randomized, multicenter clinical trial. Urology. (1999) 54:976–81. 10.1016/s0090-4295(99)00288-5 10604693

[254] Park JH Cho YW Cho YH Choi JM Shin HJ Bae YH. Norfloxacin-releasing urethral catheter for long-term catheterization. J Biomater Sci Polym Ed. (2003) 14:951–62. 10.1163/156856203322381438 14661872

[255] León C Ruiz-Santana S Rello J de la Torre MV Vallés J Alvarez-Lerma F. Benefits of minocycline and rifampin-impregnated central venous catheters. A prospective, randomized, double-blind, controlled, multicenter trial. Intensive Care Med. (2004) 30:1891–9. 10.1007/s00134-004-2378-2 15278273

[256] Rupp ME Lisco SJ Lipsett PA Perl TM Keating K Civetta JM. Effect of a second-generation venous catheter impregnated with chlorhexidine and silver sulfadiazine on central catheter-related infections: a randomized, controlled trial. Ann Intern Med. (2005) 143:570–80. 10.7326/0003-4819-143-8-200510180-00007 16230723

[257] Yücel N Lefering R Maegele M Max M Rossaint R Koch A. Reduced colonization and infection with miconazole-rifampicin modified central venous catheters: a randomized controlled clinical trial. J Antimicrob Chemother. (2004) 54:1109–15. 10.1093/jac/dkh483 15537696

[258] Xiong YQ Estellés A Li L Abdelhady W Gonzales R Bayer AS. A human biofilm-disrupting monoclonal antibody potentiates antibiotic efficacy in rodent models of both *Staphylococcus aureus* and *Acinetobacter baumannii* infections. Antimicrob Agents Chemother. (2017) 61:e00904–17. 10.1128/AAC.00904-17 28717038 PMC5610488

[259] Sperandio V. Novel approaches to bacterial infection therapy by interfering with bacteria-to-bacteria signaling. Expert Rev Anti Infect Ther. (2007) 5:271–6. 10.1586/14787210.5.2.271 17402841 PMC2613682

[260] Hoang TT Schweizer HP. Characterization of *Pseudomonas aeruginosa* enoyl-acyl carrier protein reductase (FabI): a target for the antimicrobial triclosan and its role in acylated homoserine lactone synthesis. J Bacteriol. (1999) 181:5489–97. 10.1128/JB.181.17.5489-5497.1999 10464225 PMC94060

[261] Choo JH Rukayadi Y Hwang JK. Inhibition of bacterial quorum sensing by vanilla extract. Lett Appl Microbiol. (2006) 42:637–41. 10.1111/j.1472-765X.2006.01928.x 16706905

[262] Rasmussen TB Bjarnsholt T Skindersoe ME Hentzer M Kristoffersen P Köte M. Screening for quorum-sensing inhibitors (QSI) by use of a novel genetic system, the QSI selector. J Bacteriol. (2005) 187:1799–814. 10.1128/JB.187.5.1799-1814.2005 15716452 PMC1063990

[263] Cady NC McKean KA Behnke J Kubec R Mosier AP Kasper SH. Inhibition of biofilm formation, quorum sensing and infection in *Pseudomonas aeruginosa* by natural products-inspired organosulfur compounds. PLoS ONE. (2012) 7:e38492. 10.1371/journal.pone.0038492 22715388 PMC3371053

[264] Stacy DM Welsh MA Rather PN Blackwell HE. Attenuation of quorum sensing in the pathogen *Acinetobacter baumannii* using non-native N-Acyl homoserine lactones. ACS Chem Biol. (2012) 7:1719–28. 10.1021/cb300351x 22853441 PMC3477293

[265] Sambanthamoorthy K Luo C Pattabiraman N Feng X Koestler B Waters CM. Identification of small molecules inhibiting diguanylate cyclases to control bacterial biofilm development. Biofouling. (2014) 30:17–28. 10.1080/08927014.2013.832224 24117391 PMC4120261

[266] Young DM Parke D Ornston LN. Opportunities for genetic investigation afforded by *Acinetobacter baylyi*, a nutritionally versatile bacterial species that is highly competent for natural transformation. Annu Rev Microbiol. (2005) 59:519–51. 10.1146/annurev.micro.59.051905.105823 16153178

[267] Nait Chabane Y Mlouka MB Alexandre S Nicol M Marti S Pestel-Caron M. Virstatin inhibits biofilm formation and motility of *Acinetobacter baumannii*. BMC Microbiol. (2014) 14:62. 10.1186/1471-2180-14-62 24621315 PMC4007623

[268] Oh MH Choi CH. Role of LuxIR homologue AnoIR in acinetobacter nosocomialis and the effect of virstatin on the expression of anoR gene. J Microbiol Biotechnol. (2015) 25:1390–400. 10.4014/jmb.1504.04069 25975610

[269] Alam P Alqahtani AS Mabood HF Tabish RM Alajmi MF Noman OM. Siphonocholin isolated from red sea sponge *Siphonochalina siphonella* attenuates quorum sensing controlled virulence and biofilm formation. Saudi Pharm J. (2020) 28:1383–91. 10.1016/j.jsps.2020.09.002 33250645 PMC7679466

[270] Chow JY Yang Y Tay SB Chua KL Yew WS. Disruption of biofilm formation by the human pathogen *Acinetobacter baumannii* using engineered quorum-quenching lactonases. Antimicrob Agents Chemother. (2014) 58:1802–5. 10.1128/AAC.02410-13 24379199 PMC3957888

[271] Zhang Y Brackman G Coenye T. Pitfalls associated with evaluating enzymatic quorum quenching activity: the case of MomL and its effect on *Pseudomonas aeruginosa* and *Acinetobacter baumannii* biofilms. PeerJ. (2017) 5:e3251. 10.7717/peerj.3251 28462048 PMC5410158

[272] Paluch E Rewak-Soroczyńska J Jedrusik I Mazurkiewicz E Jermakow K. Prevention of biofilm formation by quorum quenching. Appl Microbiol Biotechnol. (2020) 104:1871–81. 10.1007/s00253-020-10349-w 31927762 PMC7007913

[273] Seo H Kim J Jung J Jin HM Jeon CO Park W. Complexity of cell-cell interactions between *Pseudomonas* sp. AS1 and Acinetobacter oleivorans DR1: metabolic commensalism, biofilm formation and quorum quenching. Res Microbiol. (2012) 163:173–81. 10.1016/j.resmic.2011.12.003 22202171

[274] Simões LC Simões M Vieira MJ. Intergeneric coaggregation among drinking water bacteria: evidence of a role for Acinetobacter calcoaceticus as a bridging bacterium. Appl Environ Microbiol. (2008) 74:1259–63. 10.1128/AEM.01747-07 18156333 PMC2258588

[275] Zhang J Liang X Zhang S Song Z Wang C Xu Y. Maipomycin A, a novel natural compound with promising anti-biofilm activity against gram-negative pathogenic bacteria. Front Microbiol. (2021) 11:598024. 10.3389/fmicb.2020.598024 33510721 PMC7835661

[276] Stowe SD Richards JJ Tucker AT Thompson R Melander C Cavanagh J. Anti-biofilm compounds derived from marine sponges. Mar Drugs. (2011) 9:2010–35. 10.3390/md9102010 22073007 PMC3210616

[277] Tseng SP Hung WC Huang CY Lin YS Chan MY Lu PL. 5-Episinuleptolide decreases the expression of the extracellular matrix in early biofilm formation of multi-drug resistant *Acinetobacter baumannii*. Mar Drugs. (2016) 14:143. 10.3390/md14080143 27483290 PMC4999904

[278] King LB Pangburn MK McDaniel LS. Serine protease PKF of *Acinetobacter baumannii* results in serum resistance and suppression of biofilm formation. J Infect Dis. (2013) 207:1128–34. 10.1093/infdis/jis939 23303803

[279] Selvaraj A Valliammai A Sivasankar C Suba M Sakthivel G Pandian SK. Antibiofilm and antivirulence efficacy of myrtenol enhances the antibiotic susceptibility of *Acinetobacter baumannii*. Sci Rep. (2020) 10:21975. 10.1038/s41598-020-79128-x 33319862 PMC7738676

[280] Nazzaro F Fratianni F De Martino L Coppola R De Feo V. Effect of essential oils on pathogenic bacteria. Pharmaceuticals. (2013) 6:1451–74. 10.3390/ph6121451 24287491 PMC3873673

[281] Ugur T Cem C Isa K Ataş Ceylan MH. Anti-biofilm and antimicrobial activity of *Mentha pulegium* L essential oil against multidrug-resistant *Acinetobacter baumannii*. Trop J Pharm Res. (2016) 5:1039–47. 10.4314/tjpr.v15i5.20

[282] Celik C Tutar U Karaman I Hepokur C Atas M. Evaluation of the antibiofilm and antimicrobial properties of *Ziziphora tenuior* L. essential oil against multidrug-resistant *Acinetobacter baumannii*. Int J Pharm. (2016) 12:28–35. 10.3923/ijp.2016.28.35

[283] Orhan-Yanikan E da Silva-Janeiro S Ruiz-Rico M Jimenez-Belenguer A Ayhan K Bharat JM. Essential oils compounds as antimicrobial and antibiofilm agents against strains present in the meat industry. Food Control. (2019) 101:29–38. 10.1016/j.foodcont.2019.02.035

[284] Mayaud L Carricajo A Zhiri A Aubert G. Comparison of bacteriostatic and bactericidal activity of 13 essential oils against strains with varying sensitivity to antibiotics. Lett Appl Microbiol. (2008) 47:167–73. 10.1111/j.1472-765X.2008.02406.x 19552780

[285] Knezevic P Aleksic V Simin N Svircev E Petrovic A Mimica-Dukic N. Antimicrobial activity of *Eucalyptus camaldulensis* essential oils and their interactions with conventional antimicrobial agents against multi-drug resistant *Acinetobacter baumannii*. J Ethnopharmacol. (2016) 178:125–36. 10.1016/j.jep.2015.12.008 26671210

[286] Moghimi R Aliahmadi A Rafati H Abtahi HR Amini S Feizabadi MM. Antibacterialand anti-biofilm activity of nanoemulsion of *Thymus daenensis* oil against multi-drug resistant *Acinetobacter baumannii*. J Mol Liq. (2018) 265:765–70. 10.1016/j.molliq.2018.07.023

[287] Shivaprasad A Antony B Rekha B. Antibiofilm activity and the synergistic effect of lemon grass essential oil with antibiotics against *A. baumannii* complex – a preliminary report. EJPMR. (2019) 6:505–10.

[288] Ismail MM Samir R Saber FR Ahmed SR Farag MA. *Pimenta* oil as a potential treatment for *Acinetobacter Baumannii* wound infection: *in vitro* and *in vivo* bioassays in relation to its chemical composition. Antibiotics. (2020) 9:679. 10.3390/antibiotics9100679 33036456 PMC7600634

[289] Lu M Dai T Murray CK Wu MX. Bactericidal property of oregano oil against multidrug-resistant clinical isolates. Front Microbiol. (2018) 9:2329. 10.3389/fmicb.2018.02329 Erratum in: *Front Microbiol*. (2021) 12:713573. 30344513 PMC6182053

[290] Fjell CD Hiss JA Hancock RE Schneider G. Designing antimicrobial peptides: form follows function. Nat Rev Drug Discov. (2011) 11:37–51. 10.1038/nrd3591 22173434

[291] Feng X Sambanthamoorthy K Palys T Paranavitana C. The human antimicrobial peptide LL-37 and its fragments possess both antimicrobial and antibiofilm activities against multidrug-resistant *Acinetobacter baumannii*. Peptides. (2013) 49:131–7. 10.1016/j.peptides.2013.09.007 24071034

[292] Avery TM Boone RL Lu J Spicer SK Guevara MA Moore RE. Analysis of antimicrobial and antibiofilm activity of human milk lactoferrin compared to bovine lactoferrin against multidrug resistant and susceptible *Acinetobacter baumannii* clinical isolates. ACS Infect Dis. (2021) 7:2116–26. 10.1021/acsinfecdis.1c00087 34105954 PMC8577213

[293] Mohamed MF Brezden A Mohammad H Chmielewski J Seleem MN. A short D-enantiomeric antimicrobial peptide with potent immunomodulatory and antibiofilm activity against multidrug-resistant *Pseudomonas aeruginosa* and *Acinetobacter baumannii*. Sci Rep. (2017) 7:6953. 10.1038/s41598-017-07440-0 28761101 PMC5537347

[294] Kim MK Kang N Ko SJ Park J Park E Shin DW. Antibacterial and antibiofilm activity and mode of action of magainin 2 against drug-resistant *Acinetobacter baumannii*. Int J Mol Sci. (2018) 19:3041. 10.3390/ijms19103041 30301180 PMC6213043

[295] Peng J Long H Liu W Wu Z Wang T Zeng Z. Antibacterial mechanism of peptide Cec4 against *Acinetobacter baumannii*. Infect Drug Resist. (2019) 12:2417–28. 10.2147/IDR.S214057 31496754 PMC6689099

[296] Gordya N Yakovlev A Kruglikova A Tulin D Potolitsina E Suborova T. Natural antimicrobial peptide complexes in the fighting of antibiotic resistant biofilms: *Calliphora vicina* medicinal maggots. PLoS ONE. (2017) 12:e0173559. 10.1371/journal.pone.0173559 28278280 PMC5344439

[297] Jaśkiewicz M Neubauer D Kazor K Bartoszewska S Kamysz W. Antimicrobial activity of selected antimicrobial peptides against planktonic culture and biofilm of *Acinetobacter baumannii*. Probiotics Antimicrob Proteins. (2019) 11:317–24. 10.1007/s12602-018-9444-5 30043322 PMC6449538

[298] Gopal R Kim YG Lee JH Lee SK Chae JD Son BK. Synergistic effects and antibiofilm properties of chimeric peptides against multidrug-resistant *Acinetobacter baumannii* strains. Antimicrob Agents Chemother. (2014) 58:1622–9. 10.1128/AAC.02473-13 24366740 PMC3957903

[299] Swedan S Shubair Z Almaaytah A. Synergism of cationic antimicrobial peptide WLBU2 with antibacterial agents against biofilms of multi-drug resistant *Acinetobacter baumannii* and *Klebsiella pneumoniae*. Infect Drug Resist. (2019) 12:2019–30. 10.2147/IDR.S215084 31372010 PMC6636432

[300] Mwangi J Yin Y Wang G Yang M Li Y Zhang Z. The antimicrobial peptide ZY4 combats multidrug-resistant *Pseudomonas aeruginosa* and *Acinetobacter baumannii* infection. Proc Natl Acad Sci USA. (2019) 116:26516–22. 10.1073/pnas.1909585117 31843919 PMC6936460

[301] de Breij A Riool M Cordfunke RA Malanovic N de Boer L Koning RI. The antimicrobial peptide SAAP-148 combats drug-resistant bacteria and biofilms. Sci Transl Med. (2018) 10:eaan4044. 10.1126/scitranslmed.aan4044 29321257

[302] Di Somma A Recupido F Cirillo A Romano A Romanelli A Caserta S. Antibiofilm properties of temporin-L on *Pseudomonas fluorescens* in static and in-flow conditions. Int J Mol Sci. (2020) 21:8526. 10.3390/ijms21228526 33198325 PMC7696879

[303] Reza A Sutton JM Rahman KM. Effectiveness of efflux pump inhibitors as biofilm disruptors and resistance breakers in gram-negative (ESKAPEE) bacteria. Antibiotics. (2019) 8:229. 10.3390/antibiotics8040229 31752382 PMC6963839

[304] Chen L Li H Wen H Zhao B Niu Y Mo Q. Biofilm formation in *Acinetobacter baumannii* was inhibited by PAβN while it had no association with antibiotic resistance. Microbiologyopen. (2020) 9:e1063. 10.1002/mbo3.1063 32700454 PMC7520992

[305] Blanchard C Barnett P Perlmutter J Dunman PM. Identification of *Acinetobacter baumannii* serum-associated antibiotic efflux pump inhibitors. Antimicrob Agents Chemother. (2014) 58:6360–70. 10.1128/AAC.03535-14 25114126 PMC4249429

[306] Yilmaz S Altinkanat-Gelmez G Bolelli K Guneser-Merdan D Over-Hasdemir MU Yildiz I. Pharmacophore generation of 2-substituted benzothiazoles as AdeABC efflux pump inhibitors in *A. baumannii*. SAR QSAR Environ Res. (2014) 25:551–63. 10.1080/1062936X.2014.919357 24905472

[307] Krishnamoorthy S Shah BP Lee HH Martinez LR. Microbicides alter the expression and function of RND-type efflux pump AdeABC in biofilm-associated cells of *Acinetobacter baumannii* clinical isolates. Antimicrob Agents Chemother. (2015) 60:57–63. 10.1128/AAC.01045-15 26459900 PMC4704159

[308] Wang L Hu C Shao L. The antimicrobial activity of nanoparticles: present situation and prospects for the future. Int J Nanomedicine. (2017) 12:1227–49. 10.2147/IJN.S121956 28243086 PMC5317269

[309] Mihu MR Sandkovsky U Han G Friedman JM Nosanchuk JD Martinez LR. The use of nitric oxide releasing nanoparticles as a treatment against *Acinetobacter baumannii* in wound infections. Virulence. (2010) 1:62–7. 10.4161/viru.1.2.10038 21178416

[310] Hwang YY Ramalingam K Bienek DR Lee V You T Alvarez R. Antimicrobial activity of nanoemulsion in combination with cetylpyridinium chloride in multidrug-resistant *Acinetobacter baumannii*. Antimicrob Agents Chemother. (2013) 57:3568–75. 10.1128/AAC.02109-12 23669390 PMC3719747

[311] Salunke GR Ghosh S Santosh Kumar RJ Khade S Vashisth P Kale T. Rapid efficient synthesis and characterization of silver, gold, and bimetallic nanoparticles from the medicinal plant Plumbago zeylanica and their application in biofilm control. Int J Nanomedicine. (2014) 9:2635–53. 10.2147/IJN.S59834 24920901 PMC4043712

[312] Gaidhani S Singh R Singh D Patel U Shevade K Yeshvekar R. Biofilm disruptionactivity of silver nanoparticles synthesized by Acinetobacter calcoaceticus PUCM 1005. Mater Lett. (2013) 108:324–7. 10.1016/j.matlet.2013.07.023

[313] Martinez-Gutierrez F Boegli L Agostinho A Sánchez EM Bach H Ruiz F. Anti-biofilm activity of silver nanoparticles against different microorganisms. Biofouling. (2013) 29:651–60. 10.1080/08927014.2013.794225 23731460

[314] Ramya S Shanmugasundaram T Balagurunathan R. Biomedical potential of actinobacterially synthesized selenium nanoparticles with special reference to anti-biofilm, anti-oxidant, wound healing, cytotoxic and anti-viral activities. J Trace Elem Med Biol. (2015) 32:30–9. 10.1016/j.jtemb.2015.05.005 26302909

[315] Hosseini A Nejadsattari T Zargar M. *In vitro* anti-biofilm activity of curcumin nanoparticles in *Acinetobacter baumannii*: a culture-based and molecular approach. Arch Clin Infect Dis. (2019) 14:e83263. 10.5812/archcid.83263

[316] Muzammil S Khurshid M Nawaz I Siddique MH Zubair M Nisar MA. Aluminium oxide nanoparticles inhibit EPS production, adhesion and biofilm formation by multidrug resistant *Acinetobacter baumannii*. Biofouling. (2020) 36:492–504. 10.1080/08927014.2020.1776856 32529892

[317] Hendiani S Abdi AA Mohammadi P Kharrazi Sh. Synthesis of silver nanoparticles and its synergistic effects in combination with imipenem and two biocides against biofilm producing *Acinetobacter baumannii*. Nanomed J. (2015) 2:291–8. 10.7508/NMJ.2015.04.007

[318] Tran HM Tran H Booth MA Fox KE Nguyen TH Tran N. Nanomaterials for treating bacterial biofilms on implantable medical devices. Nanomaterials. (2020) 10:2253. 10.3390/nano10112253 33203046 PMC7696307

[319] Thawal ND Yele AB Sahu PK Chopade BA. Effect of a novel podophage AB7-IBB2 on *Acinetobacter baumannii* biofilm. Curr Microbiol. (2012) 65:66–72. 10.1007/s00284-012-0127-2 22535475

[320] Yele AB Thawal ND Sahu PK Chopade BA. Novel lytic bacteriophage AB7-IBB1 of *Acinetobacter baumannii*: isolation, characterization and its effect on biofilm. Arch Virol. (2012) 157:1441–50. 10.1007/s00705-012-1320-0 22552486

[321] Liu Y Mi Z Niu W An X Yuan X Liu H. Potential of a lytic bacteriophage to disrupt *Acinetobacter baumannii* biofilms *in vitro*. Future Microbiol. (2016) 11:1383–93. 10.2217/fmb-2016-0104 27538011

[322] Lood R Winer BY Pelzek AJ Diez-Martinez R Thandar M Euler CW. Novel phage lysin capable of killing the multidrug-resistant gram-negative bacterium *Acinetobacter baumannii* in a mouse bacteremia model. Antimicrob Agents Chemother. (2015) 59:1983–91. 10.1128/AAC.04641-14 25605353 PMC4356752

[323] Thandar M Lood R Winer BY Deutsch DR Euler CW Fischetti VA. Novel engineered peptides of a phage lysin as effective antimicrobials against multidrug-resistant *Acinetobacter baumannii*. Antimicrob Agents Chemother. (2016) 60:2671–9. 10.1128/AAC.02972-15 26856847 PMC4862495

[324] Vukotic G Obradovic M Novovic K Di LM Jovcic B Fira D. Characterization, antibiofilm, and depolymerizing activity of two phages active on carbapenem-resistant *Acinetobacter baumannii*. Front Med. (2020) 7:426. 10.3389/fmed.2020.00426 32974360 PMC7461965

[325] Grygorcewicz B Wojciuk B Roszak M Łubowska N Błazejczak P Jursa-Kulesza J. Environmental phage-based cocktail and antibiotic combination effects on *Acinetobacter baumannii* biofilm in a human urine model. Microb Drug Resist. (2021) 27:25–35. 10.1089/mdr.2020.0083 32543337

[326] Ran B Yuan Y Xia W Li M Yao Q Wang Z. A photo-sensitizable phage for multidrug-resistant *Acinetobacter baumannii* therapy and biofilm ablation. Chem Sci. (2020) 12:1054–61. 10.1039/d0sc04889e 34163871 PMC8179032

[327] Shakeri S Kermanshahi RK Moghaddam MM Emtiazi G. Assessment of biofilm cell removal and killing and biocide efficacy using the microtiter plate test. Biofouling. (2007) 23:79–86. 10.1080/08927010701190011 17453732

[328] Perumal PK Wand ME Sutton JM Bock LJ. Evaluation of the effectiveness of hydrogen-peroxide-based disinfectants on biofilms formed by gram-negative pathogens. J Hosp Infect. (2014) 87:227–33. 10.1016/j.jhin.2014.05.004 24957804

[329] Stowe SD Thompson RJ Peng L Su Z Blackledge MS Draughn GL. Membrane-permeabilizing activity of reverse-amide 2-aminoimidazole antibiofilm agents against *Acinetobacter baumannii*. Curr Drug Deliv. (2015) 12:223–30. 10.2174/1567201811666140924125740 25348099 PMC4640187

[330] Narayanan A Nair MS Karumathil DP Baskaran SA Venkitanarayanan K Amalaradjou MA. Inactivation of *Acinetobacter baumannii* biofilms on polystyrene, stainless steel, and urinary catheters by octenidine dihydrochloride. Front Microbiol. (2016) 7:847. 10.3389/fmicb.2016.00847 27375572 PMC4899441

[331] Burcu S Burak A Yagci AK. Biofilm production and biocidal efficacy in multidrug-resistant *Pseudomonas aeruginosa* and *Acinetobacter baumannii* isolates. Int J Antisepsis Disinfection Sterilization. (2016) 1:7–12. 10.14744/ijads.2016.08208

[332] Runci F Bonchi C Frangipani E Visaggio D Visca P. *Acinetobacter baumannii* biofilm formation in human serum and disruption by gallium. Antimicrob Agents Chemother. (2016) 61:e01563–16. 10.1128/AAC.01563-16 27799219 PMC5192145

[333] Fleeman R Van Horn KS Barber MM Burda WN Flanigan DL Manetsch R. Characterizing the antimicrobial activity of *N*^2^, *N*^4^-Disubstituted quinazoline-2,4-diamines toward multidrug-resistant *Acinetobacter baumannii*. Antimicrob Agents Chemother. (2017) 61:e00059–17. 10.1128/AAC.00059-17 28289036 PMC5444175

[334] Schumm K Lam TB. Types of urethral catheters for management of short-term voiding problems in hospitalized adults: a short version Cochrane review. Neurourol Urodyn. (2008) 27:738–46. 10.1002/nau.20645 18951451

[335] Johnson JR Kuskowski MA Wilt TJ. Systematic review: antimicrobial urinary catheters to prevent catheter-associated urinary tract infection in hospitalized patients. Ann Intern Med. (2006) 144:116–26. 10.7326/0003-4819-144-2-200601170-00009 16418411

[336] Hu X Huang YY Wang Y Wang X Hamblin MR. Antimicrobial photodynamic therapy to control clinically relevant biofilm infections. Front Microbiol. (2018) 9:1299. 10.3389/fmicb.2018.01299 29997579 PMC6030385

[337] Ghorbanzadeh R Assadian H Chiniforush N Parker S Pourakbari B Ehsani B. Modulation of virulence in Enterococcus faecalis cells surviving antimicrobial photodynamic inactivation with reduced graphene oxide-curcumin: an *ex vivo* biofilm model. Photodiagnosis Photodyn Ther. (2020) 29:101643. 10.1016/j.pdpdt.2019.101643 31899382

[338] Geralde MC Leite IS Inada NM Salina AC Medeiros AI Kuebler WM. Pneumonia treatment by photodynamic therapy with extracorporeal illumination - an experimental model. Physiol Rep. (2017) 5:e13190. 10.14814/phy2.13190 28292878 PMC5350187

[339] Granick MS Paribathan C Shanmugam M Ramasubbu N. Direct-contact low-frequency ultrasound clearance of biofilm from metallic implant materials. Eplasty. (2017) 17:e13. 28405263 PMC5372756

[340] Ng E Lim LP. An overview of different interdental cleaning aids and their effectiveness. Dent J. (2019) 7:56. 10.3390/dj7020056 31159354 PMC6630384

[341] Li X Sun L Zhang P Wang Y. Novel approaches to combat medical device-associated biofilms. Coatings. (2021) 1:294. 10.3390/coatings11030294

[342] Li H Fairfax MR Dubocq F Darouiche RO Rajpurkar A Thompson M. Antibacterial activity of antibiotic coated silicone grafts. J Urol. (1998) 160:1910–3. 9783984

[343] Nielsen CK Subbiahdoss G Zeng G Salmi Z Kjems J Mygind T. Antibacterial isoeugenol coating on stainless steel and polyethylene surfaces prevents biofilm growth. J Appl Microbiol. (2018) 124:179–87. 10.1111/jam.13634 29119696

[344] John T Rajpurkar A Smith G Fairfax M Triest J. Antibiotic pretreatment of hydrogel ureteral stent. J Endourol. (2007) 21:1211–6. 10.1089/end.2007.9904 17949328

[345] Piozzi A Francolini I Occhiaperti L Di Rosa R Ruggeri V Donelli G. Polyurethanes loaded with antibiotics: influence of polymer-antibiotic interactions on *in vitro* activity against *Staphylococcus epidermidis*. J Chemother. (2004) 16:446–52. 10.1179/joc.2004.16.5.446 15565910

[346] Roy S Chatterjee S Bhattacharjee A Chattopadhyay P Saha B Dutta S. Overexpression of efflux pumps, mutations in the pumps' regulators, chromosomal mutations, and AAC(6')-Ib-cr are associated with fluoroquinolone resistance in diverse sequence types of neonatal septicaemic *Acinetobacter baumannii*: a 7-year single center study. Front Microbiol. (2021) 12:602724. 10.3389/fmicb.2021.602724 33776950 PMC7990795

[347] Sands K Carvalho MJ Portal E Thomson K Dyer C Akpulu C. Characterization of antimicrobial-resistant gram-negative bacteria that cause neonatal sepsis in seven low- and middle-income countries. Nat Microbiol. (2021) 6:512–23. 10.1038/s41564-021-00870-7 33782558 PMC8007471

[348] Al Atrouni A Joly-Guillou ML Hamze M Kempf M. Reservoirs of non-baumannii acinetobacter species. Front Microbiol. (2016) 7:49. 10.3389/fmicb.2016.00049 26870013 PMC4740782

[349] Tyers M Wright GD. Drug combinations: a strategy to extend the life of antibiotics in the 21st century. Nat Rev Microbiol. (2019) 17:141–55. 10.1038/s41579-018-0141-x 30683887

[350] Asahara T Takahashi A Yuki N Kaji R Takahashi T Nomoto K. Protective effect of a synbiotic against multidrug-resistant *Acinetobacter baumannii* in a murine infection model. Antimicrob Agents Chemother. (2016) 60:3041–50. 10.1128/AAC.02928-15 26953197 PMC4862511

[351] Parra Millán R Jiménez Mejías ME Sánchez Encinales V Ayerbe Algaba R Gutiérrez Valencia A Pachón Ibáñez ME. Efficacy of lysophosphatidylcholine in combination with antimicrobial agents against *Acinetobacter baumannii* in experimental murine peritoneal sepsis and pneumonia models. Antimicrob Agents Chemother. (2016) 60:4464–70. 10.1128/AAC.02708-15 27161639 PMC4958192

[352] Konstantinidis T Kambas K Mitsios A Panopoulou M Tsironidou V Dellaporta E. Immunomodulatory role of clarithromycin in *Acinetobacter baumannii* infection via formation of neutrophil extracellular traps. Antimicrob Agents Chemother. (2015) 60:1040–8. 10.1128/AAC.02063-15 26643338 PMC4750671

